# Childhood experiences and adult prayer or meditation in 22 countries around the world

**DOI:** 10.1038/s41598-025-99796-x

**Published:** 2025-04-30

**Authors:** Matt Bradshaw, Victor Counted, Tim Lomas, Robert D. Woodberry, Tyler J. VanderWeele, Byron R. Johnson

**Affiliations:** 1 Institute for Studies of Religion, Baylor University, One Bear Place #97326, Waco, TX 76798 USA; 2 Department of Psychology, Regent University, Virginia Beach, Virginia, VA USA; 3 Human Flourishing Program, Institute for Quantitative Social Science, Harvard University, Cambridge, MA England; 4 Department of Biostatistics, Department of Epidemiology, Harvard T.H. Chan School of Public Health, Boston, MA USA; 5 School of Public Policy, Pepperdine University, Malibu, CA USA

**Keywords:** Religion, Spirituality, Culture, Cross-National, Development, Life course, Socialization, Childhood, Early-Life, Christianity, Islam, Judaism, Buddhism, Hinduism, Risk factors, Epidemiology

## Abstract

Few studies have examined how childhood experiences and characteristics shape daily prayer or meditation in adulthood across countries around the world using nationally-representative data. The current study addresses this limitation by analyzing data from the Global Flourishing Study (GFS), an international survey of 202,898 individuals from 22 geographically, economically, and culturally diverse countries collected in 2022–2023. Father-child relations, early-life religious attendance, year of birth, and gender were associated with adult daily prayer or meditation in a meta-analysis of all 22 countries. Parental marital status, economic conditions, abuse and adversity, health, and immigration status were not significant in the pooled analysis, but were important in one or more countries. All childhood characteristics showed some variation across nations. E-values suggested that the strongest associations were robust against confounding from unmeasured covariates. Cross-national research is difficult for many reasons including language barriers, norms regarding talking about sensitive issues, and survey question translation and interpretation issues. Despite these, findings show meaningful associations between numerous childhood characteristics and adult prayer or meditation around the world. Results also reveal considerable variation across countries and cultures. This work lays the foundation for future longitudinal GFS studies on the causes and correlates of prayer or meditation in a global context.

**Supplementary Information:**

The online version contains supplementary material available at 10.1038/s41598-025-99796-x.

## Introduction

Foundational experiences during childhood shape the contours of adult life^1,2,3^, but the pathways from early experiences to adult religiosity and spirituality (R/S) remain insufficiently mapped in the scientific literature^4,5^. Considerable research has explored the causes and correlates of adult R/S^6–9^, but childhood antecedents have received far less attention. Previous research has identified aspects of family and social life including maltreatment^4^, attachment^5^, and parent and peer religiosity^10^, but these do not account for the breadth of childhood experiences, which also include family structure (e.g., one or two parent families, divorce), socioeconomic status, health, and immigration experiences, among others. Further, most of the research in this area has been conducted in the United States where Christianity is the dominant religion, and studies across countries and cultures remain rare.

The current study focuses on prayer and meditation, where prayer is commonly understood as the act of communicating with a deity or the divine, expressing thoughts and feelings, or seeking guidance^11,12^, and meditation is often viewed as a practice of focused attention and awareness, including techniques aimed at cultivating compassion, transcendental experiences, or self-transcendence^12^. In Christian, Muslim, and Jewish traditions, meditation may also emphasize an intimate, personal relationship with God. Both share characteristics such as quietude and introspection, but they may differ with respect to goals and a theistic focus^13^. There is considerable overlap between these concepts in countries around the world, however, and they often serve similar religious, spiritual, and reflective functions. Therefore, this study treats them as a broad, unifying construct that refers to a variety of practices across cultures that might be encompassed by prayer or meditation (P/M). These are common behaviors for adherents of many of the world religions including Buddhism, Christianity, Hinduism, Islam, and Judaism, so they are important for diverse individuals and groups around the globe.

Numerous strands of research offer insight into the role that childhood factors may play in shaping P/M in adulthood. From a life course perspective, human development is a lifelong process, and individuals are embedded in powerful social contexts that shape their behavior beginning at conception and continuing throughout life^14,15^. All adult outcomes, including P/M, must therefore be understood in the context of early-life experiences, where habits, practices, relationships, commitments, and beliefs are initially formed, and then reverberate over time^16,17^. Research in this area is often framed in terms of stability/instability, access to resources, and adversity during childhood, all of which may promote or hinder many aspects of human development including R/S^18–22^. Several key domains have been identified including family relationships and structure, economic conditions, abuse and neglect, health and well-being, immigration, childhood religious or spiritual involvement, birth cohort, and gender-specific experiences.

Family relations are likely to have a strong influence on the intergenerational transmission of R/S. Research suggests that warm, supportive, and stable parent-child interactions foster an environment conducive to the adoption of parental religious or spiritual values, including engagement in P/M^21,23^. Other aspects of the family environment, such as marital status and family structure, may also play an important role in religious socialization during childhood. For example, studies have found that children from stable and religiously homogeneous families are more likely to inherit and sustain R/S into adulthood^24,25^. Conversely, family instability or a lack of religious participation early in life may lead to lower levels of religiosity or spirituality in adulthood. Very little research, however, has examined these possibilities in different nations and cultures around the world, and for P/M specifically.

Financial conditions during childhood may be associated with adult R/S as well, in part because they shape stability, available resources, and exposure to stressful conditions and adversity. In his seminal work on religious coping, Pargament found that low-income individuals were more likely than their high-income counterparts to use religion as a mechanism for coping with life’s difficulties^26^. Religious coping can be both positive (adaptive) and negative (maladaptive), however, so it is important to understand exactly how individuals are utilizing religion in their lives^27^. Consistent with Pargament’s findings, a more recent study reported that the prayer was higher among individuals with less income^28^One of the most detailed studies to date replicated these findings for the quantity, style, purpose, and target of prayer^29^. These studies examined the association between financial status and prayer among adults using cross-sectional data, so it is not clear whether childhood socioeconomic status is associated with P/M in adulthood. This is especially true in countries outside of the developed world, and in nations that are not predominantly or historically Christian.

Experiencing abuse or an outsider status during childhood may lead to complex outcomes regarding R/S. Some individuals may turn to P/M as a coping mechanism for dealing with adversity^26^, seeking solace and healing, and others may distance themselves from religious or spiritual practices that they associate with traumatic contexts or find objectionable for personal reasons (e.g., homophobia, traditional gender roles)^30^. Further, it is possible that R/S may promote resilience in the face of instability, stress, and suffering^31^. In a review of research on childhood abuse and R/S, Walker et al.^32^found that the majority of studies reported either declines in R/S or a combination of both increases and decreases^4,33^. Very little work, however, has focused specifically on P/M even though this aspect of religious and spiritual life may offer promise in promoting posttraumatic growth^34^and better health^35^. Further, relatively little research on this topic has been conducted outside of developed, Western, and predominantly Christian nations, although at least one study has examined the connection between childhood trauma and spirituality among non-religious respondents^36^.

Considerable research, including a growing number of longitudinal studies, shows that multiple aspects of R/S are associated with better health and well-being, in part because religion may promote stable relationships, offer resources to participants, and provide mechanisms for dealing with hardship^7,37^. It is possible, however, that health shapes R/S as well. A recent study provided support for a bidirectional, reciprocal relationship between religiosity and health^38^. Specifically, the authors found that religious activities were associated with improvements in self-rated health over time. Their findings also revealed that poor health predicted increases in both religious activities and conservative religious beliefs across three waves of data. These findings were limited to Christians in the United States, and it is not clear whether this trend will replicate among other religious traditions and in different nations around the world such as Islam in the Middle East or Buddhism in parts of Asia. Further, this type of research has not been conducted specifically on P/M, so the findings reported here will provide new insights for many countries.

Immigration status may also play a role, and several studies have documented the importance of R/S in the lives of immigrants^39,40^. Previous research suggests that migrants may draw on R/S when facing adversity in their new countries^41^, and that religion may help them acculturate^42^. Other work suggests that religiosity may be higher among unemployed, less educated, and recent immigrants^43^, perhaps because they are drawing on R/S as a coping mechanism to deal with unstable living environments and the stress of migration^44^. It may also be the case that migration from religious to secular nations is more common than the opposite scenario. Most of the studies in this area have focused on religious affiliation and service attendance, and very few have examined private aspects of R/S such as prayer or meditation^43^. Thus, we know very little about the private religious and spiritual lives of immigrants compared with native-born individuals, especially around the world.

Extensive research has examined the intergenerational transmission of R/S^45,46^. Drawing on social capital theory, this work suggests that religious interactions with parents shape the religious practices and beliefs of children during adolescence, and that early habituation into R/S ingrains these behaviors, making them more likely to persist into adulthood^47,48^. At least two studies suggests that grandparents may play a role above and beyond parents during the religious socialization process^45,49^. This literature is weak in at least three ways, however: (a) the vast majority of research has focused on predominantly or historically Christian nations; (b) most work has been done in developed, Western nations, particularly the United States; and (c) we know very little about the intergenerational transmission of prayer or meditation since most research has focused on organizational involvement and beliefs. It is likely, however, that religious and spiritual participation early in life will shape P/M practices into adulthood. Therefore, research on P/M in different nations around the world is needed.

Finally, two key demographic factors—year of birth and gender—may shape P/M in adulthood because of their connections to social stability, resources, and adversity. Many different factors related to year of birth are relevant to R/S including economic conditions at the time, political stability, peace and conflict, educational opportunities, and media influences, among many other factors that vary across time and place^50^. Research using age-period-cohort techniques suggests that age is positively associated with religious involvement in societies around the world, and that period changes related to increasing national wealth are negatively correlated with religiosity^51^. According to this study: “These results indicate that both individual aging processes and changes in the material environment may influence changes in religious involvement, in keeping with both psychological and sociological theory, but that culture also plays a role in the nature and speed of these changes (p.979).” Numerous studies also suggest that women may be more religious than men, and this has been attributed to differential socialization, psychological characteristics, and biological/evolutionary processes^52,53^. This gender gap may originate early in life, where children are often socialized differently based on their gender, potentially influencing their adult R/S routines. There are gender differences in many related outcomes as well including personality, cognitive processes, emotions, and social behaviors such as aggression, competition, and leadership^54^, and these factors may contribute to gender differences in R/S. However, much of this research concerns the West, and overall, we know relatively about how age and gender shape P/M in different countries and cultures around the world, and additional research is warranted.

The current study builds on these insights and expands our knowledge in these areas by examining the associations between several childhood experiences and characteristics—i.e., parent child relations, family structure early in life, family income, abuse, feeling like an outsider, health status, immigration, religious service attendance, year of birth, and gender—and adult P/M using data from more than 200,000 participants in 22 diverse countries around the world in the Global Flourishing Study (GFS). Three main research questions guide this study. Research Question 1: Are these childhood characteristics associated with P/M in adulthood? Given the literature reviewed above, good parent-child relations and growing up in a stable family environment, good health, childhood religious service attendance, belonging to an older birth cohort, and female gender are all expected to have positive associations with P/M in adulthood. In contrast, childhood financial conditions, abuse, social adversity, and immigration status are likely to have complex associations with adult P/M, and may have either positive or negative relationships. On one hand, stressful conditions such as financial hardship, abuse, social exclusion, and migration may lead to coping responses in the form of P/M. On the other hand, people without these types of adversity may be thankful for their blessings, and therefore respond with prayer or meditation centered on gratitude. Research Question 2: Do these associations vary by country? Each of the childhood variables is expected to have meaningful correlations with daily P/M in adulthood, but the strength of the associations is likely to vary across countries in response to local cultures and contexts. Research Question 3: Are the observed relationships robust to potential unmeasured confounding?

## Methods

The description of the methods below has been adapted from VanderWeele, Johnson, et al.^55^. Further methodological detail is available elsewhere^56,57,58,59,60^.

### Data

Data come from Wave 1 of the GFS, a longitudinal panel study with an intended five waves of data collection, which is designed to examine the distribution and determinants of well-being across a sample of 202,898 participants from 22 geographically, economically, and culturally diverse countries. Wave 1 collected nationally-representative data from the following countries and territories: Argentina, Australia, Brazil, Egypt, Germany, Hong Kong (Special Administrative Region of China, with mainland China also included from 2024 onwards), India, Indonesia, Israel, Japan, Kenya, Mexico, Nigeria, the Philippines, Poland, South Africa, Spain, Sweden, Tanzania, Turkey, the United Kingdom, and the United States. These countries were chosen to: (a) maximize coverage of the world’s population; (b) ensure geographic, cultural, and religious diversity; and (c) prioritize feasibility and existing data collection infrastructure. Gallup Inc. conducted the data collection primarily in 2023, although some regions began in 2022; timing varied by country, and more information can be found elsewhere^60^. The precise sampling design to ensure nationally-representative samples varied by country^60^. Plans are in place to collect four additional waves of annual panel data on the participants from 2024 to 2027. The data are publicly available through COS (https://www.cos.io/gfs). The translation process followed the TRAPD model (translation, review, adjudication, pretesting, and documentation) for cross-cultural survey research (ccsg.isr.umich.edu/chapters/translation/overview). Additional details are available in the GFS Questionnaire Development Report^56^, Methodology^60^, Codebook (https://osf.io/cg76b), and Translations documents^57^.

## Measures

### Outcome variable

P/M was measured with a single indicator. Respondents were asked the following question: “How often do you pray or meditate?” Response categories were 1 = more than once a day, 2 = about once a day, 3 = sometimes, and 4 = never. A dichotomous variable coded 1 if the respondents reported more than once a day or about once a day and 0 if they reported sometimes or never was constructed and used in all analyses. This dichotomization was chosen on substantive grounds to provide a meaningful, simplified response scale for easy comparison of coordinated methods and findings across GFS papers on numerous topics^59^.

## Predictor variables

Relationship with one’s mother during childhood was assessed with the question: “Please think about your relationship with your mother when you were growing up. In general, would you say that relationship was very good, somewhat good, somewhat bad, or very bad?” A dichotomous variable for very/somewhat good (coded 1) versus very/somewhat bad (coded 0) was constructed. An analogous variable was used for relationship with father. Does not apply was treated as a dichotomous control variable coded 1 for respondents who did not have a mother or father due to death or absence, and coded 0 otherwise.

Parental marital status during childhood was assessed with a series of dummy variables representing married (reference group), divorced, never married, and one or both had died.

Family financial status was measured with the following question: “Which one of these phrases comes closest to your own feelings about your family’s household income when you were growing up, such as when you were around 12 years old?” A series of dummy variables was created for lived comfortably, got by (reference group), found it difficult, and found it very difficult.

Abuse was assessed with yes/no responses (coded 1 and 0, respectively) to: “Were you ever physically or sexually abused when you were growing up?”

Participants were separately asked: “When you were growing up, did you feel like an outsider in your family?” This variable was also coded 1 for yes and 0 for no.

Childhood health was assessed by: “In general, how was your health when you were growing up?” A series of dummy variables indicating excellent, very good, good (reference group), fair, and poor was constructed.

Immigration status was assessed with: “Were you born in this country, or not?” This was coded 1 for yes and 0 for no.

Religious attendance during childhood was assessed with: “How often did you attend religious services or worship at a temple, mosque, shrine, church, or other religious building when you were around 12 years old?” A series of dummy variables indicating at least once/week, one-to-three times/month, less than once/month, and never (reference group) was constructed.

A continuous age variable was collapsed into the following series of dummy variables: 18–24 (reference group), 25–29, 30–39, 40–49, 50–59, 60–69, 70–79, and 80 or older.

Gender was assessed with a series of dummy variables for male (reference group), female, and other gender identity.

Childhood religious tradition was measured with a series of dummy variables indicating: Christianity, Islam, Hinduism, Buddhism, Judaism, Sikhism, Baha’i, Jainism, Shinto, Taoism, Confucianism, Primal/Animist/Folk religion, Spiritism, African-Derived, some other religion, or no religion/atheist/agnostic. Response categories varied by country^57^. When the category no religion/atheist/agnostic had more than 5% of the within country sample size, this was used as the reference category; otherwise, the most prominent religious group was used. Additionally, all religious categories endorsed by less than 3% of the within country sample size were collapsed into a single religious category.

Race/ethnic identity was coded 1 if the individual was in the most prominent group compared with 0 if they were a member of a minority group (race plurality).

## Analytic strategy

A weighted modified Poisson regression model with complex survey adjusted standard errors was fit within each country for P/M on all of the aforementioned childhood variables simultaneously. A Wald-type test was conducted to obtain a global (joint) test of the effect of all categories within a childhood characteristic resulting in a single global p-value of the effect of each childhood variable. In the primary analysis, a random effects meta-analysis of the regression coefficients was estimated^61,62^along with confidence intervals, lower and upper limits 95% prediction intervals, heterogeneity (τ), and I^2^for evidence concerning variation within a given demographic category across countries^63^. Forest plots of estimates are available in the Online Supplement. Religious tradition and race/ethnicity were used within country as control variables when available, but these coefficients themselves were not included in the meta-analyses because categories/responses varied by country. The meta-analysis was conducted in R using the metafor package^64^. Within each country, a global test of association of each childhood predictor variable group with P/M was conducted, and a pooled p-value across countries was reported concerning evidence for association within any country^65^. Bonferroni corrected p-value thresholds were provided based on the number of childhood demographic variables^66,67^. For each childhood variable, E-values were calculated to evaluate the sensitivity of results to unmeasured confounding. An E-value is the minimum strength of the association an unmeasured confounder must have with both the outcome and the predictor, above and beyond all measured covariates, for an unmeasured confounder to explain away an association^68^. As a supplementary analysis, a population weighted meta-analysis of the regression coefficients was estimated. All analyses were pre-registered with COS prior to data access, with only slight subsequent modification in the regression analyses due to multicollinearity on some of the parent variables in some countries with small sample sizes. For example, we originally intended to include separate variables for parent-child relationship quality, feeling love from parents, and parental religious service attendance during childhood, but due to multicollinearity issues, we only retained relations with both mother and father, as well as marital status. Additional details are provided elsewhere^59^. Code to reproduce the analyses is openly available in an online repository^59^.

### Missing data

Missing data on all variables was imputed using multivariate imputation by chained equations, and five imputed datasets were used^69,70^. The percentage of missing data was low (< 5% for all variables), and the item with the largest percent missing was parent marital status (4.9%)^59^. Less than 1% was missing/imputed on the outcome variable. To account for variation in the assessment of certain variables across countries (e.g., religious tradition and race/ethnicity), the imputation process was conducted separately in each country. This within-country imputation approach ensured that the imputation models accurately reflected country-specific contexts and assessment methods. Sampling weights were included in the imputation models to account for specific-variable missingness that may have been related to the probability of inclusion in the study.

## Accounting for complex sampling design

The GFS used different sampling designs across countries based on availability of existing panels and recruitment needs^60^. All analyses accounted for the complex survey design components by including weights, primary sampling units, and strata. Additional methodological detail, including accounting for the complex sampling design, is provided elsewhere^59,71^.

## Results

### Descriptive statistics

Table 1 presents nationally-representative descriptive statistics for the full sample. The majority of participants reported having very good relationships with their mother (63%) and father (53%). Most participants grew up in households where their parents were married (75%), 35% indicated that their families lived comfortably, 14% reported experiencing childhood abuse and feeling like an outsider, 33% rated their childhood health as excellent (31% as very good), 94% were born in the country they currently reside in, and 41% attended services at least once a week during childhood. The sample was diverse in terms of age and the gender distribution was nearly even (49% male and 51% female, along with a small percentage of other gender identities).

**Table 1:** Nationally-Representative descriptive statistics of the observed sample*.

Variable	Proportion	Frequency
Relationship with Mother		
Very Good	0.63	127,836
Somewhat Good	0.26	52,439
Somewhat Bad	0.05	11,060
Very Bad	0.02	4642
Not Applicable	0.03	5965
Missing	0.00	956
Relationship with Father		
Very Good	0.53	107,742
Somewhat Good	0.27	55,714
Somewhat Bad	0.08	15,807
Very Bad	0.04	8278
Not Applicable	0.07	13,985
Missing	0.01	1372
Parent Marital Status		
Married	0.75	152,001
Divorced	0.09	17,726
Never Married	0.08	15,534
One or Both Had Died	0.04	7794
Missing	0.05	9843
Childhood Income		
Lived Comfortably	0.35	70,861
Got By	0.41	82,905
Found it Difficult	0.18	35,852
Found it Very Difficult	0.06	12,606
Missing	0.00	674
Childhood Abuse		
Yes	0.14	29,139
No	0.82	167,279
Missing	0.03	6479
Outsider		
Yes	0.14	28,732
No	0.84	170,577
Not Applicable	0.01	2712
Missing	0.00	877
Childhood Health		
Excellent	0.33	67,121
Very Good	0.31	63,086
Good	0.23	47,378
Fair	0.10	19,877
Poor	0.02	4906
Missing	0.00	530
Immigration Status		
Born in This Country	0.94	190,998
Born in Another Country	0.05	9791
Missing	0.01	2110
Childhood Service Attendance		
At Least 1/Week	0.41	83,237
1–3/Month	0.16	33,308
<1/Month	0.18	36,928
Never	0.23	47,445
Missing	0.01	1980
Year of Birth		
1998–2005; Age 18–24	0.13	27,007
1993–1998; Age 25–29	0.10	20,700
1983–1993; Age 30–39	0.20	40,256
1973–1983; Age 40–49	0.17	34,464
1963–1973; Age 50–59	0.16	31,793
1953–1963; Age 60–69	0.14	27,763
1943–1953; Age 70–79	0.08	16,776
1943 or Earlier; 80 or Older	0.02	4119
Missing	0.00	20
Gender		
Male	0.49	98,411
Female	0.51	103,488
Other	0.00	602
Missing	0.00	397
Country		
Argentina	0.03	6724
Australia	0.02	3844
Brazil	0.07	13,204
Egypt	0.02	4729
Germany	0.05	9506
Hong Kong	0.01	3012
India	0.06	12,765
Indonesia	0.03	6992
Israel	0.02	3669
Japan	0.10	20,543
Kenya	0.06	11,389
Mexico	0.03	5776
Nigeria	0.03	6827
Philippines	0.03	5292
Poland	0.05	10,389
South Africa	0.01	2651
Spain	0.03	6290
Sweden	0.07	15,068
Tanzania	0.04	9075
Turkey	0.01	1473
United Kingdom	0.03	5368
United States	0.19	38,312

*Country-specific descriptive statistics are available in the Online Supplement.

### Meta-Analysis results: childhood characteristics and adult P/M

The meta-analysis explored the relationships between each of the childhood characteristics and daily P/M in adulthood across all 22 countries combined (see Table 2). Having a very/somewhat good relationship with one’s father was associated with a slightly higher daily prayer or meditation in adulthood (RR = 1.03, 95% CI: 1.01, 1.05) compared with a bad/somewhat bad relationship. The effect size was small so these results should not be overstated. Relations with one’s mother were weak. The strongest association was observed for early-life religious service attendance. Participants who attended at least once a week during childhood were substantially more likely to engage in P/M as adults (RR = 1.91, 95% CI: 1.51, 2.42). Similarly, those who attended services 1–3 times per month (RR = 1.64, 95% CI: 1.33, 2.01) or less than once a month (RR = 1.21, 95% CI: 1.10, 1.34) also showed a higher likelihood of engaging in these practices. Daily P/M also tended to be more common among older cohorts compared with younger ones, and there was a somewhat linear relationship with year of birth. Compared with those born in 1998–2005, the RR for all cohorts except 1993–1998 were larger than one, ranging from 1.08 to 1.48. Gender was also a key factor, with an RR of 1.14 (95% CI: 1.09, 1.20) for women compared with men, highlighting an important gender difference across the analyzed countries.

Overall, these results suggest a lasting impact of father-child relations, childhood service attendance, birth cohort, and gender on adult P/M. Other factors like parental marital status, income, abuse and adversity, health, and immigration status may play less of a role. However, these findings mask significant associations between most of these variables in one or more countries, as indicated by the global p-values. The I^2^ values provide additional evidence that many of these associations vary across nations. Results for each individual country are provided in Tables S1-S22, and summarized in detail in the Discussion below.

**Table 2:** Random effects Meta-Analysis of regression of daily prayer or meditation on childhood predictors.

				Estimated Proportion of Effects by Threshold			
Variable	Category	RR	95% CI	< 0.90	> 1.10	I^2	Global p-value
Relationship with mother	(Ref: Very bad/somewhat bad)						0.738
	Very/somewhat good	1.02	(1.00,1.05)	0.00	0.00	< 0.1ǂ	
Relationship with father	(Ref: Very bad/somewhat bad)						0.142
	Very/somewhat good	1.03	(1.01,1.05)	0.00	0.00	2.6	
Parent marital status	(Ref: Parents married)						< 0.001**
	No, divorced	1.00	(0.94,1.05)	0.18	0.14	77.6	
	Single, never married	1.02	(0.95,1.10)	0.14	0.36	86.4	
	No, one or both had died	1.01	(0.94,1.09)	0.14	0.18	89.0	
Subjective financial status of family growing up	(Ref: Got by)						< 0.001**
	Lived comfortably	1.01	(0.99,1.03)	0.00	0.00	53.1	
	Found it difficult	1.01	(0.99,1.03)	0.00	0.00	43.9	
	Found it very difficult	1.01	(0.97,1.05)	0.05	0.14	64.3	
Abuse	(Ref: No)						0.001**
	Yes	1.02	(0.97,1.08)	0.00	0.24	89.0	
Outsider growing up	(Ref: No)						< 0.001**
	Yes	1.05	(0.99,1.11)	0.05	0.32	90.5	
Self-rated health growing up	(Ref: Good)						< 0.001**
	Excellent	1.03	(0.99,1.08)	0.09	0.18	89.8	
	Very good	1.01	(0.98,1.04)	0.00	0.09	73.6	
	Fair	0.99	(0.94,1.04)	0.09	0.05	85.6	
	Poor	1.10	(0.98,1.24)	0.05	0.36	91.7	
Immigration status	(Ref: Born in this country)						< 0.001**
	No	1.09	(0.99,1.20)	0.14	0.41	82.7	
Age 12 religious service attendance	(Ref: Never)						< 0.001**
	At least 1/week	1.91	(1.51,2.42)	0.00	0.91	99.2	
	1–3/month	1.64	(1.33,2.01)	0.00	0.77	98.6	
	Less than 1/month	1.21	(1.10,1.34)	0.00	0.55	92.2	
Year of birth	(Ref: 1998–2005; age 18–24)						< 0.001**
	1993–1998; age 25–29	1.04	(1.00,1.07)	0.00	0.14	64.5	
	1983–1993; age 30–39	1.08	(1.02,1.15)	0.09	0.32	92.6	
	1973–1983; age 40–49	1.13	(1.04,1.23)	0.09	0.55	95.6	
	1963–1973; age 50–59	1.24	(1.12,1.37)	0.00	0.77	96.6	
	1953–1963; age 60–69	1.29	(1.13,1.46)	0.05	0.77	97.5	
	1943–1953; age 70–79	1.35	(1.17,1.57)	0.05	0.82	94.7	
	1943 or earlier; age 80+	1.48	(1.30,1.68)	0.00	0.86	89.6	
Gender	(Ref: Male)						< 0.001**
	Female	1.14	(1.09,1.20)	0.00	0.64	95.7	
	Other	0.18	(0.02,1.54)	0.22	0.50	100.0	

Note. *p <.05; **p <.004 (Bonferroni corrected threshold); ǂestimate of heterogeneity is likely unstable, please see our online supplement forest plots for more detail on heterogeneity of effects.

### Sensitivity analyses

Table 3 shows E-values and E-value limits for the regression findings shown in Table 2. These results suggest that some relationships are quite robust to unmeasured confounding. Variables including childhood religious service attendance, year of birth, and gender had E-values of 1.50 or higher. For example, to explain away the association between weekly childhood attendance and adult P/M (RR = 1.91), an unmeasured confounder associated with both variables by risk ratios of 3.22 each (above and beyond all other covariates included in the model) could suffice, but weaker joint confounder associations could not. Additionally, in order to explain away the confidence interval for this association, an unmeasured confounder associated with both service attendance and adult prayer/mediation by risk ratios of 2.38 each could shift the confidence interval to include the null, but weaker joint confounder associations could not. In contrast, other variables including parent-child relations, parental marital status, financial conditions, abuse and feeling like an outsider, health, and immigration status had smaller E-values, and may therefore be more susceptible to confounding. Many of these associations were weak or not significant.

As an additional sensitivity analysis and alternative to the primary random effects meta-analysis, Table S23 shows results from a fixed effects meta-analysis using 2023 population size weights reflecting differences in the number of people in each country. In other words, this analysis was weighted by individuals in each country rather than by country, which elevated the influence of more populous countries such as India. Compared with Table 2, the findings for most variables were similar, although the specific estimates varied. The effects of abuse and outsider status were slightly stronger in the population-weighted findings, but the overall pattern of findings remained somewhat consistent with the primary analyses, suggesting that the findings are fairly robust. E-values for these findings are available in Table S24.

**Table 3:** Sensitivity of Meta-Analyzed childhood predictors to unmeasured confounding.

Variable	Category	E-value	E-value Limit
Relationship with mother	(Ref: Very bad/somewhat bad)		
	Very/somewhat good	1.18	1.00
Relationship with father	(Ref: Very bad/somewhat bad)		
	Very/somewhat good	1.20	1.10
Parent marital status	(Ref: Parents married)		
	No, divorced	1.07	1.00
	Single, never married	1.17	1.00
	No, one or both had died	1.13	1.00
Subjective financial status of family growing up	(Ref: Got by)		
	Lived comfortably	1.11	1.00
	Found it difficult	1.10	1.00
	Found it very difficult	1.13	1.00
Abuse	(Ref: No)		
	Yes	1.17	1.00
Outsider growing up	(Ref: No)		
	Yes	1.28	1.00
Self-rated health growing up	(Ref: Good)		
	Excellent	1.22	1.00
	Very good	1.09	1.00
	Fair	1.13	1.00
	Poor	1.43	1.00
Immigration status	(Ref: Born in this country)		
	No	1.40	1.00
Age 12 religious service attendance	(Ref: Never)		
	At least 1/week	3.22	2.38
	1–3/month	2.66	2.00
	Less than 1/month	1.72	1.43
Year of birth	(Ref: 1998–2005; age 18–24)		
	1993–1998; age 25–29	1.23	1.04
	1983–1993; age 30–39	1.37	1.14
	1973–1983; age 40–49	1.52	1.25
	1963–1973; age 50–59	1.79	1.49
	1953–1963; age 60–69	1.90	1.52
	1943–1953; age 70–79	2.05	1.62
	1943 or earlier; age 80+	2.32	1.93
Gender	(Ref: Male)		
	Female	1.55	1.41
	Other	10.79	1.00

## Discussion

According to the life course paradigm, individuals are embedded in influential social contexts from birth until death, and many habits and practices that initially develop early in life remain relevant well into adulthood^14,15,72^. Early-life contexts and experiences provide stability/instability that may have consequences throughout life, and may shape access to key resources and exposure to adversity across time^18,19,20,21,22^. Drawing on this conceptual foundation, the current study examined the associations between childhood experiences and daily P/M in adulthood in 22 diverse countries around the world. Findings from a random effects meta-analysis of all countries combined showed that father-child relations, childhood religious service attendance, birth cohort, and gender were all associated with P/M in adulthood. There was considerable heterogeneity across nations, however, and most of the childhood characteristics examined here were associated with P/M in one or more countries even though some did not show definitive evidence in the pooled meta-analysis. These relationships may be shaped by local social contexts and conditions, so country-specific results are provided in Supplementary Tables S1b-S22b and discussed in detail below.

When looking across countries, parental relationships during childhood were only strongly associated with P/M in one nation: Japan. Findings were also marginally significant in Poland (i.e., significant without a Bonferroni correction). In both cases, having a somewhat/very good relationship with one’s father (but not mother) had a positive association with P/M in adulthood. The findings for parental marital status were somewhat stronger. Compared with married, divorced had positive associations with P/M in India, Japan, and Spain, but a marginally significant negative one in Nigeria. Single/never married was positive in Japan (this was marginally true in Egypt, Poland, Spain and the United States as well), but negative in Kenya and Tanzania (marginal in Mexico). Having a parent who died was positive in India (marginally), Japan, and the Philippines, but marginally negative in Israel and Nigeria. The effect sizes were somewhat larger in Japan and Spain (two economically developed countries) compared with India, Nigeria, the Philippines, and Tanzania. The countries where significant findings were observed were diverse in terms of economic development, region of the world, and religious context, so there were no obvious or clear differences or similarities between countries where marital status mattered and where it did not. It is possible that in situations where less than ideal family structures are present, a positive association with P/M may indicate that individuals are drawing on R/S to alleviate stress or adversity, whereas when the association is negative it could be due to a breakdown in religious socialization processes or turning away from R/S in response to adverse or undesirable experiences^26^. These findings were weaker than expected and there are at least three possible explanations. First, the average age in the sample was roughly 46 years. Considerable time may have passed since childhood for many participants, so the effects of early life parental relations may have diminished. Second, all of the variables were included in the models together, so the effects of the parent-child variables are net of the other variables. Third, the connections between parent religiosity, parent-child relations, and child R/S outcomes may be moderated by gender^73,74^. In other words, parent-child relationship and religious dynamics may differ depending on whether the child shares the same gender as the parent. Examining this possibility was beyond the scope of the current study, but should be priority for future research.

Childhood financial status was not strongly associated in the meta-analysis, but it was correlated with adult P/M in several countries. Compared with getting by financially, living comfortably had a positive association with P/M in India and South Africa (marginally), but a negative relationship in Tanzania. There was also a positive relationship among those who found it difficult to get by on their household income in the United States (marginally), but a negative one in Tanzania. For those who found it very difficult to get by, there was a positive correlation with P/M in Mexico and the United States, and a negative one in Germany. The effect sizes tended to be larger in Germany compared with Mexico, Tanzania, and the United States. Interestingly, P/M was more common among those who suffered financial hardship in the United States and Mexico, but less common in Germany and Tanzania. This could be related to differences in social safety nets or the predominance and influence of religion in each country. It may also be important to note that Christianity is the largest religious tradition in all four countries, so connections between financial status and religion may be stronger in predominately Christian nations compared with others, although null findings were also observed in several countries where Christianity is common. The findings for the United States and Mexico align with previous research showing that childhood adversity, including low socioeconomic status, can have lasting effects on adult religiosity^26,28,29^. This was not observed in many other countries, however, and the effect sizes were relatively small, so these findings should not be overstated. Overall, even though financial conditions early in life may contribute to adult P/M in some contexts, they are likely part of a broader constellation of influences. In some settings, individuals from disadvantaged backgrounds may turn to P/M as a coping mechanism while seeking comfort and hope^26^, or as a source of meaning in life^75,76^. In others, those from more advantaged backgrounds may engage in P/M as a reflection of gratitude or as a continuation of familial traditions.

Previous research suggest that abuse or other forms of adversity during childhood may shape R/S in complex ways, with some individuals possibly turning to practices like P/M to deal with life’s difficulties, and others distancing themselves from R/S due to traumatic events or contexts^26,30,31,32,33^. The results for abuse were not significant in the meta-analysis, but there were important findings in a few countries. Abuse was positively associated with P/M in Australia, Hong Kong, India, Japan, and Sweden (marginally), but negatively in Nigeria and Tanzania (marginally). Effect sizes were larger in Australia, Hong Kong, Japan, and Sweden (all developed nations), and lower in India, Nigeria, and Tanzania (developing nations). Feeling like an outsider was positively associated with P/M in Honk Kong (marginally), Japan, Sweden, and the United Kingdom, but negatively in the United States. Effect sizes were larger in Japan, Sweden, and the United Kingdom, and smaller in Hong Kong and the United States. For both variables, positive associations occurred in countries with high levels of secularization or where a non-Abrahamic religious tradition predominates (e.g., Hinduism, Buddhism, Shinto, and folk religion), and negative associations were observed in less secularized countries where Christians predominate. No significant findings were observed in the remaining countries. The positive associations may be explained, in part, by coping responses, or as sources of resilience, hope, optimism, forgiveness, and meaning^30,31,32,33,76,77,78^. Further, extensions of attachment theory to religious outcomes suggest that relationships with God, gods, or other transcendent forces may compensate for poor connections to other individuals including parents^79,80^, which implies that individuals who experience abuse or who are not close to others in their families may seek connections with religious or spiritual others to compensate for these worldly deficiencies. Relatively few studies to date have specifically focused on childhood adversity and P/M, however, even though this aspect of religious and spiritual life may offer unique promise for coping and well-being because it is readily available to individuals regardless of region of the world, social status, health, and other factors that may limit access to alternative resources^34,35^.

Numerous studies have linked R/S with health and well-being^7,37^. Most of this work has focused on the role of R/S in shaping health, but this relationship may work in the opposite direction as well^38^. Even though the results of the meta-analysis were not significant, the current findings suggest that childhood health has complex associations with P/M across countries. Compared with good health, excellent or very good health had positive relationships with P/M in Israel, Japan, the Philippines, Spain, and Tanzania (marginally), but negative ones in Egypt, Germany (marginally), Kenya (marginally), and Poland. Fair or poor health had positive associations with P/M in Argentina, Israel, Japan, the United Kingdom (marginally), and the United States, but a negative one in Hong Kong. Poor health had the strongest positive association with health in Israel compared with Argentina, Japan, and the United States. Importantly, the countries where significant relationships were observed were diverse in terms of economic development, region of the world, and religious context, so there are no clear similarities among countries where poor health is linked with P/M. In situations where good health appears to promote P/M (i.e., in Israel, Japan, the Philippines, Spain, and Tanzania), this could be linked with gratitude or related characteristics^81,82^, whereas in settings where P/M is lower among healthy individuals (i.e., Hong Kong), this may be due to living a comfortable life with no real or perceived need for supernatural or spiritual support. It is important to note that childhood health was assessed retrospectively. Future research using prospective data should examine these associations since recall bias may be present in the current findings.

An association between immigration status and R/S has been documented in previous research^39^. Here, there were relatively weak findings in the meta-analysis and the majority of countries, but there were positive associations between being an immigrant (compared with native born) and P/M in Germany, Japan, Spain, Sweden, Tanzania, and the United Kingdom (marginally). In contrast, the relationship was negative in Israel. Effect sizes were largest in Germany, Japan, Spain, and Sweden (all economically developed nations), and lowest in Israel, Tanzania, the United Kingdom (a diverse group of countries). Together, these countries are economically, geographically, and religiously diverse, so country-specific contexts net of these characteristics are likely playing a role. The significant positive findings are consistent with research showing that immigrants may draw on R/S when attempting to integrate into their new home countries^41,42,43,83^, and may use it to deal with the stress of immigrating^44^. These findings may also be explained by the fact that these immigrants moved to relatively secular nations like Germany, Japan, Spain, Sweden, and the United Kingdom, and thus may have already been more religious prior to moving to their new homes. Conversely, in Israel, recent immigrants from the United States and former-communist countries in East Europe and Russia may have been more secular than native-born Israelis before they arrived.

Considerable research has examined the intergenerational transmission of R/S^45–49^. However, much of this research has been conducted in developed, Western, and historically Christian countries, and we know very little about the processes that shape adult R/S in many nations around the world. In the GFS, childhood religious service attendance had strong associations with adult P/M in the meta-analysis and all countries except Kenya, Nigeria, and South Africa. However, because religiosity is so high in these countries, there may be a ceiling effect. Effect sizes for weekly attendance were largest in Australia, Germany, Hong Kong, Israel, Japan, Poland, Spain, Sweden, and the United Kingdom (all economically developed nations), and smallest in Egypt, India, Indonesia, the Philippines, and Tanzania (all developing countries). The results corroborate previous findings that childhood religious involvement is a robust predictor of adult R/S, including P/M^84,85,45^. The consistency of this relationship across economically, geographically, and religiously diverse countries suggests a near universal aspect of religious socialization where early habituation to structured R/S practices fosters a lifelong commitment^45^. The variability in the strength of this relationship across countries, however, suggests that national contexts play a significant role in how R/S is transmitted intergenerationally. For instance, the stronger association found in the United Kingdom compared with the United States may reflect the role of religion within these societies. The United Kingdom, being a predominantly secular society, may have a more pronounced effect of religious upbringing due to its relative rarity, but in the United States, where religion is more ingrained in the cultural fabric, the effect might be diluted by other societal factors^86^.

A variety of factors linked with year of birth are relevant to the development of adult P/M as well, including economic contexts, political structures, war and peace, educational opportunities, and the media^50^. In addition, considerable research shows that R/S tends to be higher among older cohorts^51^. Even though there were significant results for all countries combined, year of birth had complex associations with P/M across countries in the current data. Consistent with most previous research, there was a somewhat linear increase in daily P/M as age cohorts increased in Brazil, Egypt, Germany, India, Indonesia, Japan, Kenya, Mexico, South Africa, Tanzania, Turkey, and the United States, a diverse set of countries and cultures. There was a similar pattern in Argentina, the Philippines, and Poland with one exception: the oldest age cohort was not higher than 70–79, and in some cases was lower. In the United Kingdom, there was a somewhat linear decrease as age increased except for the oldest cohort, which was the highest; findings that are likely related to past and present secularization processes that differ across cohorts. Findings were less clear for the remaining countries. Overall, these results may broadly imply secularization or declining religious practices over time or among younger cohorts in numerous countries, but these processes are highly debated^86,87,88^and deserve further research and scrutiny. These findings could be due to age, period, and/or cohort effects, and one study suggests that numerous processes may play a role^51^.

Numerous studies suggest that women may be more religious than men^52,53^, and the current findings for gender were strong. The results of the meta-analysis were significant, and proportions of daily P/M were higher among women compared with men in Argentina, Australia, Brazil, Egypt, Germany (marginally), India, Indonesia, Israel (marginally), Kenya, Mexico, Nigeria, the Philippines, Poland, South Africa, Sweden, Tanzania, Turkey, and the United States (findings were null in Hong Kong, Japan, Spain, and the United Kingdom). These countries are quite diverse in terms of cultural practices, region of the world, economic development, life expectancy, and majority religious tradition. They also include countries that are highly religious and ones that are considered secular by many scholars, as well as countries that have sizeable Christian, Hindu, Jewish, and Muslim populations. Effect sizes were largest in Argentina, Australia, Poland, Turkey, and the United States (a diverse set of countries), and smallest in India, Indonesia, Kenya, Nigeria, and Tanzania (developing nations). Importantly, men were not significantly higher than women in any country. Possible reasons for this gender disparity are multifaceted and may involve: (a) socialization processes (e.g., women may be socialized to prioritize communal and relational aspects of life, which align with R/S); (b) psychological factors (e.g., women may experience higher levels of anxiety or stress, leading them to seek comfort in R/S); and (c) biological/genetic differences or physiological influences (e.g., ​​hormonal variations, such as higher levels of oxytocin, which are linked to bonding and may enhance R/S)^52,53,59^. The consistency across countries underscores the near universal presence of factors that promote gender differences in P/M around the world.

Religious tradition was not included in the meta-analysis, but it was examined in the country-specific results. Affiliation with a religious group (compared with the reference group: no religion/atheist/agnostic) was positively associated with P/M in many countries including Argentina, Australia, Brazil, Germany, Hong Kong, Japan, Spain, Sweden, the United Kingdom, and the United States. This was not true in Poland, however. In some countries, the not religious category was very small, so the largest one served as the reference group instead. In Indonesia and Turkey, other religious groups were negatively associated with P/M compared with Islam. In Nigeria, there was a positive association between being a member of another religious group compared with Christian. Null findings occurred in Egypt, India, Israel, Kenya, Mexico, the Philippines, South Africa, and Tanzania. Race/ethnicity was also examined in the country-specific results, but these findings were somewhat weak. Being a member of a minority group (compared with the largest category) was positively associated with P/M in Argentina, Brazil, the Philippines (marginally), Tanzania, the United Kingdom, and the United States. Research has documented racial/ethnic differences in numerous consequential outcomes including health and well-being^90^, socioeconomic status^91^, and family life^92,93^, with minority groups typically having worse outcomes possibly due to prejudice and discrimination^94,95^. These findings may indicate a religious coping response that accompanies belonging to a minority group. In contrast, there was a negative association between minority status and P/M in India. Data was not available in Germany, Japan, Spain, and Sweden, and there were no significant relationships observed in the remaining countries. Religion and race scholars with expertise in each specific cultural context should carefully examine the findings for each nation and offer insights based on their unique knowledge.

This study has several strengths. First, the sample includes countries that are economically developed and developing, geographically dispersed, and religiously diverse. This means that the findings are applicable to many individuals and groups around the world. Second, all of the survey items were chosen and evaluated by leading scholars around the world, and then pretested by Gallup in each nation. Third, the GFS is a very large survey, with nationally-representative samples from 22 diverse countries, which should offer reasonable estimates of P/M. Many of the countries included in this study have rarely been examined, so the findings provide new insights into variations in P/M around the world.

Several limitations should also be acknowledged. First, the reliance on self-reported measures introduces the possibility of social desirability bias. Second, the childhood exposures were also assessed retrospectively, giving rise to the possibility of recall bias. However, for recall bias to completely explain away the observed associations would require that the effect of adult P/M on biasing retrospective assessments of the childhood predictors would essentially have to be at least as strong as the observed associations themselves^96^, and a few of these were fairly moderate in magnitude. Third, some degree of confounding by unmeasured variables is possible in the models. Although the study used E-values to assess the robustness of the findings, for most of these, given the more modest magnitudes, there remains the possibility that other factors not accounted for in the analysis could influence the observed relationships. Fourth, cross-cultural research is difficult for many reasons including language barriers, different norms regarding speaking about sensitive issues like R/S, and survey question translation and interpretation issues. Gallup conducted extensive pretesting and translation work in an attempt to obtain comparable meanings of survey items across countries, but some words and concepts are difficult to translate. This means that strict and direct comparisons of one country versus another should be made with caution. Fifth, the childhood questions were framed in terms of experiences “around 12 years old” or “when you were growing up” so we do not have data on the exact timing of these experiences. For example, we do not know whether the respondent’s parents were married until age nine and then got divorced, or whether they attended religious services more frequently prior to or after the age of 12. Sixth, this study only analyzes the first wave of GFS data. Data collection for the second wave is ongoing. Seventh, this study only examined the associations between childhood factors and one aspect of R/S: daily prayer or meditation. Other aspects of religious and spiritual life are also important the lives of many individuals around the world.

Moving forward, the cultural variability observed in the associations between childhood characteristics and adult P/M suggests that future research should further explore the role of national contexts in shaping these relationships. Qualitative studies, in particular, could provide insight into the cultural meanings and practices associated with P/M in different societies. The findings also have important implications for interventions aimed at fostering human flourishing through R/S practices. Given the significant influence of childhood religious involvement on adult P/M, efforts to promote R/S during childhood could have long-lasting benefits. Programs that encourage regular participation in religious or spiritual activities from an early age may help instill lifelong habits of P/M that may be used to cope with stress and adversity. The variability in the effects of childhood predictors across countries also highlights the importance of culturally sensitive approaches to promoting R/S. Interventions and programs should be designed with an awareness of the cultural and societal context, ensuring that they resonate with the target population’s values and beliefs.

## Conclusion

Father-child relations, early-life religious service attendance, year of birth, and gender were associated with prayer or meditation in a meta-analysis that combined all 22 countries. Other characteristics such as parental marital status, economic conditions, abuse and adversity, health, and immigration status were not significant in the full sample, but were important in one or more countries. All characteristics showed some variation across national and cultural contexts. These findings highlight the enduring impact of several early-life experiences on adult R/S. Despite limitations such as self-reported data and possible confounding, this study provides insights into the developmental pathways leading to adult P/M, with implications for promoting human flourishing through early religious or spiritual engagement.

## Electronic supplementary material

Below is the link to the electronic supplementary material.

Supplementary Material 1

## Data Availability

Data for Wave 1 of the Global Flourishing Study is available through the Center for Open Science (COS) upon submission of a preregistration (https://doi.org/10.17605/OSF.IO/3 JTZ8), and will be openly available without preregistration when Wave 2 data is released in 2025. For additional information about data access see the COS website (https://www.cos.io/gfs-access-data) or contact them by email (globalflourishing@cos.io). All code to reproduce the analyses are openly available in an online OSF repository (https://doi.org/10.17605/osf.io/vbype).
