# Supplementary material for: Childhood experiences and adult prayer or meditation in 22 countries around the world
Source: Sci Rep. 2025 Apr 30;15:15083. doi: 10.1038/s41598-025-99796-x (PMC12044052; doi:10.1038/s41598-025-99796-x)

**Childhood Experiences and Adult Prayer or Meditation in 22 Countries Around the World**

**Online Supplement**

**Table S1a: Nationally-Representative Descriptive Statistics of the Observed Sample (Argentina)**

| Variable | Proportion | Frequency |
| --- | --- | --- |
| Relationship with Mother |  |  |
| Very Good | 0.66 | 4463 |
| Somewhat Good | 0.21 | 1436 |
| Somewhat Bad | 0.04 | 299 |
| Very Bad | 0.03 | 216 |
| Not Applicable | 0.04 | 273 |
| Missing | 0.01 | 36 |
| Relationship with Father |  |  |
| Very Good | 0.54 | 3612 |
| Somewhat Good | 0.23 | 1537 |
| Somewhat Bad | 0.07 | 440 |
| Very Bad | 0.06 | 401 |
| Not Applicable | 0.10 | 694 |
| Missing | 0.01 | 39 |
| Parent Marital Status |  |  |
| Married | 0.61 | 4110 |
| Divorced | 0.09 | 637 |
| Never Married | 0.20 | 1368 |
| One or Both Had Died | 0.03 | 199 |
| Missing | 0.06 | 410 |
| Childhood Income |  |  |
| Lived Comfortably | 0.30 | 2042 |
| Got By | 0.34 | 2305 |
| Found it Difficult | 0.27 | 1789 |
| Found it Very Difficult | 0.08 | 569 |
| Missing | 0.00 | 19 |
| Childhood Abuse |  |  |
| Yes | 0.19 | 1302 |
| No | 0.78 | 5271 |
| Missing | 0.02 | 151 |
| Outsider |  |  |
| Yes | 0.17 | 1165 |
| No | 0.81 | 5458 |
| Not Applicable | 0.01 | 68 |
| Missing | 0.00 | 33 |
| Childhood Health |  |  |
| Excellent | 0.36 | 2402 |
| Very Good | 0.27 | 1819 |
| Good | 0.27 | 1830 |
| Fair | 0.08 | 505 |
| Poor | 0.02 | 156 |
| Missing | 0.00 | 12 |
| Immigration Status |  |  |
| Born in This Country | 0.94 | 6346 |
| Born in Another Country | 0.05 | 348 |
| Missing | 0.00 | 29 |
| Childhood Service Attendance |  |  |
| At Least 1/Week | 0.39 | 2601 |
| 1-3/Month | 0.18 | 1204 |
| <1/Month | 0.16 | 1059 |
| Never | 0.27 | 1808 |
| Missing | 0.01 | 53 |
| Gender |  |  |
| Male | 0.47 | 3143 |
| Female | 0.53 | 3542 |
| Other | 0.00 | 21 |
| Missing | 0.00 | 18 |
| Year of Birth |  |  |
| 1998-2005; Age 18-24 | 0.16 | 1108 |
| 1993-1998; Age 25-29 | 0.11 | 719 |
| 1983-1993; Age 30-39 | 0.21 | 1432 |
| 1973-1983; Age 40-49 | 0.19 | 1254 |
| 1963-1973; Age 50-59 | 0.15 | 1014 |
| 1953-1963; Age 60-69 | 0.11 | 730 |
| 1943-1953; Age 70-79 | 0.05 | 356 |
| 1943 or Earlier; 80 or Older | 0.02 | 112 |
| Missing | . | . |
| Childhood Religion |  |  |
| Christianity | 0.86 | 5805 |
| Islam | 0.00 | 11 |
| Hinduism | 0.00 | 2 |
| Buddhism | 0.00 | 3 |
| Judaism | 0.01 | 51 |
| Sikhism | 0.00 | 5 |
| Baha'i | . | . |
| Jainism | . | . |
| Shinto | . | . |
| Taoism | 0.00 | 1 |
| Confucianism | . | . |
| Primal, Animist, or Folk Religion | 0.00 | 17 |
| Spiritism | . | . |
| African-Derived | . | . |
| Chinese | . | . |
| Some Other Religion | 0.00 | 10 |
| No Religion/Atheist/Agnostic | 0.10 | 697 |
| Missing | 0.02 | 122 |
| Race/Ethnicity |  |  |
| Asian | 0.01 | 43 |
| Black | 0.01 | 95 |
| Indigenous | 0.02 | 129 |
| Mestizo(a) | 0.27 | 1801 |
| Mullato(a) | 0.01 | 75 |
| White | 0.51 | 3406 |
| Other | 0.02 | 104 |
| Missing | 0.16 | 1070 |

**Table S1b: Variations Across Childhood Predictors (Argentina)**

| Variable | IRR | SE | Prob | LCI | UCI | Global p-value |
| --- | --- | --- | --- | --- | --- | --- |
| Relationship with Mother (Ref: Very/Somewhat Bad) |  |  |  |  |  |  |
| Very/Somewhat Good | 0.99 | 0.08 | 0.90 | 0.85 | 1.16 | 0.90 |
| Relationship with Father (Ref: Very/Somewhat Bad) |  |  |  |  |  |  |
| Very/Somewhat Good | 1.04 | 0.07 | 0.61 | 0.90 | 1.19 | 0.61 |
| Parent Marital Status (Ref: Married) |  |  |  |  |  |  |
| Divorced | 1.03 | 0.08 | 0.65 | 0.89 | 1.20 | 0.55 |
| Never Married | 1.04 | 0.07 | 0.59 | 0.91 | 1.17 | . |
| One or Both Had Died | 0.86 | 0.11 | 0.25 | 0.66 | 1.11 | . |
| Childhood Income (Ref: Got By) |  |  |  |  |  |  |
| Lived Comfortably | 1.03 | 0.06 | 0.59 | 0.93 | 1.15 | 0.21 |
| Found it Difficult | 1.06 | 0.06 | 0.31 | 0.95 | 1.18 | . |
| Found it Very Difficult | 1.18 | 0.09 | 0.04 | 1.01 | 1.37 | . |
| Childhood Abuse (Ref: No) |  |  |  |  |  |  |
| Yes | 1.03 | 0.05 | 0.55 | 0.93 | 1.14 | 0.55 |
| Outsider (Ref: No) |  |  |  |  |  |  |
| Yes | 1.09 | 0.07 | 0.17 | 0.96 | 1.23 | 0.17 |
| Childhood Health (Ref: Good) |  |  |  |  |  |  |
| Excellent | 1.00 | 0.06 | 0.96 | 0.89 | 1.11 | 0.10 |
| Very Good | 1.01 | 0.06 | 0.92 | 0.90 | 1.13 | . |
| Fair | 1.05 | 0.08 | 0.53 | 0.90 | 1.23 | . |
| Poor | 1.35 | 0.15 | 0.01 | 1.08 | 1.69 | . |
| Immigration Status (Ref: Born in This Country) |  |  |  |  |  |  |
| Born in Another Country | 1.18 | 0.10 | 0.05 | 1.00 | 1.40 | 0.05 |
| Childhood Service Attendance (Ref: Never) |  |  |  |  |  |  |
| At Least 1/Week | 1.74 | 0.12 | 0.00 | 1.52 | 1.98 | 0.00 |
| 1-3/Month | 1.30 | 0.11 | 0.00 | 1.11 | 1.52 | . |
| <1/Month | 1.23 | 0.11 | 0.02 | 1.04 | 1.45 | . |
| Gender (Ref: Male) |  |  |  |  |  |  |
| Female | 1.30 | 0.06 | 0.00 | 1.19 | 1.42 | 0.00 |
| Other | 1.31 | 0.72 | 0.63 | 0.44 | 3.86 | . |
| Year of Birth (Ref: 1998-2005) |  |  |  |  |  |  |
| 1993-1998; Age 25-29 | 1.27 | 0.15 | 0.04 | 1.01 | 1.59 | 0.00 |
| 1983-1993; Age 30-39 | 1.32 | 0.13 | 0.00 | 1.09 | 1.60 | . |
| 1973-1983; Age 40-49 | 1.46 | 0.15 | 0.00 | 1.20 | 1.78 | . |
| 1963-1973; Age 50-59 | 1.92 | 0.19 | 0.00 | 1.59 | 2.32 | . |
| 1953-1963; Age 60-69 | 1.93 | 0.20 | 0.00 | 1.58 | 2.37 | . |
| 1943-1953; Age 70-79 | 2.51 | 0.26 | 0.00 | 2.04 | 3.09 | . |
| 1943 or Earlier; Age 80 or Older | 2.23 | 0.32 | 0.00 | 1.69 | 2.94 | . |
| Mother Absence/Presence (Ref: Present) |  |  |  |  |  |  |
| Absent | 1.14 | 0.10 | 0.14 | 0.96 | 1.35 | 0.14 |
| Father Absence/Presence (Ref: Present) |  |  |  |  |  |  |
| Absent | 1.11 | 0.09 | 0.18 | 0.95 | 1.31 | 0.18 |
| Childhood Religion (Ref: No Religion/Atheist/Agnostic) |  |  |  |  |  |  |
| Christianity | 1.69 | 0.21 | 0.00 | 1.32 | 2.17 | 0.00 |
| Some Other Religion | 1.51 | 0.37 | 0.09 | 0.93 | 2.45 | . |
| Race/Ethnicity (Ref: Ethnic Plurality) |  |  |  |  |  |  |
| Ethnic Minority | 1.12 | 0.05 | 0.01 | 1.03 | 1.23 | 0.01 |

**Table S1c: E-Values and E-Value Limits (Argentina)**

| Variable | E-Value | E-Value Limit |
| --- | --- | --- |
| Relationship with Mother (Ref: Very/Somewhat Bad) |  |  |
| Very/Somewhat Good | 1.10 | 1.00 |
| Relationship with Father (Ref: Very/Somewhat Bad) |  |  |
| Very/Somewhat Good | 1.21 | 1.00 |
| Parent Marital Status (Ref: Married) |  |  |
| Divorced | 1.20 | 1.00 |
| Never Married | 1.20 | 1.00 |
| One or Both Had Died | 1.52 | 1.00 |
| Childhood Income (Ref: Got By) |  |  |
| Lived Comfortably | 1.18 | 1.00 |
| Found it Difficult | 1.27 | 1.00 |
| Found it Very Difficult | 1.54 | 1.07 |
| Childhood Abuse (Ref: No) |  |  |
| Yes | 1.19 | 1.00 |
| Outsider (Ref: No) |  |  |
| Yes | 1.35 | 1.00 |
| Childhood Health (Ref: Good) |  |  |
| Excellent | 1.05 | 1.00 |
| Very Good | 1.07 | 1.00 |
| Fair | 1.25 | 1.00 |
| Poor | 1.88 | 1.25 |
| Immigration Status (Ref: Born in This Country) |  |  |
| Born in Another Country | 1.56 | 1.00 |
| Childhood Service Attendance (Ref: Never) |  |  |
| At Least 1/Week | 2.51 | 1.77 |
| 1-3/Month | 1.78 | 1.29 |
| <1/Month | 1.65 | 1.16 |
| Gender (Ref: Male) |  |  |
| Female | 1.78 | 1.40 |
| Other | 1.80 | 1.00 |
| Year of Birth (Ref: 1998-2005) |  |  |
| 1993-1998; Age 25-29 | 1.73 | 1.09 |
| 1983-1993; Age 30-39 | 1.83 | 1.26 |
| 1973-1983; Age 40-49 | 2.06 | 1.42 |
| 1963-1973; Age 50-59 | 2.81 | 1.84 |
| 1953-1963; Age 60-69 | 2.82 | 1.82 |
| 1943-1953; Age 70-79 | 3.67 | 2.21 |
| 1943 or Earlier; Age 80 or Older | 3.26 | 1.92 |
| Mother Absence/Presence (Ref: Present) |  |  |
| Absent | 1.46 | 1.00 |
| Father Absence/Presence (Ref: Present) |  |  |
| Absent | 1.41 | 1.00 |
| Childhood Religion (Ref: No Religion/Atheist/Agnostic) |  |  |
| Christianity | 2.45 | 1.56 |
| Some Other Religion | 2.15 | 1.00 |
| Race/Ethnicity (Ref: Ethnic Plurality) |  |  |
| Ethnic Minority | 1.43 | 1.13 |

**Table S2a: Nationally-Representative Descriptive Statistics of the Observed Sample (Australia)**

| Variable | Proportion | Frequency |
| --- | --- | --- |
| Relationship with Mother |  |  |
| Very Good | 0.66 | 2554 |
| Somewhat Good | 0.24 | 925 |
| Somewhat Bad | 0.06 | 218 |
| Very Bad | 0.03 | 107 |
| Not Applicable | 0.01 | 32 |
| Missing | 0.00 | 7 |
| Relationship with Father |  |  |
| Very Good | 0.53 | 2032 |
| Somewhat Good | 0.30 | 1144 |
| Somewhat Bad | 0.08 | 315 |
| Very Bad | 0.05 | 196 |
| Not Applicable | 0.04 | 148 |
| Missing | 0.00 | 9 |
| Parent Marital Status |  |  |
| Married | 0.79 | 3048 |
| Divorced | 0.12 | 462 |
| Never Married | 0.05 | 187 |
| One or Both Had Died | 0.02 | 96 |
| Missing | 0.01 | 52 |
| Childhood Income |  |  |
| Lived Comfortably | 0.46 | 1756 |
| Got By | 0.39 | 1496 |
| Found it Difficult | 0.11 | 422 |
| Found it Very Difficult | 0.04 | 154 |
| Missing | 0.00 | 16 |
| Childhood Abuse |  |  |
| Yes | 0.26 | 995 |
| No | 0.73 | 2790 |
| Missing | 0.02 | 59 |
| Outsider |  |  |
| Yes | 0.20 | 756 |
| No | 0.80 | 3062 |
| Not Applicable | 0.00 | 6 |
| Missing | 0.00 | 19 |
| Childhood Health |  |  |
| Excellent | 0.45 | 1736 |
| Very Good | 0.28 | 1087 |
| Good | 0.16 | 603 |
| Fair | 0.08 | 308 |
| Poor | 0.03 | 106 |
| Missing | 0.00 | 4 |
| Immigration Status |  |  |
| Born in This Country | 0.77 | 2953 |
| Born in Another Country | 0.23 | 885 |
| Missing | 0.00 | 6 |
| Childhood Service Attendance |  |  |
| At Least 1/Week | 0.35 | 1362 |
| 1-3/Month | 0.13 | 486 |
| <1/Month | 0.16 | 600 |
| Never | 0.34 | 1307 |
| Missing | 0.02 | 90 |
| Gender |  |  |
| Male | 0.48 | 1861 |
| Female | 0.50 | 1941 |
| Other | 0.01 | 36 |
| Missing | 0.00 | 6 |
| Year of Birth |  |  |
| 1998-2005; Age 18-24 | 0.09 | 345 |
| 1993-1998; Age 25-29 | 0.07 | 282 |
| 1983-1993; Age 30-39 | 0.17 | 641 |
| 1973-1983; Age 40-49 | 0.16 | 618 |
| 1963-1973; Age 50-59 | 0.18 | 691 |
| 1953-1963; Age 60-69 | 0.15 | 589 |
| 1943-1953; Age 70-79 | 0.13 | 498 |
| 1943 or Earlier; 80 or Older | 0.05 | 178 |
| Missing | 0.00 | 2 |
| Childhood Religion |  |  |
| Christianity | 0.70 | 2678 |
| Islam | 0.01 | 48 |
| Hinduism | 0.01 | 39 |
| Buddhism | 0.00 | 16 |
| Judaism | 0.01 | 29 |
| Sikhism | 0.00 | 6 |
| Baha'i | 0.00 | 5 |
| Jainism | . | . |
| Shinto | . | . |
| Taoism | 0.00 | 1 |
| Confucianism | . | . |
| Primal, Animist, or Folk Religion | 0.00 | 4 |
| Spiritism | . | . |
| African-Derived | . | . |
| Chinese | . | . |
| Some Other Religion | 0.00 | 8 |
| No Religion/Atheist/Agnostic | 0.26 | 990 |
| Missing | 0.01 | 21 |
| Race/Ethnicity |  |  |
| Aboriginal | 0.01 | 53 |
| Australian | 0.51 | 1946 |
| Australian /British/European | 0.27 | 1047 |
| Chinese | 0.02 | 75 |
| Indian | 0.02 | 58 |
| Japanese | 0.00 | 1 |
| Malay | 0.00 | 11 |
| Sinhalese | 0.00 | 1 |
| Spanish | 0.00 | 2 |
| Sri Lankan Moor | 0.00 | 1 |
| Sri Lankan Tamil | 0.00 | 7 |
| Vietnamese | 0.00 | 7 |
| Taiwanese/Holo | . | . |
| Russian | 0.00 | 7 |
| Samoan | 0.00 | 4 |
| New Zealander | 0.02 | 91 |
| Other European | 0.09 | 357 |
| Other | 0.04 | 163 |
| Missing | 0.00 | 14 |

**Table S2b: Variations Across Childhood Predictors (Australia)**

| Variable | IRR | SE | Prob | LCI | UCI | Global p-value |
| --- | --- | --- | --- | --- | --- | --- |
| Relationship with Mother (Ref: Very/Somewhat Bad) |  |  |  |  |  |  |
| Very/Somewhat Good | 1.13 | 0.17 | 0.43 | 0.84 | 1.53 | 0.43 |
| Relationship with Father (Ref: Very/Somewhat Bad) |  |  |  |  |  |  |
| Very/Somewhat Good | 1.14 | 0.15 | 0.32 | 0.88 | 1.46 | 0.32 |
| Parent Marital Status (Ref: Married) |  |  |  |  |  |  |
| Divorced | 0.93 | 0.14 | 0.64 | 0.69 | 1.26 | 0.57 |
| Never Married | 1.31 | 0.32 | 0.26 | 0.82 | 2.11 | . |
| One or Both Had Died | 0.87 | 0.23 | 0.60 | 0.53 | 1.45 | . |
| Childhood Income (Ref: Got By) |  |  |  |  |  |  |
| Lived Comfortably | 0.92 | 0.07 | 0.28 | 0.78 | 1.07 | 0.59 |
| Found it Difficult | 0.86 | 0.11 | 0.26 | 0.66 | 1.12 | . |
| Found it Very Difficult | 0.97 | 0.21 | 0.88 | 0.64 | 1.47 | . |
| Childhood Abuse (Ref: No) |  |  |  |  |  |  |
| Yes | 1.37 | 0.11 | 0.00 | 1.17 | 1.62 | 0.00 |
| Outsider (Ref: No) |  |  |  |  |  |  |
| Yes | 1.13 | 0.12 | 0.25 | 0.92 | 1.39 | 0.25 |
| Childhood Health (Ref: Good) |  |  |  |  |  |  |
| Excellent | 1.05 | 0.12 | 0.67 | 0.84 | 1.30 | 0.87 |
| Very Good | 0.98 | 0.12 | 0.90 | 0.78 | 1.24 | . |
| Fair | 1.14 | 0.19 | 0.43 | 0.83 | 1.58 | . |
| Poor | 1.05 | 0.29 | 0.86 | 0.61 | 1.82 | . |
| Immigration Status (Ref: Born in This Country) |  |  |  |  |  |  |
| Born in Another Country | 1.17 | 0.11 | 0.08 | 0.98 | 1.40 | 0.08 |
| Childhood Service Attendance (Ref: Never) |  |  |  |  |  |  |
| At Least 1/Week | 2.31 | 0.28 | 0.00 | 1.82 | 2.92 | 0.00 |
| 1-3/Month | 1.52 | 0.23 | 0.01 | 1.13 | 2.05 | . |
| <1/Month | 1.16 | 0.18 | 0.36 | 0.85 | 1.58 | . |
| Gender (Ref: Male) |  |  |  |  |  |  |
| Female | 1.45 | 0.11 | 0.00 | 1.25 | 1.69 | 0.00 |
| Other | 0.95 | 0.61 | 0.93 | 0.27 | 3.36 | . |
| Year of Birth (Ref: 1998-2005) |  |  |  |  |  |  |
| 1993-1998; Age 25-29 | 1.20 | 0.29 | 0.45 | 0.75 | 1.94 | 0.00 |
| 1983-1993; Age 30-39 | 1.02 | 0.22 | 0.93 | 0.67 | 1.54 | . |
| 1973-1983; Age 40-49 | 1.29 | 0.26 | 0.20 | 0.87 | 1.92 | . |
| 1963-1973; Age 50-59 | 1.09 | 0.22 | 0.66 | 0.74 | 1.62 | . |
| 1953-1963; Age 60-69 | 1.05 | 0.21 | 0.80 | 0.71 | 1.55 | . |
| 1943-1953; Age 70-79 | 1.27 | 0.26 | 0.24 | 0.85 | 1.88 | . |
| 1943 or Earlier; Age 80 or Older | 1.82 | 0.38 | 0.00 | 1.21 | 2.74 | . |
| Mother Absence/Presence (Ref: Present) |  |  |  |  |  |  |
| Absent | 0.43 | 0.14 | 0.01 | 0.23 | 0.83 | 0.01 |
| Father Absence/Presence (Ref: Present) |  |  |  |  |  |  |
| Absent | 1.39 | 0.29 | 0.11 | 0.92 | 2.10 | 0.11 |
| Childhood Religion (Ref: No Religion/Atheist/Agnostic) |  |  |  |  |  |  |
| Christianity | 2.05 | 0.32 | 0.00 | 1.50 | 2.79 | 0.00 |
| Some Other Religion | 3.72 | 0.73 | 0.00 | 2.53 | 5.47 | . |
| Race/Ethnicity (Ref: Ethnic Plurality) |  |  |  |  |  |  |
| Ethnic Minority | 1.06 | 0.09 | 0.48 | 0.90 | 1.25 | 0.48 |

**Table S2c: E-Values and E-Value Limits (Australia)**

| Variable | E-Value | E-Value Limit |
| --- | --- | --- |
| Relationship with Mother (Ref: Very/Somewhat Bad) |  |  |
| Very/Somewhat Good | 1.55 | 1.00 |
| Relationship with Father (Ref: Very/Somewhat Bad) |  |  |
| Very/Somewhat Good | 1.56 | 1.00 |
| Parent Marital Status (Ref: Married) |  |  |
| Divorced | 1.38 | 1.00 |
| Never Married | 2.03 | 1.00 |
| One or Both Had Died | 1.58 | 1.00 |
| Childhood Income (Ref: Got By) |  |  |
| Lived Comfortably | 1.42 | 1.00 |
| Found it Difficult | 1.63 | 1.00 |
| Found it Very Difficult | 1.23 | 1.00 |
| Childhood Abuse (Ref: No) |  |  |
| Yes | 2.17 | 1.37 |
| Outsider (Ref: No) |  |  |
| Yes | 1.54 | 1.00 |
| Childhood Health (Ref: Good) |  |  |
| Excellent | 1.28 | 1.00 |
| Very Good | 1.15 | 1.00 |
| Fair | 1.57 | 1.00 |
| Poor | 1.30 | 1.00 |
| Immigration Status (Ref: Born in This Country) |  |  |
| Born in Another Country | 1.66 | 1.00 |
| Childhood Service Attendance (Ref: Never) |  |  |
| At Least 1/Week | 4.38 | 2.04 |
| 1-3/Month | 2.53 | 1.33 |
| <1/Month | 1.61 | 1.00 |
| Gender (Ref: Male) |  |  |
| Female | 2.37 | 1.48 |
| Other | 1.31 | 1.00 |
| Year of Birth (Ref: 1998-2005) |  |  |
| 1993-1998; Age 25-29 | 1.74 | 1.00 |
| 1983-1993; Age 30-39 | 1.17 | 1.00 |
| 1973-1983; Age 40-49 | 1.97 | 1.00 |
| 1963-1973; Age 50-59 | 1.43 | 1.00 |
| 1953-1963; Age 60-69 | 1.30 | 1.00 |
| 1943-1953; Age 70-79 | 1.90 | 1.00 |
| 1943 or Earlier; Age 80 or Older | 3.23 | 1.43 |
| Mother Absence/Presence (Ref: Present) |  |  |
| Absent | 4.39 | 1.43 |
| Father Absence/Presence (Ref: Present) |  |  |
| Absent | 2.22 | 1.00 |
| Childhood Religion (Ref: No Religion/Atheist/Agnostic) |  |  |
| Christianity | 3.77 | 1.75 |
| Some Other Religion | 7.77 | 2.56 |
| Race/Ethnicity (Ref: Ethnic Plurality) |  |  |
| Ethnic Minority | 1.33 | 1.00 |

**Table S3a: Nationally-Representative Descriptive Statistics of the Observed Sample (Brazil)**

| Variable | Proportion | Frequency |
| --- | --- | --- |
| Relationship with Mother |  |  |
| Very Good | 0.63 | 8369 |
| Somewhat Good | 0.27 | 3559 |
| Somewhat Bad | 0.04 | 483 |
| Very Bad | 0.02 | 214 |
| Not Applicable | 0.04 | 507 |
| Missing | 0.01 | 73 |
| Relationship with Father |  |  |
| Very Good | 0.48 | 6364 |
| Somewhat Good | 0.28 | 3654 |
| Somewhat Bad | 0.08 | 1035 |
| Very Bad | 0.06 | 756 |
| Not Applicable | 0.10 | 1303 |
| Missing | 0.01 | 93 |
| Parent Marital Status |  |  |
| Married | 0.65 | 8546 |
| Divorced | 0.10 | 1384 |
| Never Married | 0.15 | 1985 |
| One or Both Had Died | 0.04 | 508 |
| Missing | 0.06 | 781 |
| Childhood Income |  |  |
| Lived Comfortably | 0.38 | 4998 |
| Got By | 0.35 | 4616 |
| Found it Difficult | 0.19 | 2484 |
| Found it Very Difficult | 0.08 | 1027 |
| Missing | 0.01 | 79 |
| Childhood Abuse |  |  |
| Yes | 0.20 | 2606 |
| No | 0.77 | 10147 |
| Missing | 0.03 | 451 |
| Outsider |  |  |
| Yes | 0.13 | 1659 |
| No | 0.85 | 11234 |
| Not Applicable | 0.02 | 229 |
| Missing | 0.01 | 82 |
| Childhood Health |  |  |
| Excellent | 0.40 | 5312 |
| Very Good | 0.26 | 3392 |
| Good | 0.22 | 2873 |
| Fair | 0.10 | 1368 |
| Poor | 0.02 | 228 |
| Missing | 0.00 | 30 |
| Immigration Status |  |  |
| Born in This Country | 0.96 | 12688 |
| Born in Another Country | 0.01 | 153 |
| Missing | 0.03 | 363 |
| Childhood Service Attendance |  |  |
| At Least 1/Week | 0.48 | 6306 |
| 1-3/Month | 0.19 | 2491 |
| <1/Month | 0.20 | 2629 |
| Never | 0.13 | 1707 |
| Missing | 0.01 | 71 |
| Gender |  |  |
| Male | 0.48 | 6320 |
| Female | 0.52 | 6820 |
| Other | 0.00 | 35 |
| Missing | 0.00 | 30 |
| Year of Birth |  |  |
| 1998-2005; Age 18-24 | 0.15 | 1986 |
| 1993-1998; Age 25-29 | 0.11 | 1468 |
| 1983-1993; Age 30-39 | 0.22 | 2908 |
| 1973-1983; Age 40-49 | 0.20 | 2638 |
| 1963-1973; Age 50-59 | 0.16 | 2131 |
| 1953-1963; Age 60-69 | 0.11 | 1435 |
| 1943-1953; Age 70-79 | 0.04 | 510 |
| 1943 or Earlier; 80 or Older | 0.01 | 126 |
| Missing | . | . |
| Childhood Religion |  |  |
| Christianity | 0.86 | 11403 |
| Islam | 0.00 | 14 |
| Hinduism | 0.00 | 0 |
| Buddhism | 0.00 | 27 |
| Judaism | 0.00 | 40 |
| Sikhism | . | . |
| Baha'i | 0.00 | 1 |
| Jainism | 0.00 | 4 |
| Shinto | 0.00 | 4 |
| Taoism | 0.00 | 0 |
| Confucianism | 0.00 | 7 |
| Primal, Animist, or Folk Religion | 0.00 | 17 |
| Spiritism | 0.03 | 336 |
| African-Derived | 0.02 | 262 |
| Chinese | . | . |
| Some Other Religion | 0.01 | 87 |
| No Religion/Atheist/Agnostic | 0.07 | 908 |
| Missing | 0.01 | 94 |
| Race/Ethnicity |  |  |
| Branca | 0.39 | 5169 |
| Preta | 0.12 | 1615 |
| Parda | 0.39 | 5125 |
| Amarela | 0.02 | 238 |
| Indigena | 0.01 | 131 |
| Other | 0.00 | 61 |
| Missing | 0.07 | 865 |

**Table S3b: Variations Across Childhood Predictors (Brazil)**

| Variable | IRR | SE | Prob | LCI | UCI | Global p-value |
| --- | --- | --- | --- | --- | --- | --- |
| Relationship with Mother (Ref: Very/Somewhat Bad) |  |  |  |  |  |  |
| Very/Somewhat Good | 1.05 | 0.04 | 0.16 | 0.98 | 1.13 | 0.16 |
| Relationship with Father (Ref: Very/Somewhat Bad) |  |  |  |  |  |  |
| Very/Somewhat Good | 1.03 | 0.03 | 0.20 | 0.98 | 1.08 | 0.20 |
| Parent Marital Status (Ref: Married) |  |  |  |  |  |  |
| Divorced | 0.98 | 0.03 | 0.58 | 0.92 | 1.05 | 0.74 |
| Never Married | 0.97 | 0.03 | 0.31 | 0.92 | 1.03 | . |
| One or Both Had Died | 0.98 | 0.04 | 0.62 | 0.90 | 1.07 | . |
| Childhood Income (Ref: Got By) |  |  |  |  |  |  |
| Lived Comfortably | 1.00 | 0.02 | 0.87 | 0.96 | 1.04 | 0.46 |
| Found it Difficult | 1.00 | 0.02 | 0.86 | 0.96 | 1.05 | . |
| Found it Very Difficult | 1.05 | 0.03 | 0.14 | 0.99 | 1.11 | . |
| Childhood Abuse (Ref: No) |  |  |  |  |  |  |
| Yes | 0.97 | 0.02 | 0.20 | 0.93 | 1.02 | 0.20 |
| Outsider (Ref: No) |  |  |  |  |  |  |
| Yes | 0.95 | 0.03 | 0.06 | 0.90 | 1.00 | 0.06 |
| Childhood Health (Ref: Good) |  |  |  |  |  |  |
| Excellent | 1.02 | 0.02 | 0.47 | 0.97 | 1.06 | 0.60 |
| Very Good | 0.99 | 0.02 | 0.62 | 0.94 | 1.04 | . |
| Fair | 0.98 | 0.03 | 0.48 | 0.92 | 1.04 | . |
| Poor | 1.00 | 0.06 | 0.97 | 0.89 | 1.11 | . |
| Immigration Status (Ref: Born in This Country) |  |  |  |  |  |  |
| Born in Another Country | 1.02 | 0.09 | 0.84 | 0.85 | 1.21 | 0.84 |
| Childhood Service Attendance (Ref: Never) |  |  |  |  |  |  |
| At Least 1/Week | 1.43 | 0.05 | 0.00 | 1.33 | 1.52 | 0.00 |
| 1-3/Month | 1.28 | 0.05 | 0.00 | 1.19 | 1.38 | . |
| <1/Month | 1.11 | 0.04 | 0.01 | 1.02 | 1.19 | . |
| Gender (Ref: Male) |  |  |  |  |  |  |
| Female | 1.19 | 0.02 | 0.00 | 1.15 | 1.23 | 0.00 |
| Other | 0.98 | 0.13 | 0.87 | 0.75 | 1.27 | . |
| Year of Birth (Ref: 1998-2005) |  |  |  |  |  |  |
| 1993-1998; Age 25-29 | 1.15 | 0.04 | 0.00 | 1.06 | 1.24 | 0.00 |
| 1983-1993; Age 30-39 | 1.28 | 0.04 | 0.00 | 1.20 | 1.36 | . |
| 1973-1983; Age 40-49 | 1.44 | 0.05 | 0.00 | 1.35 | 1.54 | . |
| 1963-1973; Age 50-59 | 1.60 | 0.05 | 0.00 | 1.50 | 1.71 | . |
| 1953-1963; Age 60-69 | 1.66 | 0.06 | 0.00 | 1.54 | 1.78 | . |
| 1943-1953; Age 70-79 | 1.71 | 0.08 | 0.00 | 1.56 | 1.88 | . |
| 1943 or Earlier; Age 80 or Older | 1.68 | 0.14 | 0.00 | 1.42 | 1.99 | . |
| Mother Absence/Presence (Ref: Present) |  |  |  |  |  |  |
| Absent | 1.00 | 0.04 | 0.99 | 0.93 | 1.08 | 0.99 |
| Father Absence/Presence (Ref: Present) |  |  |  |  |  |  |
| Absent | 1.01 | 0.03 | 0.62 | 0.96 | 1.07 | 0.62 |
| Childhood Religion (Ref: No Religion/Atheist/Agnostic) |  |  |  |  |  |  |
| Christianity | 1.20 | 0.05 | 0.00 | 1.10 | 1.31 | 0.00 |
| Some Other Religion | 1.21 | 0.07 | 0.00 | 1.09 | 1.35 | . |
| Race/Ethnicity (Ref: Ethnic Plurality) |  |  |  |  |  |  |
| Ethnic Minority | 1.08 | 0.02 | 0.00 | 1.04 | 1.12 | 0.00 |

**Table S3c: E-Values and E-Value Limits (Brazil)**

| Variable | E-Value | E-Value Limit |
| --- | --- | --- |
| Relationship with Mother (Ref: Very/Somewhat Bad) |  |  |
| Very/Somewhat Good | 1.21 | 1.00 |
| Relationship with Father (Ref: Very/Somewhat Bad) |  |  |
| Very/Somewhat Good | 1.16 | 1.00 |
| Parent Marital Status (Ref: Married) |  |  |
| Divorced | 1.12 | 1.00 |
| Never Married | 1.16 | 1.00 |
| One or Both Had Died | 1.13 | 1.00 |
| Childhood Income (Ref: Got By) |  |  |
| Lived Comfortably | 1.05 | 1.00 |
| Found it Difficult | 1.05 | 1.00 |
| Found it Very Difficult | 1.20 | 1.00 |
| Childhood Abuse (Ref: No) |  |  |
| Yes | 1.15 | 1.00 |
| Outsider (Ref: No) |  |  |
| Yes | 1.21 | 1.00 |
| Childhood Health (Ref: Good) |  |  |
| Excellent | 1.11 | 1.00 |
| Very Good | 1.09 | 1.00 |
| Fair | 1.13 | 1.00 |
| Poor | 1.04 | 1.00 |
| Immigration Status (Ref: Born in This Country) |  |  |
| Born in Another Country | 1.12 | 1.00 |
| Childhood Service Attendance (Ref: Never) |  |  |
| At Least 1/Week | 1.80 | 1.58 |
| 1-3/Month | 1.61 | 1.41 |
| <1/Month | 1.33 | 1.12 |
| Gender (Ref: Male) |  |  |
| Female | 1.47 | 1.35 |
| Other | 1.13 | 1.00 |
| Year of Birth (Ref: 1998-2005) |  |  |
| 1993-1998; Age 25-29 | 1.40 | 1.21 |
| 1983-1993; Age 30-39 | 1.60 | 1.41 |
| 1973-1983; Age 40-49 | 1.82 | 1.60 |
| 1963-1973; Age 50-59 | 2.02 | 1.75 |
| 1953-1963; Age 60-69 | 2.08 | 1.79 |
| 1943-1953; Age 70-79 | 2.14 | 1.80 |
| 1943 or Earlier; Age 80 or Older | 2.11 | 1.67 |
| Mother Absence/Presence (Ref: Present) |  |  |
| Absent | 1.02 | 1.00 |
| Father Absence/Presence (Ref: Present) |  |  |
| Absent | 1.10 | 1.00 |
| Childhood Religion (Ref: No Religion/Atheist/Agnostic) |  |  |
| Christianity | 1.48 | 1.27 |
| Some Other Religion | 1.50 | 1.25 |
| Race/Ethnicity (Ref: Ethnic Plurality) |  |  |
| Ethnic Minority | 1.27 | 1.16 |

**Table S4a: Nationally-Representative Descriptive Statistics of the Observed Sample (Egypt)**

| Variable | Proportion | Frequency |
| --- | --- | --- |
| Relationship with Mother |  |  |
| Very Good | 0.87 | 4110 |
| Somewhat Good | 0.11 | 505 |
| Somewhat Bad | 0.00 | 21 |
| Very Bad | 0.00 | 10 |
| Not Applicable | 0.02 | 83 |
| Missing | . | . |
| Relationship with Father |  |  |
| Very Good | 0.79 | 3713 |
| Somewhat Good | 0.14 | 683 |
| Somewhat Bad | 0.01 | 56 |
| Very Bad | 0.01 | 30 |
| Not Applicable | 0.05 | 233 |
| Missing | 0.00 | 14 |
| Parent Marital Status |  |  |
| Married | 0.86 | 4049 |
| Divorced | 0.03 | 131 |
| Never Married | 0.00 | 9 |
| One or Both Had Died | 0.10 | 485 |
| Missing | 0.01 | 55 |
| Childhood Income |  |  |
| Lived Comfortably | 0.26 | 1251 |
| Got By | 0.50 | 2352 |
| Found it Difficult | 0.18 | 857 |
| Found it Very Difficult | 0.06 | 268 |
| Missing | 0.00 | 1 |
| Childhood Abuse |  |  |
| Yes | 0.09 | 405 |
| No | 0.91 | 4293 |
| Missing | 0.01 | 30 |
| Outsider |  |  |
| Yes | 0.05 | 260 |
| No | 0.94 | 4456 |
| Not Applicable | 0.00 | 4 |
| Missing | 0.00 | 10 |
| Childhood Health |  |  |
| Excellent | 0.57 | 2687 |
| Very Good | 0.25 | 1174 |
| Good | 0.11 | 497 |
| Fair | 0.06 | 265 |
| Poor | 0.02 | 106 |
| Missing | 0.00 | 1 |
| Immigration Status |  |  |
| Born in This Country | 1.00 | 4713 |
| Born in Another Country | 0.00 | 16 |
| Missing | 0.00 | 1 |
| Childhood Service Attendance |  |  |
| At Least 1/Week | 0.49 | 2307 |
| 1-3/Month | 0.12 | 570 |
| <1/Month | 0.13 | 629 |
| Never | 0.25 | 1165 |
| Missing | 0.01 | 57 |
| Gender |  |  |
| Male | 0.51 | 2394 |
| Female | 0.49 | 2334 |
| Other | . | . |
| Missing | 0.00 | 0 |
| Year of Birth |  |  |
| 1998-2005; Age 18-24 | 0.20 | 960 |
| 1993-1998; Age 25-29 | 0.13 | 607 |
| 1983-1993; Age 30-39 | 0.25 | 1204 |
| 1973-1983; Age 40-49 | 0.19 | 897 |
| 1963-1973; Age 50-59 | 0.13 | 613 |
| 1953-1963; Age 60-69 | 0.08 | 387 |
| 1943-1953; Age 70-79 | 0.01 | 54 |
| 1943 or Earlier; 80 or Older | 0.00 | 7 |
| Missing | . | . |
| Childhood Religion |  |  |
| Christianity | 0.03 | 123 |
| Islam | 0.97 | 4602 |
| Hinduism | . | . |
| Buddhism | . | . |
| Judaism | . | . |
| Sikhism | . | . |
| Baha'i | . | . |
| Jainism | 0.00 | 1 |
| Shinto | . | . |
| Taoism | 0.00 | 0 |
| Confucianism | . | . |
| Primal, Animist, or Folk Religion | . | . |
| Spiritism | . | . |
| African-Derived | . | . |
| Chinese | . | . |
| Some Other Religion | . | . |
| No Religion/Atheist/Agnostic | . | . |
| Missing | 0.00 | 3 |
| Race/Ethnicity |  |  |
| Arab | 0.97 | 4585 |
| Turkish | 0.00 | 9 |
| Greek | 0.00 | 1 |
| Abazas | . | . |
| Bedouin Arab | 0.00 | 4 |
| Swiss | . | . |
| Nubian | 0.01 | 27 |
| Other | . | . |
| Missing | 0.02 | 102 |

**Table S4b: Variations Across Childhood Predictors (Egypt)**

| Variable | IRR | SE | Prob | LCI | UCI | Global p-value |
| --- | --- | --- | --- | --- | --- | --- |
| Relationship with Mother (Ref: Very/Somewhat Bad) |  |  |  |  |  |  |
| Very/Somewhat Good | 1.18 | 0.11 | 0.09 | 0.98 | 1.41 | 0.09 |
| Relationship with Father (Ref: Very/Somewhat Bad) |  |  |  |  |  |  |
| Very/Somewhat Good | 0.98 | 0.04 | 0.65 | 0.90 | 1.07 | 0.65 |
| Parent Marital Status (Ref: Married) |  |  |  |  |  |  |
| Divorced | 0.87 | 0.07 | 0.06 | 0.75 | 1.01 | 0.02 |
| Never Married | 1.19 | 0.09 | 0.03 | 1.02 | 1.38 | . |
| One or Both Had Died | 0.97 | 0.03 | 0.35 | 0.91 | 1.03 | . |
| Childhood Income (Ref: Got By) |  |  |  |  |  |  |
| Lived Comfortably | 1.02 | 0.02 | 0.47 | 0.97 | 1.07 | 0.74 |
| Found it Difficult | 0.98 | 0.02 | 0.46 | 0.94 | 1.03 | . |
| Found it Very Difficult | 1.01 | 0.04 | 0.79 | 0.94 | 1.09 | . |
| Childhood Abuse (Ref: No) |  |  |  |  |  |  |
| Yes | 0.93 | 0.04 | 0.07 | 0.85 | 1.01 | 0.07 |
| Outsider (Ref: No) |  |  |  |  |  |  |
| Yes | 0.92 | 0.04 | 0.08 | 0.85 | 1.01 | 0.08 |
| Childhood Health (Ref: Good) |  |  |  |  |  |  |
| Excellent | 0.99 | 0.03 | 0.64 | 0.93 | 1.04 | 0.07 |
| Very Good | 0.93 | 0.03 | 0.03 | 0.88 | 0.99 | . |
| Fair | 1.00 | 0.04 | 0.99 | 0.92 | 1.09 | . |
| Poor | 0.93 | 0.07 | 0.36 | 0.80 | 1.09 | . |
| Immigration Status (Ref: Born in This Country) |  |  |  |  |  |  |
| Born in Another Country | 1.01 | 0.15 | 0.95 | 0.76 | 1.35 | 0.95 |
| Childhood Service Attendance (Ref: Never) |  |  |  |  |  |  |
| At Least 1/Week | 1.12 | 0.03 | 0.00 | 1.07 | 1.17 | 0.00 |
| 1-3/Month | 1.06 | 0.03 | 0.05 | 1.00 | 1.13 | . |
| <1/Month | 1.04 | 0.03 | 0.14 | 0.99 | 1.09 | . |
| Gender (Ref: Male) |  |  |  |  |  |  |
| Female | 1.16 | 0.02 | 0.00 | 1.12 | 1.20 | 0.00 |
| Other | 1.00 | . | . | . | . | . |
| Year of Birth (Ref: 1998-2005) |  |  |  |  |  |  |
| 1993-1998; Age 25-29 | 0.97 | 0.04 | 0.50 | 0.89 | 1.06 | 0.00 |
| 1983-1993; Age 30-39 | 1.04 | 0.03 | 0.18 | 0.98 | 1.11 | . |
| 1973-1983; Age 40-49 | 1.09 | 0.04 | 0.01 | 1.02 | 1.17 | . |
| 1963-1973; Age 50-59 | 1.22 | 0.04 | 0.00 | 1.15 | 1.30 | . |
| 1953-1963; Age 60-69 | 1.28 | 0.04 | 0.00 | 1.20 | 1.37 | . |
| 1943-1953; Age 70-79 | 1.21 | 0.08 | 0.00 | 1.07 | 1.37 | . |
| 1943 or Earlier; Age 80 or Older | 1.35 | 0.05 | 0.00 | 1.26 | 1.45 | . |
| Mother Absence/Presence (Ref: Present) |  |  |  |  |  |  |
| Absent | 1.09 | 0.10 | 0.31 | 0.92 | 1.30 | 0.31 |
| Father Absence/Presence (Ref: Present) |  |  |  |  |  |  |
| Absent | 0.97 | 0.05 | 0.54 | 0.87 | 1.08 | 0.54 |
| Childhood Religion (Ref: Islam) |  |  |  |  |  |  |
| Some Other Religion | 0.95 | 0.05 | 0.38 | 0.85 | 1.07 | 0.38 |
| Race/Ethnicity (Ref: Ethnic Plurality) |  |  |  |  |  |  |
| Ethnic Minority | 1.09 | 0.08 | 0.23 | 0.95 | 1.26 | 0.23 |

**Table S4c: E-Values and E-Value Limits (Egypt)**

| Variable | E-Value | E-Value Limit |
| --- | --- | --- |
| Relationship with Mother (Ref: Very/Somewhat Bad) |  |  |
| Very/Somewhat Good | 1.42 | 1.00 |
| Relationship with Father (Ref: Very/Somewhat Bad) |  |  |
| Very/Somewhat Good | 1.12 | 1.00 |
| Parent Marital Status (Ref: Married) |  |  |
| Divorced | 1.39 | 1.00 |
| Never Married | 1.44 | 1.11 |
| One or Both Had Died | 1.15 | 1.00 |
| Childhood Income (Ref: Got By) |  |  |
| Lived Comfortably | 1.11 | 1.00 |
| Found it Difficult | 1.11 | 1.00 |
| Found it Very Difficult | 1.08 | 1.00 |
| Childhood Abuse (Ref: No) |  |  |
| Yes | 1.26 | 1.00 |
| Outsider (Ref: No) |  |  |
| Yes | 1.26 | 1.00 |
| Childhood Health (Ref: Good) |  |  |
| Excellent | 1.09 | 1.00 |
| Very Good | 1.24 | 1.07 |
| Fair | 1.02 | 1.00 |
| Poor | 1.25 | 1.00 |
| Immigration Status (Ref: Born in This Country) |  |  |
| Born in Another Country | 1.07 | 1.00 |
| Childhood Service Attendance (Ref: Never) |  |  |
| At Least 1/Week | 1.33 | 1.21 |
| 1-3/Month | 1.22 | 1.01 |
| <1/Month | 1.17 | 1.00 |
| Gender (Ref: Male) |  |  |
| Female | 1.39 | 1.30 |
| Other | 1.00 | 1.00 |
| Year of Birth (Ref: 1998-2005) |  |  |
| 1993-1998; Age 25-29 | 1.15 | 1.00 |
| 1983-1993; Age 30-39 | 1.18 | 1.00 |
| 1973-1983; Age 40-49 | 1.28 | 1.11 |
| 1963-1973; Age 50-59 | 1.48 | 1.35 |
| 1953-1963; Age 60-69 | 1.57 | 1.42 |
| 1943-1953; Age 70-79 | 1.47 | 1.22 |
| 1943 or Earlier; Age 80 or Older | 1.65 | 1.49 |
| Mother Absence/Presence (Ref: Present) |  |  |
| Absent | 1.28 | 1.00 |
| Father Absence/Presence (Ref: Present) |  |  |
| Absent | 1.16 | 1.00 |
| Childhood Religion (Ref: Islam) |  |  |
| Some Other Religion | 1.20 | 1.00 |
| Race/Ethnicity (Ref: Ethnic Plurality) |  |  |
| Ethnic Minority | 1.28 | 1.00 |

**Table S5a: Nationally-Representative Descriptive Statistics of the Observed Sample (Germany)**

| Variable | Proportion | Frequency |
| --- | --- | --- |
| Relationship with Mother |  |  |
| Very Good | 0.58 | 5497 |
| Somewhat Good | 0.32 | 3031 |
| Somewhat Bad | 0.05 | 496 |
| Very Bad | 0.02 | 187 |
| Not Applicable | 0.03 | 241 |
| Missing | 0.01 | 54 |
| Relationship with Father |  |  |
| Very Good | 0.49 | 4652 |
| Somewhat Good | 0.32 | 3012 |
| Somewhat Bad | 0.09 | 846 |
| Very Bad | 0.04 | 385 |
| Not Applicable | 0.06 | 538 |
| Missing | 0.01 | 73 |
| Parent Marital Status |  |  |
| Married | 0.80 | 7620 |
| Divorced | 0.10 | 927 |
| Never Married | 0.06 | 578 |
| One or Both Had Died | 0.03 | 245 |
| Missing | 0.01 | 136 |
| Childhood Income |  |  |
| Lived Comfortably | 0.33 | 3177 |
| Got By | 0.47 | 4508 |
| Found it Difficult | 0.16 | 1481 |
| Found it Very Difficult | 0.03 | 314 |
| Missing | 0.00 | 26 |
| Childhood Abuse |  |  |
| Yes | 0.11 | 1086 |
| No | 0.88 | 8321 |
| Missing | 0.01 | 99 |
| Outsider |  |  |
| Yes | 0.12 | 1105 |
| No | 0.87 | 8262 |
| Not Applicable | 0.01 | 114 |
| Missing | 0.00 | 25 |
| Childhood Health |  |  |
| Excellent | 0.28 | 2633 |
| Very Good | 0.37 | 3518 |
| Good | 0.27 | 2582 |
| Fair | 0.06 | 612 |
| Poor | 0.01 | 134 |
| Missing | 0.00 | 26 |
| Immigration Status |  |  |
| Born in This Country | 0.92 | 8722 |
| Born in Another Country | 0.08 | 744 |
| Missing | 0.00 | 40 |
| Childhood Service Attendance |  |  |
| At Least 1/Week | 0.20 | 1943 |
| 1-3/Month | 0.20 | 1899 |
| <1/Month | 0.30 | 2887 |
| Never | 0.29 | 2749 |
| Missing | 0.00 | 27 |
| Gender |  |  |
| Male | 0.49 | 4641 |
| Female | 0.51 | 4843 |
| Other | 0.00 | 11 |
| Missing | 0.00 | 11 |
| Year of Birth |  |  |
| 1998-2005; Age 18-24 | 0.09 | 829 |
| 1993-1998; Age 25-29 | 0.08 | 774 |
| 1983-1993; Age 30-39 | 0.15 | 1438 |
| 1973-1983; Age 40-49 | 0.16 | 1494 |
| 1963-1973; Age 50-59 | 0.18 | 1729 |
| 1953-1963; Age 60-69 | 0.20 | 1915 |
| 1943-1953; Age 70-79 | 0.12 | 1137 |
| 1943 or Earlier; 80 or Older | 0.02 | 190 |
| Missing | . | . |
| Childhood Religion |  |  |
| Christianity | 0.61 | 5751 |
| Islam | 0.04 | 350 |
| Hinduism | 0.00 | 15 |
| Buddhism | 0.00 | 25 |
| Judaism | 0.00 | 18 |
| Sikhism | 0.00 | 5 |
| Baha'i | 0.00 | 2 |
| Jainism | 0.00 | 1 |
| Shinto | . | . |
| Taoism | . | . |
| Confucianism | 0.00 | 4 |
| Primal, Animist, or Folk Religion | 0.00 | 19 |
| Spiritism | . | . |
| African-Derived | . | . |
| Chinese | . | . |
| Some Other Religion | 0.01 | 67 |
| No Religion/Atheist/Agnostic | 0.33 | 3163 |
| Missing | 0.01 | 85 |
| Race/Ethnicity |  |  |
| No Data | . | . |

**Table S5b: Variations Across Childhood Predictors (Germany)**

| Variable | IRR | SE | Prob | LCI | UCI | Global p-value |
| --- | --- | --- | --- | --- | --- | --- |
| Relationship with Mother (Ref: Very/Somewhat Bad) |  |  |  |  |  |  |
| Very/Somewhat Good | 1.16 | 0.13 | 0.21 | 0.92 | 1.45 | 0.21 |
| Relationship with Father (Ref: Very/Somewhat Bad) |  |  |  |  |  |  |
| Very/Somewhat Good | 0.98 | 0.09 | 0.79 | 0.81 | 1.17 | 0.79 |
| Parent Marital Status (Ref: Married) |  |  |  |  |  |  |
| Divorced | 0.83 | 0.09 | 0.07 | 0.67 | 1.02 | 0.18 |
| Never Married | 0.99 | 0.13 | 0.97 | 0.76 | 1.30 | . |
| One or Both Had Died | 1.20 | 0.20 | 0.25 | 0.88 | 1.66 | . |
| Childhood Income (Ref: Got By) |  |  |  |  |  |  |
| Lived Comfortably | 1.15 | 0.08 | 0.05 | 1.00 | 1.32 | 0.00 |
| Found it Difficult | 0.95 | 0.09 | 0.60 | 0.80 | 1.14 | . |
| Found it Very Difficult | 0.59 | 0.11 | 0.01 | 0.40 | 0.86 | . |
| Childhood Abuse (Ref: No) |  |  |  |  |  |  |
| Yes | 1.08 | 0.09 | 0.36 | 0.91 | 1.29 | 0.36 |
| Outsider (Ref: No) |  |  |  |  |  |  |
| Yes | 1.07 | 0.09 | 0.41 | 0.91 | 1.27 | 0.41 |
| Childhood Health (Ref: Good) |  |  |  |  |  |  |
| Excellent | 0.84 | 0.07 | 0.05 | 0.71 | 1.00 | 0.29 |
| Very Good | 0.94 | 0.07 | 0.39 | 0.81 | 1.09 | . |
| Fair | 1.03 | 0.12 | 0.79 | 0.82 | 1.31 | . |
| Poor | 1.12 | 0.25 | 0.60 | 0.73 | 1.72 | . |
| Immigration Status (Ref: Born in This Country) |  |  |  |  |  |  |
| Born in Another Country | 1.44 | 0.15 | 0.00 | 1.18 | 1.76 | 0.00 |
| Childhood Service Attendance (Ref: Never) |  |  |  |  |  |  |
| At Least 1/Week | 2.85 | 0.29 | 0.00 | 2.33 | 3.48 | 0.00 |
| 1-3/Month | 2.48 | 0.26 | 0.00 | 2.02 | 3.05 | . |
| <1/Month | 1.39 | 0.15 | 0.00 | 1.12 | 1.71 | . |
| Gender (Ref: Male) |  |  |  |  |  |  |
| Female | 1.13 | 0.07 | 0.04 | 1.00 | 1.26 | 0.11 |
| Other | 0.61 | 0.54 | 0.57 | 0.11 | 3.42 | . |
| Year of Birth (Ref: 1998-2005) |  |  |  |  |  |  |
| 1993-1998; Age 25-29 | 1.01 | 0.16 | 0.96 | 0.74 | 1.37 | 0.01 |
| 1983-1993; Age 30-39 | 1.00 | 0.14 | 0.97 | 0.76 | 1.31 | . |
| 1973-1983; Age 40-49 | 1.19 | 0.16 | 0.19 | 0.91 | 1.56 | . |
| 1963-1973; Age 50-59 | 1.14 | 0.16 | 0.34 | 0.87 | 1.49 | . |
| 1953-1963; Age 60-69 | 1.17 | 0.16 | 0.25 | 0.90 | 1.53 | . |
| 1943-1953; Age 70-79 | 1.42 | 0.20 | 0.01 | 1.07 | 1.88 | . |
| 1943 or Earlier; Age 80 or Older | 1.78 | 0.34 | 0.00 | 1.22 | 2.60 | . |
| Mother Absence/Presence (Ref: Present) |  |  |  |  |  |  |
| Absent | 0.98 | 0.14 | 0.87 | 0.75 | 1.28 | 0.87 |
| Father Absence/Presence (Ref: Present) |  |  |  |  |  |  |
| Absent | 1.03 | 0.12 | 0.83 | 0.81 | 1.30 | 0.83 |
| Childhood Religion (Ref: No Religion/Atheist/Agnostic) |  |  |  |  |  |  |
| Christianity | 1.73 | 0.16 | 0.00 | 1.45 | 2.07 | 0.00 |
| Islam | 3.14 | 0.40 | 0.00 | 2.44 | 4.04 | . |
| Some Other Religion | 2.84 | 0.55 | 0.00 | 1.94 | 4.16 | . |

**Table S5c: E-Values and E-Value Limits (Germany)**

| Variable | E-Value | E-Value Limit |
| --- | --- | --- |
| Relationship with Mother (Ref: Very/Somewhat Bad) |  |  |
| Very/Somewhat Good | 1.67 | 1.00 |
| Relationship with Father (Ref: Very/Somewhat Bad) |  |  |
| Very/Somewhat Good | 1.21 | 1.00 |
| Parent Marital Status (Ref: Married) |  |  |
| Divorced | 1.84 | 1.00 |
| Never Married | 1.09 | 1.00 |
| One or Both Had Died | 1.82 | 1.00 |
| Childhood Income (Ref: Got By) |  |  |
| Lived Comfortably | 1.65 | 1.00 |
| Found it Difficult | 1.31 | 1.00 |
| Found it Very Difficult | 3.24 | 1.38 |
| Childhood Abuse (Ref: No) |  |  |
| Yes | 1.44 | 1.00 |
| Outsider (Ref: No) |  |  |
| Yes | 1.40 | 1.00 |
| Childhood Health (Ref: Good) |  |  |
| Excellent | 1.76 | 1.00 |
| Very Good | 1.38 | 1.00 |
| Fair | 1.25 | 1.00 |
| Poor | 1.56 | 1.00 |
| Immigration Status (Ref: Born in This Country) |  |  |
| Born in Another Country | 2.50 | 1.40 |
| Childhood Service Attendance (Ref: Never) |  |  |
| At Least 1/Week | 6.59 | 2.42 |
| 1-3/Month | 5.49 | 2.19 |
| <1/Month | 2.34 | 1.31 |
| Gender (Ref: Male) |  |  |
| Female | 1.58 | 1.04 |
| Other | 3.06 | 1.00 |
| Year of Birth (Ref: 1998-2005) |  |  |
| 1993-1998; Age 25-29 | 1.11 | 1.00 |
| 1983-1993; Age 30-39 | 1.08 | 1.00 |
| 1973-1983; Age 40-49 | 1.78 | 1.00 |
| 1963-1973; Age 50-59 | 1.62 | 1.00 |
| 1953-1963; Age 60-69 | 1.72 | 1.00 |
| 1943-1953; Age 70-79 | 2.43 | 1.22 |
| 1943 or Earlier; Age 80 or Older | 3.44 | 1.44 |
| Mother Absence/Presence (Ref: Present) |  |  |
| Absent | 1.20 | 1.00 |
| Father Absence/Presence (Ref: Present) |  |  |
| Absent | 1.21 | 1.00 |
| Childhood Religion (Ref: No Religion/Atheist/Agnostic) |  |  |
| Christianity | 3.31 | 1.70 |
| Islam | 7.49 | 2.50 |
| Some Other Religion | 6.58 | 2.13 |

**Table S6a: Nationally-Representative Descriptive Statistics of the Observed Sample (Hong Kong)**

| Variable | Proportion | Frequency |
| --- | --- | --- |
| Relationship with Mother |  |  |
| Very Good | 0.36 | 1077 |
| Somewhat Good | 0.39 | 1164 |
| Somewhat Bad | 0.10 | 293 |
| Very Bad | 0.02 | 49 |
| Not Applicable | 0.14 | 426 |
| Missing | 0.00 | 3 |
| Relationship with Father |  |  |
| Very Good | 0.29 | 868 |
| Somewhat Good | 0.36 | 1089 |
| Somewhat Bad | 0.13 | 393 |
| Very Bad | 0.03 | 102 |
| Not Applicable | 0.19 | 557 |
| Missing | 0.00 | 3 |
| Parent Marital Status |  |  |
| Married | 0.91 | 2752 |
| Divorced | 0.04 | 114 |
| Never Married | 0.01 | 40 |
| One or Both Had Died | 0.02 | 50 |
| Missing | 0.02 | 56 |
| Childhood Income |  |  |
| Lived Comfortably | 0.30 | 906 |
| Got By | 0.51 | 1527 |
| Found it Difficult | 0.16 | 473 |
| Found it Very Difficult | 0.03 | 84 |
| Missing | 0.01 | 22 |
| Childhood Abuse |  |  |
| Yes | 0.11 | 318 |
| No | 0.89 | 2688 |
| Missing | 0.00 | 5 |
| Outsider |  |  |
| Yes | 0.22 | 664 |
| No | 0.74 | 2224 |
| Not Applicable | 0.04 | 110 |
| Missing | 0.00 | 14 |
| Childhood Health |  |  |
| Excellent | 0.18 | 545 |
| Very Good | 0.36 | 1073 |
| Good | 0.29 | 863 |
| Fair | 0.14 | 426 |
| Poor | 0.03 | 91 |
| Missing | 0.00 | 13 |
| Immigration Status |  |  |
| Born in This Country | 0.88 | 2637 |
| Born in Another Country | 0.11 | 321 |
| Missing | 0.02 | 53 |
| Childhood Service Attendance |  |  |
| At Least 1/Week | 0.14 | 432 |
| 1-3/Month | 0.18 | 528 |
| <1/Month | 0.25 | 753 |
| Never | 0.43 | 1295 |
| Missing | 0.00 | 4 |
| Gender |  |  |
| Male | 0.46 | 1390 |
| Female | 0.54 | 1620 |
| Other | 0.00 | 2 |
| Missing | . | . |
| Year of Birth |  |  |
| 1998-2005; Age 18-24 | 0.07 | 217 |
| 1993-1998; Age 25-29 | 0.07 | 198 |
| 1983-1993; Age 30-39 | 0.17 | 507 |
| 1973-1983; Age 40-49 | 0.19 | 580 |
| 1963-1973; Age 50-59 | 0.24 | 711 |
| 1953-1963; Age 60-69 | 0.21 | 620 |
| 1943-1953; Age 70-79 | 0.05 | 164 |
| 1943 or Earlier; 80 or Older | 0.00 | 15 |
| Missing | . | . |
| Childhood Religion |  |  |
| Christianity | 0.24 | 715 |
| Islam | 0.03 | 86 |
| Hinduism | 0.01 | 27 |
| Buddhism | 0.11 | 323 |
| Judaism | 0.01 | 16 |
| Sikhism | 0.00 | 4 |
| Baha'i | . | . |
| Jainism | 0.00 | 1 |
| Shinto | 0.01 | 18 |
| Taoism | 0.03 | 81 |
| Confucianism | 0.00 | 10 |
| Primal, Animist, or Folk Religion | 0.00 | 15 |
| Spiritism | . | . |
| African-Derived | . | . |
| Chinese | 0.04 | 108 |
| Some Other Religion | 0.00 | 5 |
| No Religion/Atheist/Agnostic | 0.53 | 1601 |
| Missing | 0.00 | 1 |
| Race/Ethnicity |  |  |
| Chinese (Cantonese) | 0.64 | 1930 |
| Chinese (Chaoshan) | 0.07 | 201 |
| Chinese (Fujianese) | 0.04 | 117 |
| Chinese (Hakka) | 0.04 | 121 |
| Chinese (Shanghainese) | 0.03 | 89 |
| Chinese (Other Ethnicity) | 0.09 | 264 |
| East Asian (Korean, Japanese) | 0.00 | 10 |
| Southeast Asian (Filipino, Indonesian, Thailand) | 0.02 | 46 |
| South Asian (Indian, Nepalese, Pakistani) | 0.01 | 17 |
| Taiwanese | 0.00 | 14 |
| White | 0.00 | 15 |
| Other | 0.00 | 4 |
| Missing | 0.06 | 184 |

**Table S6b: Variations Across Childhood Predictors (Hong Kong)**

| Variable | IRR | SE | Prob | LCI | UCI | Global p-value |
| --- | --- | --- | --- | --- | --- | --- |
| Relationship with Mother (Ref: Very/Somewhat Bad) |  |  |  |  |  |  |
| Very/Somewhat Good | 1.06 | 0.11 | 0.56 | 0.86 | 1.31 | 0.56 |
| Relationship with Father (Ref: Very/Somewhat Bad) |  |  |  |  |  |  |
| Very/Somewhat Good | 0.85 | 0.09 | 0.11 | 0.69 | 1.04 | 0.11 |
| Parent Marital Status (Ref: Married) |  |  |  |  |  |  |
| Divorced | 1.05 | 0.16 | 0.74 | 0.78 | 1.43 | 0.39 |
| Never Married | 1.25 | 0.30 | 0.34 | 0.79 | 1.99 | . |
| One or Both Had Died | 0.54 | 0.24 | 0.16 | 0.22 | 1.27 | . |
| Childhood Income (Ref: Got By) |  |  |  |  |  |  |
| Lived Comfortably | 1.10 | 0.08 | 0.19 | 0.96 | 1.26 | 0.45 |
| Found it Difficult | 1.00 | 0.12 | 0.99 | 0.80 | 1.26 | . |
| Found it Very Difficult | 0.82 | 0.19 | 0.41 | 0.52 | 1.31 | . |
| Childhood Abuse (Ref: No) |  |  |  |  |  |  |
| Yes | 1.22 | 0.09 | 0.01 | 1.05 | 1.41 | 0.01 |
| Outsider (Ref: No) |  |  |  |  |  |  |
| Yes | 1.14 | 0.07 | 0.03 | 1.01 | 1.28 | 0.03 |
| Childhood Health (Ref: Good) |  |  |  |  |  |  |
| Excellent | 1.11 | 0.11 | 0.25 | 0.93 | 1.34 | 0.00 |
| Very Good | 1.04 | 0.09 | 0.63 | 0.88 | 1.24 | . |
| Fair | 0.48 | 0.09 | 0.00 | 0.33 | 0.69 | . |
| Poor | 0.66 | 0.19 | 0.15 | 0.38 | 1.17 | . |
| Immigration Status (Ref: Born in This Country) |  |  |  |  |  |  |
| Born in Another Country | 1.01 | 0.17 | 0.94 | 0.74 | 1.40 | 0.94 |
| Childhood Service Attendance (Ref: Never) |  |  |  |  |  |  |
| At Least 1/Week | 2.94 | 0.53 | 0.00 | 2.06 | 4.19 | 0.00 |
| 1-3/Month | 2.68 | 0.46 | 0.00 | 1.90 | 3.76 | . |
| <1/Month | 1.50 | 0.26 | 0.02 | 1.07 | 2.12 | . |
| Gender (Ref: Male) |  |  |  |  |  |  |
| Female | 0.95 | 0.06 | 0.41 | 0.85 | 1.07 | 0.00 |
| Other | 0.00 | 0.00 | 0.00 | 0.00 | 0.00 | . |
| Year of Birth (Ref: 1998-2005) |  |  |  |  |  |  |
| 1993-1998; Age 25-29 | 0.98 | 0.14 | 0.90 | 0.74 | 1.31 | 0.06 |
| 1983-1993; Age 30-39 | 0.97 | 0.12 | 0.83 | 0.77 | 1.23 | . |
| 1973-1983; Age 40-49 | 1.11 | 0.12 | 0.32 | 0.90 | 1.38 | . |
| 1963-1973; Age 50-59 | 1.28 | 0.13 | 0.01 | 1.05 | 1.56 | . |
| 1953-1963; Age 60-69 | 1.20 | 0.14 | 0.14 | 0.94 | 1.51 | . |
| 1943-1953; Age 70-79 | 1.14 | 0.28 | 0.59 | 0.70 | 1.86 | . |
| 1943 or Earlier; Age 80 or Older | 0.84 | 0.83 | 0.86 | 0.12 | 5.87 | . |
| Mother Absence/Presence (Ref: Present) |  |  |  |  |  |  |
| Absent | 1.13 | 0.16 | 0.38 | 0.86 | 1.48 | 0.38 |
| Father Absence/Presence (Ref: Present) |  |  |  |  |  |  |
| Absent | 0.70 | 0.11 | 0.03 | 0.51 | 0.96 | 0.03 |
| Childhood Religion (Ref: No Religion/Atheist/Agnostic) |  |  |  |  |  |  |
| Christianity | 3.77 | 0.59 | 0.00 | 2.77 | 5.11 | 0.00 |
| Buddhism | 3.59 | 0.54 | 0.00 | 2.66 | 4.83 | . |
| Chinese | 3.94 | 0.65 | 0.00 | 2.85 | 5.44 | . |
| Some Other Religion | 3.73 | 0.61 | 0.00 | 2.72 | 5.13 | . |
| Race/Ethnicity (Ref: Ethnic Plurality) |  |  |  |  |  |  |
| Ethnic Minority | 0.94 | 0.07 | 0.40 | 0.82 | 1.08 | 0.40 |

**Table S6c: E-Values and E-Value Limits (Hong Kong)**

| Variable | E-Value | E-Value Limit |
| --- | --- | --- |
| Relationship with Mother (Ref: Very/Somewhat Bad) |  |  |
| Very/Somewhat Good | 1.31 | 1.00 |
| Relationship with Father (Ref: Very/Somewhat Bad) |  |  |
| Very/Somewhat Good | 1.60 | 1.00 |
| Parent Marital Status (Ref: Married) |  |  |
| Divorced | 1.28 | 1.00 |
| Never Married | 1.75 | 1.00 |
| One or Both Had Died | 2.93 | 1.00 |
| Childhood Income (Ref: Got By) |  |  |
| Lived Comfortably | 1.39 | 1.00 |
| Found it Difficult | 1.03 | 1.00 |
| Found it Very Difficult | 1.67 | 1.00 |
| Childhood Abuse (Ref: No) |  |  |
| Yes | 1.68 | 1.18 |
| Outsider (Ref: No) |  |  |
| Yes | 1.49 | 1.07 |
| Childhood Health (Ref: Good) |  |  |
| Excellent | 1.44 | 1.00 |
| Very Good | 1.24 | 1.00 |
| Fair | 3.32 | 1.70 |
| Poor | 2.27 | 1.00 |
| Immigration Status (Ref: Born in This Country) |  |  |
| Born in Another Country | 1.12 | 1.00 |
| Childhood Service Attendance (Ref: Never) |  |  |
| At Least 1/Week | 4.78 | 2.23 |
| 1-3/Month | 4.33 | 2.10 |
| <1/Month | 2.26 | 1.21 |
| Gender (Ref: Male) |  |  |
| Female | 1.26 | 1.00 |
| Other | 198438.11 | 352.52 |
| Year of Birth (Ref: 1998-2005) |  |  |
| 1993-1998; Age 25-29 | 1.14 | 1.00 |
| 1983-1993; Age 30-39 | 1.18 | 1.00 |
| 1973-1983; Age 40-49 | 1.44 | 1.00 |
| 1963-1973; Age 50-59 | 1.81 | 1.19 |
| 1953-1963; Age 60-69 | 1.63 | 1.00 |
| 1943-1953; Age 70-79 | 1.52 | 1.00 |
| 1943 or Earlier; Age 80 or Older | 1.62 | 1.00 |
| Mother Absence/Presence (Ref: Present) |  |  |
| Absent | 1.48 | 1.00 |
| Father Absence/Presence (Ref: Present) |  |  |
| Absent | 2.12 | 1.17 |
| Childhood Religion (Ref: No Religion/Atheist/Agnostic) |  |  |
| Christianity | 6.14 | 2.72 |
| Buddhism | 5.85 | 2.65 |
| Chinese | 6.42 | 2.77 |
| Some Other Religion | 6.08 | 2.68 |
| Race/Ethnicity (Ref: Ethnic Plurality) |  |  |
| Ethnic Minority | 1.30 | 1.00 |

**Table S7a: Nationally-Representative Descriptive Statistics of the Observed Sample (India)**

| Variable | Proportion | Frequency |
| --- | --- | --- |
| Relationship with Mother |  |  |
| Very Good | 0.90 | 11465 |
| Somewhat Good | 0.06 | 788 |
| Somewhat Bad | 0.01 | 88 |
| Very Bad | 0.01 | 73 |
| Not Applicable | 0.02 | 269 |
| Missing | 0.01 | 82 |
| Relationship with Father |  |  |
| Very Good | 0.86 | 10923 |
| Somewhat Good | 0.08 | 995 |
| Somewhat Bad | 0.01 | 126 |
| Very Bad | 0.01 | 100 |
| Not Applicable | 0.04 | 481 |
| Missing | 0.01 | 140 |
| Parent Marital Status |  |  |
| Married | 0.44 | 5578 |
| Divorced | 0.02 | 236 |
| Never Married | 0.08 | 1055 |
| One or Both Had Died | 0.07 | 940 |
| Missing | 0.39 | 4956 |
| Childhood Income |  |  |
| Lived Comfortably | 0.39 | 4946 |
| Got By | 0.24 | 3010 |
| Found it Difficult | 0.21 | 2703 |
| Found it Very Difficult | 0.16 | 2035 |
| Missing | 0.01 | 70 |
| Childhood Abuse |  |  |
| Yes | 0.11 | 1468 |
| No | 0.82 | 10526 |
| Missing | 0.06 | 771 |
| Outsider |  |  |
| Yes | 0.15 | 1926 |
| No | 0.84 | 10780 |
| Not Applicable | 0.00 | 15 |
| Missing | 0.00 | 44 |
| Childhood Health |  |  |
| Excellent | 0.17 | 2182 |
| Very Good | 0.30 | 3882 |
| Good | 0.32 | 4028 |
| Fair | 0.17 | 2202 |
| Poor | 0.03 | 424 |
| Missing | 0.00 | 47 |
| Immigration Status |  |  |
| Born in This Country | 0.99 | 12629 |
| Born in Another Country | 0.01 | 110 |
| Missing | 0.00 | 26 |
| Childhood Service Attendance |  |  |
| At Least 1/Week | 0.41 | 5288 |
| 1-3/Month | 0.23 | 2959 |
| <1/Month | 0.21 | 2719 |
| Never | 0.12 | 1478 |
| Missing | 0.03 | 321 |
| Gender |  |  |
| Male | 0.51 | 6473 |
| Female | 0.49 | 6292 |
| Other | . | . |
| Missing | . | . |
| Year of Birth |  |  |
| 1998-2005; Age 18-24 | 0.20 | 2543 |
| 1993-1998; Age 25-29 | 0.13 | 1640 |
| 1983-1993; Age 30-39 | 0.24 | 3109 |
| 1973-1983; Age 40-49 | 0.18 | 2275 |
| 1963-1973; Age 50-59 | 0.12 | 1574 |
| 1953-1963; Age 60-69 | 0.09 | 1188 |
| 1943-1953; Age 70-79 | 0.03 | 370 |
| 1943 or Earlier; 80 or Older | 0.01 | 67 |
| Missing | . | . |
| Childhood Religion |  |  |
| Christianity | 0.02 | 254 |
| Islam | 0.12 | 1550 |
| Hinduism | 0.82 | 10417 |
| Buddhism | 0.01 | 180 |
| Judaism | . | . |
| Sikhism | 0.01 | 126 |
| Baha'i | . | . |
| Jainism | 0.00 | 9 |
| Shinto | 0.00 | 4 |
| Taoism | . | . |
| Confucianism | . | . |
| Primal, Animist, or Folk Religion | 0.00 | 27 |
| Spiritism | . | . |
| African-Derived | . | . |
| Chinese | . | . |
| Some Other Religion | 0.00 | 59 |
| No Religion/Atheist/Agnostic | 0.00 | 7 |
| Missing | 0.01 | 131 |
| Race/Ethnicity |  |  |
| General | 0.28 | 3538 |
| Other Backward Caste | 0.33 | 4177 |
| Schedule Caste | 0.28 | 3599 |
| Schedule Tribe | 0.09 | 1185 |
| Other | . | . |
| Missing | 0.02 | 267 |

**Table S7b: Variations Across Childhood Predictors (India)**

| Variable | IRR | SE | Prob | LCI | UCI | Global p-value |
| --- | --- | --- | --- | --- | --- | --- |
| Relationship with Mother (Ref: Very/Somewhat Bad) |  |  |  |  |  |  |
| Very/Somewhat Good | 1.06 | 0.07 | 0.36 | 0.93 | 1.21 | 0.36 |
| Relationship with Father (Ref: Very/Somewhat Bad) |  |  |  |  |  |  |
| Very/Somewhat Good | 1.00 | 0.05 | 0.99 | 0.90 | 1.11 | 0.99 |
| Parent Marital Status (Ref: Married) |  |  |  |  |  |  |
| Divorced | 1.15 | 0.06 | 0.01 | 1.03 | 1.28 | 0.01 |
| Never Married | 1.03 | 0.03 | 0.32 | 0.97 | 1.09 | . |
| One or Both Had Died | 1.05 | 0.03 | 0.04 | 1.00 | 1.11 | . |
| Childhood Income (Ref: Got By) |  |  |  |  |  |  |
| Lived Comfortably | 1.07 | 0.02 | 0.00 | 1.02 | 1.12 | 0.00 |
| Found it Difficult | 1.01 | 0.03 | 0.84 | 0.95 | 1.06 | . |
| Found it Very Difficult | 0.95 | 0.03 | 0.08 | 0.89 | 1.01 | . |
| Childhood Abuse (Ref: No) |  |  |  |  |  |  |
| Yes | 1.07 | 0.03 | 0.00 | 1.02 | 1.12 | 0.00 |
| Outsider (Ref: No) |  |  |  |  |  |  |
| Yes | 1.03 | 0.02 | 0.26 | 0.98 | 1.08 | 0.26 |
| Childhood Health (Ref: Good) |  |  |  |  |  |  |
| Excellent | 0.95 | 0.03 | 0.08 | 0.90 | 1.01 | 0.34 |
| Very Good | 0.99 | 0.02 | 0.69 | 0.95 | 1.03 | . |
| Fair | 0.98 | 0.02 | 0.36 | 0.93 | 1.03 | . |
| Poor | 0.95 | 0.05 | 0.27 | 0.86 | 1.04 | . |
| Immigration Status (Ref: Born in This Country) |  |  |  |  |  |  |
| Born in Another Country | 1.04 | 0.09 | 0.66 | 0.87 | 1.24 | 0.66 |
| Childhood Service Attendance (Ref: Never) |  |  |  |  |  |  |
| At Least 1/Week | 1.14 | 0.03 | 0.00 | 1.07 | 1.21 | 0.00 |
| 1-3/Month | 1.04 | 0.03 | 0.20 | 0.98 | 1.11 | . |
| <1/Month | 0.99 | 0.03 | 0.86 | 0.93 | 1.06 | . |
| Gender (Ref: Male) |  |  |  |  |  |  |
| Female | 1.10 | 0.02 | 0.00 | 1.07 | 1.14 | 0.00 |
| Other | 1.00 | . | . | . | . | . |
| Year of Birth (Ref: 1998-2005) |  |  |  |  |  |  |
| 1993-1998; Age 25-29 | 0.99 | 0.03 | 0.64 | 0.93 | 1.04 | 0.00 |
| 1983-1993; Age 30-39 | 1.07 | 0.03 | 0.01 | 1.02 | 1.12 | . |
| 1973-1983; Age 40-49 | 1.10 | 0.03 | 0.00 | 1.05 | 1.16 | . |
| 1963-1973; Age 50-59 | 1.14 | 0.03 | 0.00 | 1.08 | 1.21 | . |
| 1953-1963; Age 60-69 | 1.19 | 0.04 | 0.00 | 1.12 | 1.27 | . |
| 1943-1953; Age 70-79 | 1.16 | 0.05 | 0.00 | 1.06 | 1.27 | . |
| 1943 or Earlier; Age 80 or Older | 1.41 | 0.10 | 0.00 | 1.23 | 1.62 | . |
| Mother Absence/Presence (Ref: Present) |  |  |  |  |  |  |
| Absent | 1.05 | 0.07 | 0.44 | 0.93 | 1.19 | 0.44 |
| Father Absence/Presence (Ref: Present) |  |  |  |  |  |  |
| Absent | 0.98 | 0.06 | 0.79 | 0.88 | 1.10 | 0.79 |
| Childhood Religion (Ref: Hinduism) |  |  |  |  |  |  |
| Islam | 0.97 | 0.03 | 0.34 | 0.91 | 1.03 | 0.26 |
| Some Other Religion | 0.94 | 0.04 | 0.17 | 0.86 | 1.03 | . |
| Race/Ethnicity (Ref: Ethnic Plurality) |  |  |  |  |  |  |
| Ethnic Minority | 0.94 | 0.02 | 0.00 | 0.90 | 0.98 | 0.00 |

**Table S7c: E-Values and E-Value Limits (India)**

| Variable | E-Value | E-Value Limit |
| --- | --- | --- |
| Relationship with Mother (Ref: Very/Somewhat Bad) |  |  |
| Very/Somewhat Good | 1.24 | 1.00 |
| Relationship with Father (Ref: Very/Somewhat Bad) |  |  |
| Very/Somewhat Good | 1.02 | 1.00 |
| Parent Marital Status (Ref: Married) |  |  |
| Divorced | 1.40 | 1.15 |
| Never Married | 1.15 | 1.00 |
| One or Both Had Died | 1.22 | 1.03 |
| Childhood Income (Ref: Got By) |  |  |
| Lived Comfortably | 1.25 | 1.12 |
| Found it Difficult | 1.06 | 1.00 |
| Found it Very Difficult | 1.23 | 1.00 |
| Childhood Abuse (Ref: No) |  |  |
| Yes | 1.25 | 1.11 |
| Outsider (Ref: No) |  |  |
| Yes | 1.15 | 1.00 |
| Childhood Health (Ref: Good) |  |  |
| Excellent | 1.22 | 1.00 |
| Very Good | 1.08 | 1.00 |
| Fair | 1.13 | 1.00 |
| Poor | 1.22 | 1.00 |
| Immigration Status (Ref: Born in This Country) |  |  |
| Born in Another Country | 1.18 | 1.00 |
| Childhood Service Attendance (Ref: Never) |  |  |
| At Least 1/Week | 1.38 | 1.22 |
| 1-3/Month | 1.19 | 1.00 |
| <1/Month | 1.06 | 1.00 |
| Gender (Ref: Male) |  |  |
| Female | 1.32 | 1.22 |
| Other | 1.00 | 1.00 |
| Year of Birth (Ref: 1998-2005) |  |  |
| 1993-1998; Age 25-29 | 1.10 | 1.00 |
| 1983-1993; Age 30-39 | 1.25 | 1.10 |
| 1973-1983; Age 40-49 | 1.32 | 1.18 |
| 1963-1973; Age 50-59 | 1.39 | 1.24 |
| 1953-1963; Age 60-69 | 1.47 | 1.30 |
| 1943-1953; Age 70-79 | 1.42 | 1.20 |
| 1943 or Earlier; Age 80 or Older | 1.78 | 1.46 |
| Mother Absence/Presence (Ref: Present) |  |  |
| Absent | 1.21 | 1.00 |
| Father Absence/Presence (Ref: Present) |  |  |
| Absent | 1.11 | 1.00 |
| Childhood Religion (Ref: Hinduism) |  |  |
| Islam | 1.16 | 1.00 |
| Some Other Religion | 1.24 | 1.00 |
| Race/Ethnicity (Ref: Ethnic Plurality) |  |  |
| Ethnic Minority | 1.24 | 1.13 |

**Table S8a: Nationally-Representative Descriptive Statistics of the Observed Sample (Indonesia)**

| Variable | Proportion | Frequency |
| --- | --- | --- |
| Relationship with Mother |  |  |
| Very Good | 0.89 | 6238 |
| Somewhat Good | 0.08 | 583 |
| Somewhat Bad | 0.01 | 50 |
| Very Bad | 0.00 | 26 |
| Not Applicable | 0.01 | 68 |
| Missing | 0.00 | 27 |
| Relationship with Father |  |  |
| Very Good | 0.87 | 6067 |
| Somewhat Good | 0.09 | 628 |
| Somewhat Bad | 0.01 | 68 |
| Very Bad | 0.01 | 52 |
| Not Applicable | 0.02 | 115 |
| Missing | 0.01 | 61 |
| Parent Marital Status |  |  |
| Married | 0.79 | 5557 |
| Divorced | 0.06 | 448 |
| Never Married | 0.01 | 47 |
| One or Both Had Died | 0.11 | 735 |
| Missing | 0.03 | 205 |
| Childhood Income |  |  |
| Lived Comfortably | 0.49 | 3408 |
| Got By | 0.42 | 2955 |
| Found it Difficult | 0.06 | 439 |
| Found it Very Difficult | 0.03 | 181 |
| Missing | 0.00 | 9 |
| Childhood Abuse |  |  |
| Yes | 0.07 | 486 |
| No | 0.92 | 6427 |
| Missing | 0.01 | 79 |
| Outsider |  |  |
| Yes | 0.05 | 343 |
| No | 0.95 | 6639 |
| Not Applicable | 0.00 | 1 |
| Missing | 0.00 | 9 |
| Childhood Health |  |  |
| Excellent | 0.18 | 1246 |
| Very Good | 0.28 | 1968 |
| Good | 0.36 | 2490 |
| Fair | 0.18 | 1233 |
| Poor | 0.01 | 55 |
| Missing | 0.00 | 0 |
| Immigration Status |  |  |
| Born in This Country | 1.00 | 6958 |
| Born in Another Country | 0.00 | 34 |
| Missing | . | . |
| Childhood Service Attendance |  |  |
| At Least 1/Week | 0.77 | 5363 |
| 1-3/Month | 0.14 | 973 |
| <1/Month | 0.05 | 329 |
| Never | 0.04 | 275 |
| Missing | 0.01 | 51 |
| Gender |  |  |
| Male | 0.50 | 3461 |
| Female | 0.50 | 3513 |
| Other | 0.00 | 7 |
| Missing | 0.00 | 11 |
| Year of Birth |  |  |
| 1998-2005; Age 18-24 | 0.17 | 1216 |
| 1993-1998; Age 25-29 | 0.12 | 849 |
| 1983-1993; Age 30-39 | 0.23 | 1591 |
| 1973-1983; Age 40-49 | 0.23 | 1576 |
| 1963-1973; Age 50-59 | 0.17 | 1169 |
| 1953-1963; Age 60-69 | 0.07 | 490 |
| 1943-1953; Age 70-79 | 0.01 | 83 |
| 1943 or Earlier; 80 or Older | 0.00 | 17 |
| Missing | . | . |
| Childhood Religion |  |  |
| Christianity | 0.08 | 528 |
| Islam | 0.91 | 6373 |
| Hinduism | 0.01 | 75 |
| Buddhism | 0.00 | 5 |
| Judaism | . | . |
| Sikhism | . | . |
| Baha'i | . | . |
| Jainism | 0.00 | 1 |
| Shinto | . | . |
| Taoism | 0.00 | 0 |
| Confucianism | 0.00 | 1 |
| Primal, Animist, or Folk Religion | 0.00 | 0 |
| Spiritism | . | . |
| African-Derived | . | . |
| Chinese | . | . |
| Some Other Religion | . | . |
| No Religion/Atheist/Agnostic | 0.00 | 2 |
| Missing | 0.00 | 8 |
| Race/Ethnicity |  |  |
| Banjar/Melayu Banjar | 0.05 | 320 |
| Betawi | 0.04 | 251 |
| Bugis | 0.03 | 243 |
| Jawa | 0.41 | 2846 |
| Madura | 0.04 | 262 |
| Minangkabau | 0.04 | 273 |
| Sunda/Parahyangan | 0.17 | 1172 |
| Bali | 0.01 | 69 |
| Batak | 0.02 | 165 |
| Makasar | 0.01 | 91 |
| Other | 0.18 | 1262 |
| Missing | 0.01 | 38 |

**Table S8b: Variations Across Childhood Predictors (Indonesia)**

| Variable | IRR | SE | Prob | LCI | UCI | Global p-value |
| --- | --- | --- | --- | --- | --- | --- |
| Relationship with Mother (Ref: Very/Somewhat Bad) |  |  |  |  |  |  |
| Very/Somewhat Good | 1.12 | 0.11 | 0.26 | 0.92 | 1.35 | 0.26 |
| Relationship with Father (Ref: Very/Somewhat Bad) |  |  |  |  |  |  |
| Very/Somewhat Good | 1.01 | 0.05 | 0.83 | 0.92 | 1.12 | 0.83 |
| Parent Marital Status (Ref: Married) |  |  |  |  |  |  |
| Divorced | 0.94 | 0.03 | 0.06 | 0.89 | 1.00 | 0.14 |
| Never Married | 0.89 | 0.10 | 0.31 | 0.70 | 1.12 | . |
| One or Both Had Died | 0.98 | 0.03 | 0.44 | 0.93 | 1.03 | . |
| Childhood Income (Ref: Got By) |  |  |  |  |  |  |
| Lived Comfortably | 1.03 | 0.01 | 0.05 | 1.00 | 1.06 | 0.26 |
| Found it Difficult | 1.00 | 0.03 | 0.95 | 0.94 | 1.07 | . |
| Found it Very Difficult | 1.00 | 0.05 | 0.94 | 0.90 | 1.10 | . |
| Childhood Abuse (Ref: No) |  |  |  |  |  |  |
| Yes | 0.97 | 0.03 | 0.31 | 0.91 | 1.03 | 0.31 |
| Outsider (Ref: No) |  |  |  |  |  |  |
| Yes | 0.95 | 0.04 | 0.20 | 0.87 | 1.03 | 0.20 |
| Childhood Health (Ref: Good) |  |  |  |  |  |  |
| Excellent | 1.03 | 0.02 | 0.11 | 0.99 | 1.07 | 0.06 |
| Very Good | 1.02 | 0.02 | 0.13 | 0.99 | 1.06 | . |
| Fair | 0.97 | 0.02 | 0.19 | 0.94 | 1.01 | . |
| Poor | 0.91 | 0.12 | 0.47 | 0.70 | 1.18 | . |
| Immigration Status (Ref: Born in This Country) |  |  |  |  |  |  |
| Born in Another Country | 0.80 | 0.13 | 0.18 | 0.58 | 1.10 | 0.18 |
| Childhood Service Attendance (Ref: Never) |  |  |  |  |  |  |
| At Least 1/Week | 1.15 | 0.05 | 0.00 | 1.06 | 1.24 | 0.00 |
| 1-3/Month | 1.08 | 0.05 | 0.08 | 0.99 | 1.17 | . |
| <1/Month | 1.02 | 0.05 | 0.70 | 0.92 | 1.13 | . |
| Gender (Ref: Male) |  |  |  |  |  |  |
| Female | 1.10 | 0.02 | 0.00 | 1.07 | 1.13 | 0.00 |
| Other | 1.15 | 0.11 | 0.14 | 0.96 | 1.37 | . |
| Year of Birth (Ref: 1998-2005) |  |  |  |  |  |  |
| 1993-1998; Age 25-29 | 1.03 | 0.03 | 0.25 | 0.98 | 1.09 | 0.00 |
| 1983-1993; Age 30-39 | 1.05 | 0.02 | 0.02 | 1.01 | 1.10 | . |
| 1973-1983; Age 40-49 | 1.07 | 0.03 | 0.00 | 1.03 | 1.13 | . |
| 1963-1973; Age 50-59 | 1.12 | 0.03 | 0.00 | 1.07 | 1.17 | . |
| 1953-1963; Age 60-69 | 1.11 | 0.04 | 0.00 | 1.04 | 1.19 | . |
| 1943-1953; Age 70-79 | 1.10 | 0.07 | 0.12 | 0.98 | 1.24 | . |
| 1943 or Earlier; Age 80 or Older | 1.33 | 0.07 | 0.00 | 1.20 | 1.47 | . |
| Mother Absence/Presence (Ref: Present) |  |  |  |  |  |  |
| Absent | 0.95 | 0.09 | 0.58 | 0.80 | 1.14 | 0.58 |
| Father Absence/Presence (Ref: Present) |  |  |  |  |  |  |
| Absent | 1.09 | 0.06 | 0.14 | 0.97 | 1.22 | 0.14 |
| Childhood Religion (Ref: Islam) |  |  |  |  |  |  |
| Christianity | 0.84 | 0.04 | 0.00 | 0.77 | 0.91 | 0.00 |
| Some Other Religion | 0.84 | 0.07 | 0.04 | 0.71 | 0.99 | . |
| Race/Ethnicity (Ref: Ethnic Plurality) |  |  |  |  |  |  |
| Ethnic Minority | 1.01 | 0.01 | 0.45 | 0.98 | 1.04 | 0.45 |

**Table S8c: E-Values and E-Value Limits (Indonesia)**

| Variable | E-Value | E-Value Limit |
| --- | --- | --- |
| Relationship with Mother (Ref: Very/Somewhat Bad) |  |  |
| Very/Somewhat Good | 1.32 | 1.00 |
| Relationship with Father (Ref: Very/Somewhat Bad) |  |  |
| Very/Somewhat Good | 1.08 | 1.00 |
| Parent Marital Status (Ref: Married) |  |  |
| Divorced | 1.21 | 1.00 |
| Never Married | 1.33 | 1.00 |
| One or Both Had Died | 1.12 | 1.00 |
| Childhood Income (Ref: Got By) |  |  |
| Lived Comfortably | 1.14 | 1.00 |
| Found it Difficult | 1.04 | 1.00 |
| Found it Very Difficult | 1.05 | 1.00 |
| Childhood Abuse (Ref: No) |  |  |
| Yes | 1.15 | 1.00 |
| Outsider (Ref: No) |  |  |
| Yes | 1.20 | 1.00 |
| Childhood Health (Ref: Good) |  |  |
| Excellent | 1.15 | 1.00 |
| Very Good | 1.13 | 1.00 |
| Fair | 1.14 | 1.00 |
| Poor | 1.29 | 1.00 |
| Immigration Status (Ref: Born in This Country) |  |  |
| Born in Another Country | 1.50 | 1.00 |
| Childhood Service Attendance (Ref: Never) |  |  |
| At Least 1/Week | 1.37 | 1.21 |
| 1-3/Month | 1.25 | 1.00 |
| <1/Month | 1.12 | 1.00 |
| Gender (Ref: Male) |  |  |
| Female | 1.28 | 1.21 |
| Other | 1.36 | 1.00 |
| Year of Birth (Ref: 1998-2005) |  |  |
| 1993-1998; Age 25-29 | 1.15 | 1.00 |
| 1983-1993; Age 30-39 | 1.20 | 1.06 |
| 1973-1983; Age 40-49 | 1.24 | 1.12 |
| 1963-1973; Age 50-59 | 1.32 | 1.22 |
| 1953-1963; Age 60-69 | 1.31 | 1.16 |
| 1943-1953; Age 70-79 | 1.29 | 1.00 |
| 1943 or Earlier; Age 80 or Older | 1.61 | 1.42 |
| Mother Absence/Presence (Ref: Present) |  |  |
| Absent | 1.20 | 1.00 |
| Father Absence/Presence (Ref: Present) |  |  |
| Absent | 1.27 | 1.00 |
| Childhood Religion (Ref: Islam) |  |  |
| Christianity | 1.44 | 1.28 |
| Some Other Religion | 1.43 | 1.07 |
| Race/Ethnicity (Ref: Ethnic Plurality) |  |  |
| Ethnic Minority | 1.08 | 1.00 |

**Table S9a: Nationally-Representative Descriptive Statistics of the Observed Sample (Israel)**

| Variable | Proportion | Frequency |
| --- | --- | --- |
| Relationship with Mother |  |  |
| Very Good | 0.73 | 2686 |
| Somewhat Good | 0.22 | 793 |
| Somewhat Bad | 0.03 | 110 |
| Very Bad | 0.00 | 18 |
| Not Applicable | 0.01 | 45 |
| Missing | 0.00 | 17 |
| Relationship with Father |  |  |
| Very Good | 0.62 | 2290 |
| Somewhat Good | 0.25 | 912 |
| Somewhat Bad | 0.06 | 234 |
| Very Bad | 0.01 | 37 |
| Not Applicable | 0.05 | 171 |
| Missing | 0.01 | 25 |
| Parent Marital Status |  |  |
| Married | 0.86 | 3172 |
| Divorced | 0.08 | 284 |
| Never Married | 0.01 | 36 |
| One or Both Had Died | 0.04 | 130 |
| Missing | 0.01 | 47 |
| Childhood Income |  |  |
| Lived Comfortably | 0.25 | 923 |
| Got By | 0.50 | 1822 |
| Found it Difficult | 0.18 | 667 |
| Found it Very Difficult | 0.07 | 239 |
| Missing | 0.00 | 17 |
| Childhood Abuse |  |  |
| Yes | . | . |
| No | . | . |
| Missing | . | . |
| Outsider |  |  |
| Yes | 0.10 | 371 |
| No | 0.88 | 3228 |
| Not Applicable | 0.01 | 36 |
| Missing | 0.01 | 34 |
| Childhood Health |  |  |
| Excellent | 0.49 | 1785 |
| Very Good | 0.35 | 1284 |
| Good | 0.13 | 480 |
| Fair | 0.03 | 105 |
| Poor | 0.00 | 6 |
| Missing | 0.00 | 8 |
| Immigration Status |  |  |
| Born in This Country | 0.76 | 2796 |
| Born in Another Country | 0.24 | 868 |
| Missing | 0.00 | 5 |
| Childhood Service Attendance |  |  |
| At Least 1/Week | 0.24 | 867 |
| 1-3/Month | 0.12 | 435 |
| <1/Month | 0.22 | 810 |
| Never | 0.42 | 1539 |
| Missing | 0.00 | 17 |
| Gender |  |  |
| Male | 0.49 | 1791 |
| Female | 0.51 | 1872 |
| Other | 0.00 | 0 |
| Missing | 0.00 | 6 |
| Year of Birth |  |  |
| 1998-2005; Age 18-24 | 0.15 | 553 |
| 1993-1998; Age 25-29 | 0.11 | 407 |
| 1983-1993; Age 30-39 | 0.18 | 666 |
| 1973-1983; Age 40-49 | 0.17 | 616 |
| 1963-1973; Age 50-59 | 0.15 | 542 |
| 1953-1963; Age 60-69 | 0.13 | 469 |
| 1943-1953; Age 70-79 | 0.09 | 336 |
| 1943 or Earlier; 80 or Older | 0.02 | 79 |
| Missing | . | . |
| Childhood Religion |  |  |
| Christianity | 0.02 | 60 |
| Islam | 0.18 | 647 |
| Hinduism | . | . |
| Buddhism | . | . |
| Judaism | 0.78 | 2873 |
| Sikhism | 0.00 | 1 |
| Baha'i | 0.00 | 1 |
| Jainism | . | . |
| Shinto | . | . |
| Taoism | . | . |
| Confucianism | . | . |
| Primal, Animist, or Folk Religion | 0.00 | 3 |
| Spiritism | . | . |
| African-Derived | . | . |
| Chinese | . | . |
| Some Other Religion | 0.00 | 5 |
| No Religion/Atheist/Agnostic | 0.02 | 69 |
| Missing | 0.00 | 10 |
| Race/Ethnicity |  |  |
| Jewish | 0.80 | 2926 |
| Arab | 0.18 | 674 |
| Other | 0.01 | 39 |
| Missing | 0.01 | 30 |

**Table S9b: Variations Across Childhood Predictors (Israel)**

| Variable | IRR | SE | Prob | LCI | UCI | Global p-value |
| --- | --- | --- | --- | --- | --- | --- |
| Relationship with Mother (Ref: Very/Somewhat Bad) |  |  |  |  |  |  |
| Very/Somewhat Good | 0.88 | 0.12 | 0.37 | 0.67 | 1.16 | 0.37 |
| Relationship with Father (Ref: Very/Somewhat Bad) |  |  |  |  |  |  |
| Very/Somewhat Good | 0.97 | 0.11 | 0.77 | 0.78 | 1.20 | 0.77 |
| Parent Marital Status (Ref: Married) |  |  |  |  |  |  |
| Divorced | 0.83 | 0.09 | 0.10 | 0.67 | 1.04 | 0.03 |
| Never Married | 1.48 | 0.39 | 0.14 | 0.88 | 2.51 | . |
| One or Both Had Died | 0.72 | 0.11 | 0.04 | 0.53 | 0.98 | . |
| Childhood Income (Ref: Got By) |  |  |  |  |  |  |
| Lived Comfortably | 0.97 | 0.06 | 0.59 | 0.86 | 1.09 | 0.24 |
| Found it Difficult | 1.13 | 0.08 | 0.10 | 0.98 | 1.29 | . |
| Found it Very Difficult | 0.93 | 0.10 | 0.53 | 0.75 | 1.16 | . |
| Childhood Abuse (Ref: No) |  |  |  |  |  |  |
| Yes | 1.00 | . | . | . | . | . |
| Outsider (Ref: No) |  |  |  |  |  |  |
| Yes | 1.06 | 0.10 | 0.50 | 0.89 | 1.28 | 0.50 |
| Childhood Health (Ref: Good) |  |  |  |  |  |  |
| Excellent | 1.44 | 0.14 | 0.00 | 1.18 | 1.76 | 0.00 |
| Very Good | 1.30 | 0.12 | 0.01 | 1.08 | 1.56 | . |
| Fair | 0.69 | 0.14 | 0.06 | 0.46 | 1.02 | . |
| Poor | 3.43 | 0.68 | 0.00 | 2.31 | 5.07 | . |
| Immigration Status (Ref: Born in This Country) |  |  |  |  |  |  |
| Born in Another Country | 0.74 | 0.06 | 0.00 | 0.63 | 0.88 | 0.00 |
| Childhood Service Attendance (Ref: Never) |  |  |  |  |  |  |
| At Least 1/Week | 3.16 | 0.28 | 0.00 | 2.64 | 3.78 | 0.00 |
| 1-3/Month | 2.76 | 0.28 | 0.00 | 2.26 | 3.37 | . |
| <1/Month | 1.62 | 0.16 | 0.00 | 1.34 | 1.96 | . |
| Gender (Ref: Male) |  |  |  |  |  |  |
| Female | 1.11 | 0.06 | 0.05 | 1.00 | 1.23 | 0.00 |
| Other | 0.00 | 0.00 | 0.00 | 0.00 | 0.00 | . |
| Year of Birth (Ref: 1998-2005) |  |  |  |  |  |  |
| 1993-1998; Age 25-29 | 0.86 | 0.06 | 0.03 | 0.75 | 0.99 | 0.10 |
| 1983-1993; Age 30-39 | 0.85 | 0.06 | 0.03 | 0.73 | 0.98 | . |
| 1973-1983; Age 40-49 | 0.85 | 0.07 | 0.06 | 0.72 | 1.01 | . |
| 1963-1973; Age 50-59 | 1.00 | 0.08 | 0.96 | 0.86 | 1.18 | . |
| 1953-1963; Age 60-69 | 0.89 | 0.09 | 0.27 | 0.73 | 1.09 | . |
| 1943-1953; Age 70-79 | 0.77 | 0.10 | 0.05 | 0.60 | 1.00 | . |
| 1943 or Earlier; Age 80 or Older | 0.93 | 0.22 | 0.75 | 0.58 | 1.49 | . |
| Mother Absence/Presence (Ref: Present) |  |  |  |  |  |  |
| Absent | 1.19 | 0.23 | 0.36 | 0.82 | 1.74 | 0.36 |
| Father Absence/Presence (Ref: Present) |  |  |  |  |  |  |
| Absent | 0.95 | 0.15 | 0.74 | 0.69 | 1.31 | 0.74 |
| Childhood Religion (Ref: Judaism) |  |  |  |  |  |  |
| Islam | 1.42 | 0.52 | 0.34 | 0.68 | 2.93 | 0.00 |
| Some Other Religion | 0.55 | 0.21 | 0.11 | 0.26 | 1.15 | . |
| Race/Ethnicity (Ref: Ethnic Plurality) |  |  |  |  |  |  |
| Ethnic Minority | 1.13 | 0.41 | 0.73 | 0.55 | 2.32 | 0.73 |

**Table S9c: E-Values and E-Value Limits (Israel)**

| Variable | E-Value | E-Value Limit |
| --- | --- | --- |
| Relationship with Mother (Ref: Very/Somewhat Bad) |  |  |
| Very/Somewhat Good | 1.46 | 1.00 |
| Relationship with Father (Ref: Very/Somewhat Bad) |  |  |
| Very/Somewhat Good | 1.19 | 1.00 |
| Parent Marital Status (Ref: Married) |  |  |
| Divorced | 1.60 | 1.00 |
| Never Married | 2.11 | 1.00 |
| One or Both Had Died | 1.94 | 1.11 |
| Childhood Income (Ref: Got By) |  |  |
| Lived Comfortably | 1.19 | 1.00 |
| Found it Difficult | 1.44 | 1.00 |
| Found it Very Difficult | 1.31 | 1.00 |
| Childhood Abuse (Ref: No) |  |  |
| Yes | 1.00 | 1.00 |
| Outsider (Ref: No) |  |  |
| Yes | 1.29 | 1.00 |
| Childhood Health (Ref: Good) |  |  |
| Excellent | 2.04 | 1.40 |
| Very Good | 1.79 | 1.25 |
| Fair | 2.07 | 1.00 |
| Poor | 4.96 | 2.41 |
| Immigration Status (Ref: Born in This Country) |  |  |
| Born in Another Country | 1.87 | 1.33 |
| Childhood Service Attendance (Ref: Never) |  |  |
| At Least 1/Week | 4.60 | 2.64 |
| 1-3/Month | 4.05 | 2.37 |
| <1/Month | 2.34 | 1.58 |
| Gender (Ref: Male) |  |  |
| Female | 1.40 | 1.02 |
| Other | 5582.89 | 83.78 |
| Year of Birth (Ref: 1998-2005) |  |  |
| 1993-1998; Age 25-29 | 1.51 | 1.08 |
| 1983-1993; Age 30-39 | 1.56 | 1.11 |
| 1973-1983; Age 40-49 | 1.55 | 1.00 |
| 1963-1973; Age 50-59 | 1.06 | 1.00 |
| 1953-1963; Age 60-69 | 1.42 | 1.00 |
| 1943-1953; Age 70-79 | 1.77 | 1.00 |
| 1943 or Earlier; Age 80 or Older | 1.33 | 1.00 |
| Mother Absence/Presence (Ref: Present) |  |  |
| Absent | 1.58 | 1.00 |
| Father Absence/Presence (Ref: Present) |  |  |
| Absent | 1.26 | 1.00 |
| Childhood Religion (Ref: Judaism) |  |  |
| Islam | 2.00 | 1.00 |
| Some Other Religion | 2.67 | 1.00 |
| Race/Ethnicity (Ref: Ethnic Plurality) |  |  |
| Ethnic Minority | 1.46 | 1.00 |

**Table S10a: Nationally-Representative Descriptive Statistics of the Observed Sample (Japan)**

| Variable | Proportion | Frequency |
| --- | --- | --- |
| Relationship with Mother |  |  |
| Very Good | 0.27 | 5630 |
| Somewhat Good | 0.46 | 9461 |
| Somewhat Bad | 0.13 | 2750 |
| Very Bad | 0.04 | 799 |
| Not Applicable | 0.09 | 1838 |
| Missing | 0.00 | 66 |
| Relationship with Father |  |  |
| Very Good | 0.20 | 4156 |
| Somewhat Good | 0.44 | 9081 |
| Somewhat Bad | 0.17 | 3446 |
| Very Bad | 0.06 | 1223 |
| Not Applicable | 0.13 | 2580 |
| Missing | 0.00 | 57 |
| Parent Marital Status |  |  |
| Married | 0.86 | 17713 |
| Divorced | 0.05 | 1127 |
| Never Married | 0.03 | 591 |
| One or Both Had Died | 0.04 | 754 |
| Missing | 0.02 | 359 |
| Childhood Income |  |  |
| Lived Comfortably | 0.41 | 8320 |
| Got By | 0.43 | 8799 |
| Found it Difficult | 0.12 | 2398 |
| Found it Very Difficult | 0.05 | 973 |
| Missing | 0.00 | 52 |
| Childhood Abuse |  |  |
| Yes | 0.07 | 1482 |
| No | 0.92 | 18964 |
| Missing | 0.00 | 96 |
| Outsider |  |  |
| Yes | 0.10 | 1963 |
| No | 0.83 | 17136 |
| Not Applicable | 0.07 | 1402 |
| Missing | 0.00 | 42 |
| Childhood Health |  |  |
| Excellent | 0.13 | 2711 |
| Very Good | 0.35 | 7106 |
| Good | 0.33 | 6689 |
| Fair | 0.16 | 3199 |
| Poor | 0.04 | 758 |
| Missing | 0.00 | 80 |
| Immigration Status |  |  |
| Born in This Country | 0.95 | 19548 |
| Born in Another Country | 0.01 | 158 |
| Missing | 0.04 | 837 |
| Childhood Service Attendance |  |  |
| At Least 1/Week | 0.02 | 398 |
| 1-3/Month | 0.04 | 883 |
| <1/Month | 0.24 | 5023 |
| Never | 0.69 | 14117 |
| Missing | 0.01 | 123 |
| Gender |  |  |
| Male | 0.48 | 9847 |
| Female | 0.52 | 10602 |
| Other | 0.00 | 28 |
| Missing | 0.00 | 66 |
| Year of Birth |  |  |
| 1998-2005; Age 18-24 | 0.08 | 1589 |
| 1993-1998; Age 25-29 | 0.04 | 806 |
| 1983-1993; Age 30-39 | 0.14 | 2851 |
| 1973-1983; Age 40-49 | 0.16 | 3363 |
| 1963-1973; Age 50-59 | 0.18 | 3770 |
| 1953-1963; Age 60-69 | 0.20 | 4118 |
| 1943-1953; Age 70-79 | 0.17 | 3554 |
| 1943 or Earlier; 80 or Older | 0.02 | 493 |
| Missing | . | . |
| Childhood Religion |  |  |
| Christianity | 0.02 | 343 |
| Islam | 0.00 | 7 |
| Hinduism | 0.00 | 4 |
| Buddhism | 0.32 | 6536 |
| Judaism | . | . |
| Sikhism | . | . |
| Baha'i | 0.00 | 7 |
| Jainism | 0.00 | 1 |
| Shinto | 0.02 | 382 |
| Taoism | 0.00 | 14 |
| Confucianism | 0.00 | 25 |
| Primal, Animist, or Folk Religion | 0.00 | 13 |
| Spiritism | . | . |
| African-Derived | . | . |
| Chinese | . | . |
| Some Other Religion | 0.00 | 46 |
| No Religion/Atheist/Agnostic | 0.63 | 12950 |
| Missing | 0.01 | 215 |
| Race/Ethnicity |  |  |
| No Data | . | . |

**Table S10b: Variations Across Childhood Predictors (Japan)**

| Variable | IRR | SE | Prob | LCI | UCI | Global p-value |
| --- | --- | --- | --- | --- | --- | --- |
| Relationship with Mother (Ref: Very/Somewhat Bad) |  |  |  |  |  |  |
| Very/Somewhat Good | 1.05 | 0.08 | 0.49 | 0.91 | 1.21 | 0.49 |
| Relationship with Father (Ref: Very/Somewhat Bad) |  |  |  |  |  |  |
| Very/Somewhat Good | 1.19 | 0.08 | 0.01 | 1.05 | 1.36 | 0.01 |
| Parent Marital Status (Ref: Married) |  |  |  |  |  |  |
| Divorced | 1.32 | 0.14 | 0.01 | 1.07 | 1.63 | 0.00 |
| Never Married | 1.48 | 0.17 | 0.00 | 1.18 | 1.86 | . |
| One or Both Had Died | 1.78 | 0.19 | 0.00 | 1.44 | 2.19 | . |
| Childhood Income (Ref: Got By) |  |  |  |  |  |  |
| Lived Comfortably | 0.98 | 0.05 | 0.73 | 0.88 | 1.09 | 0.38 |
| Found it Difficult | 1.13 | 0.09 | 0.11 | 0.97 | 1.32 | . |
| Found it Very Difficult | 1.02 | 0.12 | 0.89 | 0.81 | 1.27 | . |
| Childhood Abuse (Ref: No) |  |  |  |  |  |  |
| Yes | 1.32 | 0.11 | 0.00 | 1.12 | 1.54 | 0.00 |
| Outsider (Ref: No) |  |  |  |  |  |  |
| Yes | 1.55 | 0.12 | 0.00 | 1.34 | 1.81 | 0.00 |
| Childhood Health (Ref: Good) |  |  |  |  |  |  |
| Excellent | 1.28 | 0.10 | 0.00 | 1.10 | 1.49 | 0.00 |
| Very Good | 1.14 | 0.07 | 0.03 | 1.01 | 1.28 | . |
| Fair | 0.92 | 0.08 | 0.30 | 0.78 | 1.08 | . |
| Poor | 1.38 | 0.16 | 0.00 | 1.10 | 1.73 | . |
| Immigration Status (Ref: Born in This Country) |  |  |  |  |  |  |
| Born in Another Country | 2.00 | 0.35 | 0.00 | 1.42 | 2.82 | 0.00 |
| Childhood Service Attendance (Ref: Never) |  |  |  |  |  |  |
| At Least 1/Week | 5.01 | 0.46 | 0.00 | 4.19 | 6.01 | 0.00 |
| 1-3/Month | 4.84 | 0.36 | 0.00 | 4.18 | 5.59 | . |
| <1/Month | 2.17 | 0.14 | 0.00 | 1.92 | 2.45 | . |
| Gender (Ref: Male) |  |  |  |  |  |  |
| Female | 1.02 | 0.05 | 0.73 | 0.92 | 1.12 | 0.15 |
| Other | 1.87 | 0.60 | 0.05 | 0.99 | 3.52 | . |
| Year of Birth (Ref: 1998-2005) |  |  |  |  |  |  |
| 1993-1998; Age 25-29 | 0.98 | 0.16 | 0.89 | 0.70 | 1.36 | 0.00 |
| 1983-1993; Age 30-39 | 0.95 | 0.12 | 0.71 | 0.75 | 1.22 | . |
| 1973-1983; Age 40-49 | 0.92 | 0.11 | 0.50 | 0.72 | 1.17 | . |
| 1963-1973; Age 50-59 | 1.26 | 0.15 | 0.04 | 1.01 | 1.59 | . |
| 1953-1963; Age 60-69 | 1.75 | 0.19 | 0.00 | 1.41 | 2.18 | . |
| 1943-1953; Age 70-79 | 1.94 | 0.22 | 0.00 | 1.56 | 2.41 | . |
| 1943 or Earlier; Age 80 or Older | 2.17 | 0.32 | 0.00 | 1.63 | 2.89 | . |
| Mother Absence/Presence (Ref: Present) |  |  |  |  |  |  |
| Absent | 1.10 | 0.10 | 0.28 | 0.93 | 1.30 | 0.28 |
| Father Absence/Presence (Ref: Present) |  |  |  |  |  |  |
| Absent | 0.79 | 0.07 | 0.01 | 0.67 | 0.94 | 0.01 |
| Childhood Religion (Ref: No Religion/Atheist/Agnostic) |  |  |  |  |  |  |
| Buddhism | 1.84 | 0.11 | 0.00 | 1.64 | 2.07 | 0.00 |
| Some Other Religion | 2.12 | 0.19 | 0.00 | 1.78 | 2.53 | . |

**Table S10c: E-Values and E-Value Limits (Japan)**

| Variable | E-Value | E-Value Limit |
| --- | --- | --- |
| Relationship with Mother (Ref: Very/Somewhat Bad) |  |  |
| Very/Somewhat Good | 1.38 | 1.00 |
| Relationship with Father (Ref: Very/Somewhat Bad) |  |  |
| Very/Somewhat Good | 1.99 | 1.18 |
| Parent Marital Status (Ref: Married) |  |  |
| Divorced | 2.50 | 1.22 |
| Never Married | 3.16 | 1.40 |
| One or Both Had Died | 4.46 | 1.69 |
| Childhood Income (Ref: Got By) |  |  |
| Lived Comfortably | 1.21 | 1.00 |
| Found it Difficult | 1.74 | 1.00 |
| Found it Very Difficult | 1.19 | 1.00 |
| Childhood Abuse (Ref: No) |  |  |
| Yes | 2.48 | 1.31 |
| Outsider (Ref: No) |  |  |
| Yes | 3.47 | 1.58 |
| Childhood Health (Ref: Good) |  |  |
| Excellent | 2.33 | 1.28 |
| Very Good | 1.75 | 1.07 |
| Fair | 1.56 | 1.00 |
| Poor | 2.74 | 1.28 |
| Immigration Status (Ref: Born in This Country) |  |  |
| Born in Another Country | 5.49 | 1.66 |
| Childhood Service Attendance (Ref: Never) |  |  |
| At Least 1/Week | 25.81 | 3.51 |
| 1-3/Month | 24.35 | 3.51 |
| <1/Month | 6.36 | 2.12 |
| Gender (Ref: Male) |  |  |
| Female | 1.19 | 1.00 |
| Other | 4.87 | 1.00 |
| Year of Birth (Ref: 1998-2005) |  |  |
| 1993-1998; Age 25-29 | 1.24 | 1.00 |
| 1983-1993; Age 30-39 | 1.37 | 1.00 |
| 1973-1983; Age 40-49 | 1.54 | 1.00 |
| 1963-1973; Age 50-59 | 2.27 | 1.07 |
| 1953-1963; Age 60-69 | 4.33 | 1.66 |
| 1943-1953; Age 70-79 | 5.21 | 1.80 |
| 1943 or Earlier; Age 80 or Older | 6.35 | 1.87 |
| Mother Absence/Presence (Ref: Present) |  |  |
| Absent | 1.59 | 1.00 |
| Father Absence/Presence (Ref: Present) |  |  |
| Absent | 2.25 | 1.21 |
| Childhood Religion (Ref: No Religion/Atheist/Agnostic) |  |  |
| Buddhism | 4.75 | 1.88 |
| Some Other Religion | 6.13 | 2.01 |

**Table S11a: Nationally-Representative Descriptive Statistics of the Observed Sample (Kenya)**

| Variable | Proportion | Frequency |
| --- | --- | --- |
| Relationship with Mother |  |  |
| Very Good | 0.83 | 9418 |
| Somewhat Good | 0.13 | 1435 |
| Somewhat Bad | 0.01 | 130 |
| Very Bad | 0.01 | 100 |
| Not Applicable | 0.02 | 240 |
| Missing | 0.01 | 66 |
| Relationship with Father |  |  |
| Very Good | 0.70 | 7958 |
| Somewhat Good | 0.17 | 1896 |
| Somewhat Bad | 0.02 | 216 |
| Very Bad | 0.02 | 220 |
| Not Applicable | 0.08 | 967 |
| Missing | 0.01 | 132 |
| Parent Marital Status |  |  |
| Married | 0.81 | 9238 |
| Divorced | 0.06 | 697 |
| Never Married | 0.06 | 681 |
| One or Both Had Died | 0.04 | 471 |
| Missing | 0.03 | 301 |
| Childhood Income |  |  |
| Lived Comfortably | 0.27 | 3026 |
| Got By | 0.29 | 3279 |
| Found it Difficult | 0.36 | 4071 |
| Found it Very Difficult | 0.09 | 994 |
| Missing | 0.00 | 19 |
| Childhood Abuse |  |  |
| Yes | 0.11 | 1300 |
| No | 0.88 | 10040 |
| Missing | 0.00 | 49 |
| Outsider |  |  |
| Yes | 0.11 | 1223 |
| No | 0.89 | 10114 |
| Not Applicable | 0.00 | 23 |
| Missing | 0.00 | 29 |
| Childhood Health |  |  |
| Excellent | 0.39 | 4449 |
| Very Good | 0.23 | 2598 |
| Good | 0.23 | 2582 |
| Fair | 0.12 | 1384 |
| Poor | 0.03 | 349 |
| Missing | 0.00 | 26 |
| Immigration Status |  |  |
| Born in This Country | 0.99 | 11270 |
| Born in Another Country | 0.01 | 117 |
| Missing | 0.00 | 2 |
| Childhood Service Attendance |  |  |
| At Least 1/Week | 0.81 | 9189 |
| 1-3/Month | 0.15 | 1687 |
| <1/Month | 0.02 | 236 |
| Never | 0.02 | 198 |
| Missing | 0.01 | 79 |
| Gender |  |  |
| Male | 0.49 | 5567 |
| Female | 0.51 | 5813 |
| Other | 0.00 | 2 |
| Missing | 0.00 | 7 |
| Year of Birth |  |  |
| 1998-2005; Age 18-24 | 0.25 | 2868 |
| 1993-1998; Age 25-29 | 0.18 | 2035 |
| 1983-1993; Age 30-39 | 0.23 | 2564 |
| 1973-1983; Age 40-49 | 0.15 | 1708 |
| 1963-1973; Age 50-59 | 0.09 | 1072 |
| 1953-1963; Age 60-69 | 0.06 | 710 |
| 1943-1953; Age 70-79 | 0.03 | 360 |
| 1943 or Earlier; 80 or Older | 0.01 | 67 |
| Missing | 0.00 | 5 |
| Childhood Religion |  |  |
| Christianity | 0.91 | 10369 |
| Islam | 0.08 | 916 |
| Hinduism | . | . |
| Buddhism | 0.00 | 5 |
| Judaism | 0.00 | 6 |
| Sikhism | 0.00 | 0 |
| Baha'i | 0.00 | 3 |
| Jainism | 0.00 | 1 |
| Shinto | . | . |
| Taoism | . | . |
| Confucianism | . | . |
| Primal, Animist, or Folk Religion | 0.00 | 13 |
| Spiritism | . | . |
| African-Derived | . | . |
| Chinese | . | . |
| Some Other Religion | 0.00 | 0 |
| No Religion/Atheist/Agnostic | 0.01 | 67 |
| Missing | 0.00 | 9 |
| Race/Ethnicity |  |  |
| Luhya | 0.17 | 1943 |
| Luo | 0.10 | 1120 |
| Kalenjin | 0.12 | 1377 |
| Kamba | 0.11 | 1299 |
| Kikuyu | 0.19 | 2118 |
| Kisii | 0.07 | 789 |
| Maasai | 0.02 | 237 |
| Meru | 0.06 | 630 |
| Kenan Somali/Somali | 0.03 | 396 |
| Miji Kenda Tribes | 0.06 | 708 |
| Embu | 0.02 | 197 |
| Other | 0.05 | 548 |
| Missing | 0.00 | 27 |

**Table S11b: Variations Across Childhood Predictors (Kenya)**

| Variable | IRR | SE | Prob | LCI | UCI | Global p-value |
| --- | --- | --- | --- | --- | --- | --- |
| Relationship with Mother (Ref: Very/Somewhat Bad) |  |  |  |  |  |  |
| Very/Somewhat Good | 1.01 | 0.03 | 0.79 | 0.95 | 1.07 | 0.79 |
| Relationship with Father (Ref: Very/Somewhat Bad) |  |  |  |  |  |  |
| Very/Somewhat Good | 1.05 | 0.02 | 0.04 | 1.00 | 1.10 | 0.04 |
| Parent Marital Status (Ref: Married) |  |  |  |  |  |  |
| Divorced | 1.00 | 0.02 | 0.86 | 0.96 | 1.04 | 0.01 |
| Never Married | 0.92 | 0.03 | 0.00 | 0.87 | 0.97 | . |
| One or Both Had Died | 1.02 | 0.02 | 0.36 | 0.98 | 1.07 | . |
| Childhood Income (Ref: Got By) |  |  |  |  |  |  |
| Lived Comfortably | 0.99 | 0.01 | 0.45 | 0.96 | 1.02 | 0.61 |
| Found it Difficult | 1.00 | 0.01 | 0.85 | 0.97 | 1.02 | . |
| Found it Very Difficult | 1.01 | 0.02 | 0.50 | 0.98 | 1.05 | . |
| Childhood Abuse (Ref: No) |  |  |  |  |  |  |
| Yes | 0.98 | 0.02 | 0.22 | 0.95 | 1.01 | 0.22 |
| Outsider (Ref: No) |  |  |  |  |  |  |
| Yes | 0.97 | 0.02 | 0.08 | 0.94 | 1.00 | 0.08 |
| Childhood Health (Ref: Good) |  |  |  |  |  |  |
| Excellent | 1.00 | 0.01 | 0.84 | 0.97 | 1.02 | 0.20 |
| Very Good | 0.97 | 0.01 | 0.04 | 0.94 | 1.00 | . |
| Fair | 0.99 | 0.02 | 0.70 | 0.96 | 1.03 | . |
| Poor | 0.98 | 0.03 | 0.55 | 0.92 | 1.05 | . |
| Immigration Status (Ref: Born in This Country) |  |  |  |  |  |  |
| Born in Another Country | 0.99 | 0.04 | 0.88 | 0.92 | 1.07 | 0.88 |
| Childhood Service Attendance (Ref: Never) |  |  |  |  |  |  |
| At Least 1/Week | 1.02 | 0.05 | 0.61 | 0.93 | 1.12 | 0.70 |
| 1-3/Month | 1.01 | 0.05 | 0.79 | 0.92 | 1.11 | . |
| <1/Month | 1.04 | 0.06 | 0.47 | 0.93 | 1.17 | . |
| Gender (Ref: Male) |  |  |  |  |  |  |
| Female | 1.08 | 0.01 | 0.00 | 1.06 | 1.10 | 0.00 |
| Other | 1.32 | 0.04 | 0.00 | 1.24 | 1.41 | . |
| Year of Birth (Ref: 1998-2005) |  |  |  |  |  |  |
| 1993-1998; Age 25-29 | 1.03 | 0.02 | 0.06 | 1.00 | 1.07 | 0.00 |
| 1983-1993; Age 30-39 | 1.08 | 0.02 | 0.00 | 1.05 | 1.12 | . |
| 1973-1983; Age 40-49 | 1.13 | 0.02 | 0.00 | 1.10 | 1.17 | . |
| 1963-1973; Age 50-59 | 1.14 | 0.02 | 0.00 | 1.09 | 1.18 | . |
| 1953-1963; Age 60-69 | 1.19 | 0.02 | 0.00 | 1.14 | 1.23 | . |
| 1943-1953; Age 70-79 | 1.14 | 0.04 | 0.00 | 1.06 | 1.23 | . |
| 1943 or Earlier; Age 80 or Older | 1.22 | 0.07 | 0.00 | 1.08 | 1.37 | . |
| Mother Absence/Presence (Ref: Present) |  |  |  |  |  |  |
| Absent | 1.02 | 0.03 | 0.50 | 0.96 | 1.09 | 0.50 |
| Father Absence/Presence (Ref: Present) |  |  |  |  |  |  |
| Absent | 1.03 | 0.02 | 0.21 | 0.98 | 1.08 | 0.21 |
| Childhood Religion (Ref: Christianity) |  |  |  |  |  |  |
| Islam | 1.01 | 0.02 | 0.66 | 0.97 | 1.05 | 0.65 |
| Some Other Religion | 0.93 | 0.08 | 0.42 | 0.78 | 1.11 | . |
| Race/Ethnicity (Ref: Ethnic Plurality) |  |  |  |  |  |  |
| Ethnic Minority | 1.02 | 0.02 | 0.21 | 0.99 | 1.06 | 0.21 |

**Table S11c: E-Values and E-Value Limits (Kenya)**

| Variable | E-Value | E-Value Limit |
| --- | --- | --- |
| Relationship with Mother (Ref: Very/Somewhat Bad) |  |  |
| Very/Somewhat Good | 1.07 | 1.00 |
| Relationship with Father (Ref: Very/Somewhat Bad) |  |  |
| Very/Somewhat Good | 1.19 | 1.04 |
| Parent Marital Status (Ref: Married) |  |  |
| Divorced | 1.05 | 1.00 |
| Never Married | 1.27 | 1.14 |
| One or Both Had Died | 1.12 | 1.00 |
| Childhood Income (Ref: Got By) |  |  |
| Lived Comfortably | 1.08 | 1.00 |
| Found it Difficult | 1.04 | 1.00 |
| Found it Very Difficult | 1.09 | 1.00 |
| Childhood Abuse (Ref: No) |  |  |
| Yes | 1.11 | 1.00 |
| Outsider (Ref: No) |  |  |
| Yes | 1.15 | 1.00 |
| Childhood Health (Ref: Good) |  |  |
| Excellent | 1.04 | 1.00 |
| Very Good | 1.14 | 1.02 |
| Fair | 1.06 | 1.00 |
| Poor | 1.12 | 1.00 |
| Immigration Status (Ref: Born in This Country) |  |  |
| Born in Another Country | 1.06 | 1.00 |
| Childhood Service Attendance (Ref: Never) |  |  |
| At Least 1/Week | 1.13 | 1.00 |
| 1-3/Month | 1.09 | 1.00 |
| <1/Month | 1.18 | 1.00 |
| Gender (Ref: Male) |  |  |
| Female | 1.25 | 1.20 |
| Other | 1.60 | 1.47 |
| Year of Birth (Ref: 1998-2005) |  |  |
| 1993-1998; Age 25-29 | 1.15 | 1.00 |
| 1983-1993; Age 30-39 | 1.26 | 1.19 |
| 1973-1983; Age 40-49 | 1.35 | 1.27 |
| 1963-1973; Age 50-59 | 1.35 | 1.26 |
| 1953-1963; Age 60-69 | 1.43 | 1.34 |
| 1943-1953; Age 70-79 | 1.36 | 1.20 |
| 1943 or Earlier; Age 80 or Older | 1.47 | 1.24 |
| Mother Absence/Presence (Ref: Present) |  |  |
| Absent | 1.12 | 1.00 |
| Father Absence/Presence (Ref: Present) |  |  |
| Absent | 1.14 | 1.00 |
| Childhood Religion (Ref: Christianity) |  |  |
| Islam | 1.08 | 1.00 |
| Some Other Religion | 1.24 | 1.00 |
| Race/Ethnicity (Ref: Ethnic Plurality) |  |  |
| Ethnic Minority | 1.13 | 1.00 |

**Table S12a: Nationally-Representative Descriptive Statistics of the Observed Sample (Mexico)**

| Variable | Proportion | Frequency |
| --- | --- | --- |
| Relationship with Mother |  |  |
| Very Good | 0.68 | 3912 |
| Somewhat Good | 0.23 | 1340 |
| Somewhat Bad | 0.03 | 177 |
| Very Bad | 0.02 | 90 |
| Not Applicable | 0.03 | 177 |
| Missing | 0.01 | 80 |
| Relationship with Father |  |  |
| Very Good | 0.53 | 3089 |
| Somewhat Good | 0.27 | 1556 |
| Somewhat Bad | 0.06 | 335 |
| Very Bad | 0.05 | 267 |
| Not Applicable | 0.08 | 470 |
| Missing | 0.01 | 60 |
| Parent Marital Status |  |  |
| Married | 0.69 | 3999 |
| Divorced | 0.06 | 341 |
| Never Married | 0.14 | 827 |
| One or Both Had Died | 0.03 | 176 |
| Missing | 0.07 | 432 |
| Childhood Income |  |  |
| Lived Comfortably | 0.31 | 1775 |
| Got By | 0.32 | 1872 |
| Found it Difficult | 0.30 | 1712 |
| Found it Very Difficult | 0.06 | 369 |
| Missing | 0.01 | 48 |
| Childhood Abuse |  |  |
| Yes | 0.16 | 905 |
| No | 0.80 | 4604 |
| Missing | 0.05 | 267 |
| Outsider |  |  |
| Yes | 0.13 | 772 |
| No | 0.85 | 4897 |
| Not Applicable | 0.01 | 58 |
| Missing | 0.01 | 49 |
| Childhood Health |  |  |
| Excellent | 0.32 | 1860 |
| Very Good | 0.23 | 1350 |
| Good | 0.29 | 1677 |
| Fair | 0.13 | 743 |
| Poor | 0.02 | 133 |
| Missing | 0.00 | 14 |
| Immigration Status |  |  |
| Born in This Country | 0.96 | 5517 |
| Born in Another Country | 0.02 | 108 |
| Missing | 0.03 | 151 |
| Childhood Service Attendance |  |  |
| At Least 1/Week | 0.44 | 2514 |
| 1-3/Month | 0.20 | 1162 |
| <1/Month | 0.19 | 1087 |
| Never | 0.16 | 944 |
| Missing | 0.01 | 69 |
| Gender |  |  |
| Male | 0.48 | 2755 |
| Female | 0.52 | 2997 |
| Other | 0.00 | 3 |
| Missing | 0.00 | 21 |
| Year of Birth |  |  |
| 1998-2005; Age 18-24 | 0.17 | 986 |
| 1993-1998; Age 25-29 | 0.11 | 623 |
| 1983-1993; Age 30-39 | 0.23 | 1312 |
| 1973-1983; Age 40-49 | 0.18 | 1027 |
| 1963-1973; Age 50-59 | 0.15 | 873 |
| 1953-1963; Age 60-69 | 0.11 | 611 |
| 1943-1953; Age 70-79 | 0.05 | 277 |
| 1943 or Earlier; 80 or Older | 0.01 | 68 |
| Missing | . | . |
| Childhood Religion |  |  |
| Christianity | 0.92 | 5337 |
| Islam | 0.00 | 6 |
| Hinduism | 0.00 | 1 |
| Buddhism | 0.00 | 1 |
| Judaism | 0.00 | 8 |
| Sikhism | 0.00 | 4 |
| Baha'i | 0.00 | 1 |
| Jainism | . | . |
| Shinto | 0.00 | 2 |
| Taoism | 0.00 | 5 |
| Confucianism | . | . |
| Primal, Animist, or Folk Religion | 0.00 | 2 |
| Spiritism | . | . |
| African-Derived | . | . |
| Chinese | . | . |
| Some Other Religion | 0.00 | 7 |
| No Religion/Atheist/Agnostic | 0.06 | 328 |
| Missing | 0.01 | 74 |
| Race/Ethnicity |  |  |
| White | 0.19 | 1116 |
| Mestizo | 0.48 | 2762 |
| Indigenous | 0.10 | 594 |
| Black | 0.02 | 108 |
| Mulatto | 0.01 | 63 |
| Other | 0.06 | 339 |
| Missing | 0.14 | 794 |

**Table S12b: Variations Across Childhood Predictors (Mexico)**

| Variable | IRR | SE | Prob | LCI | UCI | Global p-value |
| --- | --- | --- | --- | --- | --- | --- |
| Relationship with Mother (Ref: Very/Somewhat Bad) |  |  |  |  |  |  |
| Very/Somewhat Good | 1.02 | 0.08 | 0.84 | 0.87 | 1.19 | 0.84 |
| Relationship with Father (Ref: Very/Somewhat Bad) |  |  |  |  |  |  |
| Very/Somewhat Good | 1.04 | 0.06 | 0.47 | 0.93 | 1.17 | 0.47 |
| Parent Marital Status (Ref: Married) |  |  |  |  |  |  |
| Divorced | 0.98 | 0.08 | 0.83 | 0.84 | 1.15 | 0.02 |
| Never Married | 0.83 | 0.05 | 0.00 | 0.73 | 0.94 | . |
| One or Both Had Died | 0.96 | 0.09 | 0.65 | 0.80 | 1.15 | . |
| Childhood Income (Ref: Got By) |  |  |  |  |  |  |
| Lived Comfortably | 1.04 | 0.05 | 0.36 | 0.96 | 1.14 | 0.05 |
| Found it Difficult | 1.04 | 0.05 | 0.42 | 0.95 | 1.13 | . |
| Found it Very Difficult | 1.20 | 0.08 | 0.01 | 1.06 | 1.37 | . |
| Childhood Abuse (Ref: No) |  |  |  |  |  |  |
| Yes | 0.99 | 0.05 | 0.91 | 0.90 | 1.10 | 0.91 |
| Outsider (Ref: No) |  |  |  |  |  |  |
| Yes | 1.00 | 0.05 | 0.96 | 0.90 | 1.11 | 0.96 |
| Childhood Health (Ref: Good) |  |  |  |  |  |  |
| Excellent | 1.04 | 0.04 | 0.42 | 0.95 | 1.13 | 0.17 |
| Very Good | 0.93 | 0.05 | 0.14 | 0.84 | 1.03 | . |
| Fair | 0.94 | 0.05 | 0.29 | 0.84 | 1.05 | . |
| Poor | 0.95 | 0.11 | 0.66 | 0.76 | 1.19 | . |
| Immigration Status (Ref: Born in This Country) |  |  |  |  |  |  |
| Born in Another Country | 0.84 | 0.14 | 0.29 | 0.61 | 1.16 | 0.29 |
| Childhood Service Attendance (Ref: Never) |  |  |  |  |  |  |
| At Least 1/Week | 1.57 | 0.10 | 0.00 | 1.39 | 1.77 | 0.00 |
| 1-3/Month | 1.44 | 0.09 | 0.00 | 1.27 | 1.64 | . |
| <1/Month | 0.98 | 0.08 | 0.80 | 0.84 | 1.15 | . |
| Gender (Ref: Male) |  |  |  |  |  |  |
| Female | 1.12 | 0.04 | 0.00 | 1.05 | 1.20 | 0.00 |
| Other | 1.40 | 0.52 | 0.36 | 0.68 | 2.91 | . |
| Year of Birth (Ref: 1998-2005) |  |  |  |  |  |  |
| 1993-1998; Age 25-29 | 1.26 | 0.11 | 0.01 | 1.07 | 1.48 | 0.00 |
| 1983-1993; Age 30-39 | 1.46 | 0.11 | 0.00 | 1.26 | 1.68 | . |
| 1973-1983; Age 40-49 | 1.69 | 0.12 | 0.00 | 1.46 | 1.95 | . |
| 1963-1973; Age 50-59 | 2.03 | 0.15 | 0.00 | 1.76 | 2.34 | . |
| 1953-1963; Age 60-69 | 1.98 | 0.15 | 0.00 | 1.70 | 2.31 | . |
| 1943-1953; Age 70-79 | 2.24 | 0.20 | 0.00 | 1.88 | 2.66 | . |
| 1943 or Earlier; Age 80 or Older | 2.22 | 0.26 | 0.00 | 1.77 | 2.79 | . |
| Mother Absence/Presence (Ref: Present) |  |  |  |  |  |  |
| Absent | 0.97 | 0.09 | 0.75 | 0.80 | 1.17 | 0.75 |
| Father Absence/Presence (Ref: Present) |  |  |  |  |  |  |
| Absent | 0.99 | 0.07 | 0.94 | 0.87 | 1.14 | 0.94 |
| Childhood Religion (Ref: No Religion/Atheist/Agnostic) |  |  |  |  |  |  |
| Christianity | 1.12 | 0.11 | 0.23 | 0.93 | 1.36 | 0.17 |
| Some Other Religion | 1.44 | 0.28 | 0.06 | 0.98 | 2.11 | . |
| Race/Ethnicity (Ref: Ethnic Plurality) |  |  |  |  |  |  |
| Ethnic Minority | 1.04 | 0.04 | 0.32 | 0.97 | 1.11 | 0.32 |

**Table S12c: E-Values and E-Value Limits (Mexico)**

| Variable | E-Value | E-Value Limit |
| --- | --- | --- |
| Relationship with Mother (Ref: Very/Somewhat Bad) |  |  |
| Very/Somewhat Good | 1.12 | 1.00 |
| Relationship with Father (Ref: Very/Somewhat Bad) |  |  |
| Very/Somewhat Good | 1.21 | 1.00 |
| Parent Marital Status (Ref: Married) |  |  |
| Divorced | 1.12 | 1.00 |
| Never Married | 1.55 | 1.22 |
| One or Both Had Died | 1.21 | 1.00 |
| Childhood Income (Ref: Got By) |  |  |
| Lived Comfortably | 1.21 | 1.00 |
| Found it Difficult | 1.19 | 1.00 |
| Found it Very Difficult | 1.54 | 1.20 |
| Childhood Abuse (Ref: No) |  |  |
| Yes | 1.07 | 1.00 |
| Outsider (Ref: No) |  |  |
| Yes | 1.05 | 1.00 |
| Childhood Health (Ref: Good) |  |  |
| Excellent | 1.19 | 1.00 |
| Very Good | 1.30 | 1.00 |
| Fair | 1.26 | 1.00 |
| Poor | 1.23 | 1.00 |
| Immigration Status (Ref: Born in This Country) |  |  |
| Born in Another Country | 1.53 | 1.00 |
| Childhood Service Attendance (Ref: Never) |  |  |
| At Least 1/Week | 2.12 | 1.64 |
| 1-3/Month | 1.93 | 1.51 |
| <1/Month | 1.14 | 1.00 |
| Gender (Ref: Male) |  |  |
| Female | 1.39 | 1.18 |
| Other | 1.87 | 1.00 |
| Year of Birth (Ref: 1998-2005) |  |  |
| 1993-1998; Age 25-29 | 1.64 | 1.22 |
| 1983-1993; Age 30-39 | 1.95 | 1.49 |
| 1973-1983; Age 40-49 | 2.28 | 1.71 |
| 1963-1973; Age 50-59 | 2.73 | 1.99 |
| 1953-1963; Age 60-69 | 2.67 | 1.94 |
| 1943-1953; Age 70-79 | 2.98 | 2.08 |
| 1943 or Earlier; Age 80 or Older | 2.96 | 2.00 |
| Mother Absence/Presence (Ref: Present) |  |  |
| Absent | 1.17 | 1.00 |
| Father Absence/Presence (Ref: Present) |  |  |
| Absent | 1.06 | 1.00 |
| Childhood Religion (Ref: No Religion/Atheist/Agnostic) |  |  |
| Christianity | 1.40 | 1.00 |
| Some Other Religion | 1.93 | 1.00 |
| Race/Ethnicity (Ref: Ethnic Plurality) |  |  |
| Ethnic Minority | 1.19 | 1.00 |

**Table S13a: Nationally-Representative Descriptive Statistics of the Observed Sample (Nigeria)**

| Variable | Proportion | Frequency |
| --- | --- | --- |
| Relationship with Mother |  |  |
| Very Good | 0.88 | 5986 |
| Somewhat Good | 0.09 | 648 |
| Somewhat Bad | 0.01 | 62 |
| Very Bad | 0.00 | 18 |
| Not Applicable | 0.02 | 104 |
| Missing | 0.00 | 9 |
| Relationship with Father |  |  |
| Very Good | 0.82 | 5578 |
| Somewhat Good | 0.14 | 924 |
| Somewhat Bad | 0.01 | 76 |
| Very Bad | 0.01 | 43 |
| Not Applicable | 0.03 | 177 |
| Missing | 0.00 | 29 |
| Parent Marital Status |  |  |
| Married | 0.82 | 5568 |
| Divorced | 0.05 | 307 |
| Never Married | 0.05 | 335 |
| One or Both Had Died | 0.07 | 462 |
| Missing | 0.02 | 154 |
| Childhood Income |  |  |
| Lived Comfortably | 0.32 | 2192 |
| Got By | 0.35 | 2381 |
| Found it Difficult | 0.24 | 1661 |
| Found it Very Difficult | 0.08 | 563 |
| Missing | 0.00 | 29 |
| Childhood Abuse |  |  |
| Yes | 0.13 | 880 |
| No | 0.86 | 5851 |
| Missing | 0.01 | 96 |
| Outsider |  |  |
| Yes | 0.10 | 669 |
| No | 0.89 | 6059 |
| Not Applicable | 0.01 | 86 |
| Missing | 0.00 | 13 |
| Childhood Health |  |  |
| Excellent | 0.39 | 2644 |
| Very Good | 0.38 | 2613 |
| Good | 0.17 | 1152 |
| Fair | 0.04 | 306 |
| Poor | 0.01 | 98 |
| Missing | 0.00 | 14 |
| Immigration Status |  |  |
| Born in This Country | 0.99 | 6779 |
| Born in Another Country | 0.01 | 47 |
| Missing | 0.00 | 1 |
| Childhood Service Attendance |  |  |
| At Least 1/Week | 0.87 | 5907 |
| 1-3/Month | 0.09 | 600 |
| <1/Month | 0.02 | 136 |
| Never | 0.02 | 138 |
| Missing | 0.01 | 45 |
| Gender |  |  |
| Male | 0.49 | 3371 |
| Female | 0.51 | 3456 |
| Other | 0.00 | 0 |
| Missing | . | . |
| Year of Birth |  |  |
| 1998-2005; Age 18-24 | 0.22 | 1533 |
| 1993-1998; Age 25-29 | 0.17 | 1193 |
| 1983-1993; Age 30-39 | 0.28 | 1943 |
| 1973-1983; Age 40-49 | 0.16 | 1059 |
| 1963-1973; Age 50-59 | 0.09 | 619 |
| 1953-1963; Age 60-69 | 0.04 | 296 |
| 1943-1953; Age 70-79 | 0.02 | 133 |
| 1943 or Earlier; 80 or Older | 0.01 | 50 |
| Missing | . | . |
| Childhood Religion |  |  |
| Christianity | 0.51 | 3463 |
| Islam | 0.49 | 3314 |
| Hinduism | . | . |
| Buddhism | 0.00 | 0 |
| Judaism | . | . |
| Sikhism | . | . |
| Baha'i | . | . |
| Jainism | . | . |
| Shinto | . | . |
| Taoism | . | . |
| Confucianism | 0.00 | 0 |
| Primal, Animist, or Folk Religion | 0.00 | 17 |
| Spiritism | . | . |
| African-Derived | . | . |
| Chinese | . | . |
| Some Other Religion | . | . |
| No Religion/Atheist/Agnostic | 0.00 | 19 |
| Missing | 0.00 | 14 |
| Race/Ethnicity |  |  |
| Hausa | 0.34 | 2342 |
| Yoruba | 0.18 | 1230 |
| Igbo (Ibo) | 0.16 | 1112 |
| Edo | 0.02 | 116 |
| Urhobo | 0.01 | 38 |
| Fulani | 0.04 | 266 |
| Kanuri | 0.00 | 31 |
| Tiv | 0.03 | 198 |
| Efik | 0.01 | 48 |
| Ijaw | 0.02 | 110 |
| Igala | 0.01 | 77 |
| Ibibio | 0.03 | 180 |
| Idoma | 0.01 | 61 |
| Other | 0.15 | 1014 |
| Missing | 0.00 | 4 |

**Table S13b: Variations Across Childhood Predictors (Nigeria)**

| Variable | IRR | SE | Prob | LCI | UCI | Global p-value |
| --- | --- | --- | --- | --- | --- | --- |
| Relationship with Mother (Ref: Very/Somewhat Bad) |  |  |  |  |  |  |
| Very/Somewhat Good | 1.07 | 0.07 | 0.35 | 0.93 | 1.22 | 0.35 |
| Relationship with Father (Ref: Very/Somewhat Bad) |  |  |  |  |  |  |
| Very/Somewhat Good | 0.99 | 0.03 | 0.81 | 0.93 | 1.06 | 0.81 |
| Parent Marital Status (Ref: Married) |  |  |  |  |  |  |
| Divorced | 0.93 | 0.03 | 0.03 | 0.88 | 0.99 | 0.01 |
| Never Married | 0.96 | 0.03 | 0.16 | 0.90 | 1.02 | . |
| One or Both Had Died | 0.94 | 0.02 | 0.02 | 0.90 | 0.99 | . |
| Childhood Income (Ref: Got By) |  |  |  |  |  |  |
| Lived Comfortably | 1.00 | 0.01 | 0.79 | 0.97 | 1.02 | 0.89 |
| Found it Difficult | 1.00 | 0.01 | 0.98 | 0.97 | 1.03 | . |
| Found it Very Difficult | 0.99 | 0.02 | 0.49 | 0.95 | 1.03 | . |
| Childhood Abuse (Ref: No) |  |  |  |  |  |  |
| Yes | 0.93 | 0.02 | 0.00 | 0.90 | 0.97 | 0.00 |
| Outsider (Ref: No) |  |  |  |  |  |  |
| Yes | 0.98 | 0.02 | 0.27 | 0.94 | 1.02 | 0.27 |
| Childhood Health (Ref: Good) |  |  |  |  |  |  |
| Excellent | 1.03 | 0.02 | 0.06 | 1.00 | 1.06 | 0.19 |
| Very Good | 1.01 | 0.02 | 0.47 | 0.98 | 1.05 | . |
| Fair | 1.02 | 0.02 | 0.32 | 0.98 | 1.07 | . |
| Poor | 1.03 | 0.03 | 0.33 | 0.97 | 1.10 | . |
| Immigration Status (Ref: Born in This Country) |  |  |  |  |  |  |
| Born in Another Country | 0.92 | 0.08 | 0.37 | 0.77 | 1.10 | 0.37 |
| Childhood Service Attendance (Ref: Never) |  |  |  |  |  |  |
| At Least 1/Week | 0.99 | 0.02 | 0.76 | 0.95 | 1.04 | 0.37 |
| 1-3/Month | 1.00 | 0.03 | 0.94 | 0.95 | 1.05 | . |
| <1/Month | 0.93 | 0.04 | 0.09 | 0.86 | 1.01 | . |
| Gender (Ref: Male) |  |  |  |  |  |  |
| Female | 1.04 | 0.01 | 0.00 | 1.02 | 1.06 | 0.00 |
| Other | 1.24 | 0.03 | 0.00 | 1.18 | 1.29 | . |
| Year of Birth (Ref: 1998-2005) |  |  |  |  |  |  |
| 1993-1998; Age 25-29 | 1.01 | 0.01 | 0.39 | 0.99 | 1.03 | 0.00 |
| 1983-1993; Age 30-39 | 1.00 | 0.01 | 0.85 | 0.97 | 1.02 | . |
| 1973-1983; Age 40-49 | 1.05 | 0.02 | 0.01 | 1.01 | 1.08 | . |
| 1963-1973; Age 50-59 | 1.03 | 0.02 | 0.15 | 0.99 | 1.08 | . |
| 1953-1963; Age 60-69 | 1.10 | 0.02 | 0.00 | 1.06 | 1.14 | . |
| 1943-1953; Age 70-79 | 0.98 | 0.08 | 0.78 | 0.84 | 1.15 | . |
| 1943 or Earlier; Age 80 or Older | 1.03 | 0.09 | 0.74 | 0.87 | 1.21 | . |
| Mother Absence/Presence (Ref: Present) |  |  |  |  |  |  |
| Absent | 0.97 | 0.07 | 0.70 | 0.85 | 1.12 | 0.70 |
| Father Absence/Presence (Ref: Present) |  |  |  |  |  |  |
| Absent | 1.01 | 0.04 | 0.82 | 0.93 | 1.09 | 0.82 |
| Childhood Religion (Ref: Christianity) |  |  |  |  |  |  |
| Islam | 1.07 | 0.01 | 0.00 | 1.04 | 1.10 | 0.00 |
| Some Other Religion | 0.96 | 0.11 | 0.73 | 0.76 | 1.21 | . |
| Race/Ethnicity (Ref: Ethnic Plurality) |  |  |  |  |  |  |
| Ethnic Minority | 0.99 | 0.01 | 0.44 | 0.97 | 1.01 | 0.44 |

**Table S13c: E-Values and E-Value Limits (Nigeria)**

| Variable | E-Value | E-Value Limit |
| --- | --- | --- |
| Relationship with Mother (Ref: Very/Somewhat Bad) |  |  |
| Very/Somewhat Good | 1.22 | 1.00 |
| Relationship with Father (Ref: Very/Somewhat Bad) |  |  |
| Very/Somewhat Good | 1.07 | 1.00 |
| Parent Marital Status (Ref: Married) |  |  |
| Divorced | 1.23 | 1.06 |
| Never Married | 1.18 | 1.00 |
| One or Both Had Died | 1.21 | 1.07 |
| Childhood Income (Ref: Got By) |  |  |
| Lived Comfortably | 1.04 | 1.00 |
| Found it Difficult | 1.02 | 1.00 |
| Found it Very Difficult | 1.09 | 1.00 |
| Childhood Abuse (Ref: No) |  |  |
| Yes | 1.23 | 1.15 |
| Outsider (Ref: No) |  |  |
| Yes | 1.12 | 1.00 |
| Childhood Health (Ref: Good) |  |  |
| Excellent | 1.14 | 1.00 |
| Very Good | 1.09 | 1.00 |
| Fair | 1.12 | 1.00 |
| Poor | 1.14 | 1.00 |
| Immigration Status (Ref: Born in This Country) |  |  |
| Born in Another Country | 1.26 | 1.00 |
| Childhood Service Attendance (Ref: Never) |  |  |
| At Least 1/Week | 1.06 | 1.00 |
| 1-3/Month | 1.03 | 1.00 |
| <1/Month | 1.24 | 1.00 |
| Gender (Ref: Male) |  |  |
| Female | 1.17 | 1.12 |
| Other | 1.48 | 1.40 |
| Year of Birth (Ref: 1998-2005) |  |  |
| 1993-1998; Age 25-29 | 1.08 | 1.00 |
| 1983-1993; Age 30-39 | 1.04 | 1.00 |
| 1973-1983; Age 40-49 | 1.18 | 1.09 |
| 1963-1973; Age 50-59 | 1.15 | 1.00 |
| 1953-1963; Age 60-69 | 1.29 | 1.21 |
| 1943-1953; Age 70-79 | 1.12 | 1.00 |
| 1943 or Earlier; Age 80 or Older | 1.14 | 1.00 |
| Mother Absence/Presence (Ref: Present) |  |  |
| Absent | 1.13 | 1.00 |
| Father Absence/Presence (Ref: Present) |  |  |
| Absent | 1.07 | 1.00 |
| Childhood Religion (Ref: Christianity) |  |  |
| Islam | 1.23 | 1.17 |
| Some Other Religion | 1.17 | 1.00 |
| Race/Ethnicity (Ref: Ethnic Plurality) |  |  |
| Ethnic Minority | 1.07 | 1.00 |

**Table S14a: Nationally-Representative Descriptive Statistics of the Observed Sample (Philippines)**

| Variable | Proportion | Frequency |
| --- | --- | --- |
| Relationship with Mother |  |  |
| Very Good | 0.63 | 3333 |
| Somewhat Good | 0.32 | 1703 |
| Somewhat Bad | 0.02 | 124 |
| Very Bad | 0.01 | 39 |
| Not Applicable | 0.01 | 59 |
| Missing | 0.01 | 35 |
| Relationship with Father |  |  |
| Very Good | 0.65 | 3443 |
| Somewhat Good | 0.27 | 1429 |
| Somewhat Bad | 0.03 | 159 |
| Very Bad | 0.01 | 58 |
| Not Applicable | 0.02 | 108 |
| Missing | 0.02 | 95 |
| Parent Marital Status |  |  |
| Married | 0.86 | 4575 |
| Divorced | 0.01 | 64 |
| Never Married | 0.10 | 517 |
| One or Both Had Died | 0.01 | 51 |
| Missing | 0.02 | 86 |
| Childhood Income |  |  |
| Lived Comfortably | 0.18 | 937 |
| Got By | 0.57 | 3006 |
| Found it Difficult | 0.20 | 1055 |
| Found it Very Difficult | 0.06 | 291 |
| Missing | 0.00 | 3 |
| Childhood Abuse |  |  |
| Yes | 0.08 | 420 |
| No | 0.91 | 4837 |
| Missing | 0.01 | 35 |
| Outsider |  |  |
| Yes | 0.07 | 395 |
| No | 0.92 | 4884 |
| Not Applicable | 0.00 | 3 |
| Missing | 0.00 | 9 |
| Childhood Health |  |  |
| Excellent | 0.20 | 1041 |
| Very Good | 0.11 | 559 |
| Good | 0.41 | 2174 |
| Fair | 0.24 | 1246 |
| Poor | 0.05 | 272 |
| Missing | 0.00 | 0 |
| Immigration Status |  |  |
| Born in This Country | 1.00 | 5284 |
| Born in Another Country | 0.00 | 8 |
| Missing | . | . |
| Childhood Service Attendance |  |  |
| At Least 1/Week | 0.46 | 2453 |
| 1-3/Month | 0.32 | 1699 |
| <1/Month | 0.17 | 892 |
| Never | 0.04 | 201 |
| Missing | 0.01 | 47 |
| Gender |  |  |
| Male | 0.50 | 2625 |
| Female | 0.50 | 2643 |
| Other | 0.00 | 13 |
| Missing | 0.00 | 11 |
| Year of Birth |  |  |
| 1998-2005; Age 18-24 | 0.20 | 1073 |
| 1993-1998; Age 25-29 | 0.13 | 695 |
| 1983-1993; Age 30-39 | 0.22 | 1160 |
| 1973-1983; Age 40-49 | 0.18 | 972 |
| 1963-1973; Age 50-59 | 0.14 | 732 |
| 1953-1963; Age 60-69 | 0.09 | 495 |
| 1943-1953; Age 70-79 | 0.03 | 143 |
| 1943 or Earlier; 80 or Older | 0.00 | 23 |
| Missing | . | . |
| Childhood Religion |  |  |
| Christianity | 0.94 | 4968 |
| Islam | 0.05 | 276 |
| Hinduism | . | . |
| Buddhism | 0.00 | 1 |
| Judaism | . | . |
| Sikhism | 0.00 | 4 |
| Baha'i | 0.00 | 1 |
| Jainism | . | . |
| Shinto | . | . |
| Taoism | . | . |
| Confucianism | . | . |
| Primal, Animist, or Folk Religion | 0.00 | 14 |
| Spiritism | . | . |
| African-Derived | . | . |
| Chinese | . | . |
| Some Other Religion | 0.00 | 9 |
| No Religion/Atheist/Agnostic | 0.00 | 9 |
| Missing | 0.00 | 11 |
| Race/Ethnicity |  |  |
| Tagalog | 0.32 | 1691 |
| Cebuana | 0.12 | 656 |
| Ilocano/Ilokano | 0.08 | 429 |
| Visayan/Bisaya | 0.14 | 739 |
| Ilonggo/Hiligaynon | 0.08 | 428 |
| Bicolano/Bikolano | 0.06 | 300 |
| Waray | 0.04 | 216 |
| Tausug | 0.02 | 94 |
| Maranao | 0.01 | 39 |
| Maguindanaoan | 0.02 | 84 |
| Chinese-Filipino | 0.00 | 3 |
| Kapampangan | 0.02 | 107 |
| Pangasinese | 0.02 | 107 |
| Zamboangueno | 0.01 | 51 |
| Malay | . | . |
| Masbateno | 0.01 | 54 |
| Aeta | 0.00 | 1 |
| Igorot | 0.01 | 42 |
| Mangyan | 0.00 | 2 |
| Badjao | 0.00 | 2 |
| Other | 0.05 | 244 |
| Missing | 0.00 | 3 |

**Table S14b: Variations Across Childhood Predictors (Philippines)**

| Variable | IRR | SE | Prob | LCI | UCI | Global p-value |
| --- | --- | --- | --- | --- | --- | --- |
| Relationship with Mother (Ref: Very/Somewhat Bad) |  |  |  |  |  |  |
| Very/Somewhat Good | 1.08 | 0.07 | 0.22 | 0.95 | 1.23 | 0.22 |
| Relationship with Father (Ref: Very/Somewhat Bad) |  |  |  |  |  |  |
| Very/Somewhat Good | 1.03 | 0.06 | 0.58 | 0.93 | 1.15 | 0.58 |
| Parent Marital Status (Ref: Married) |  |  |  |  |  |  |
| Divorced | 1.02 | 0.09 | 0.81 | 0.86 | 1.22 | 0.00 |
| Never Married | 0.93 | 0.04 | 0.10 | 0.85 | 1.01 | . |
| One or Both Had Died | 1.24 | 0.05 | 0.00 | 1.13 | 1.35 | . |
| Childhood Income (Ref: Got By) |  |  |  |  |  |  |
| Lived Comfortably | 1.00 | 0.03 | 0.95 | 0.94 | 1.06 | 0.75 |
| Found it Difficult | 1.03 | 0.03 | 0.30 | 0.98 | 1.08 | . |
| Found it Very Difficult | 1.00 | 0.05 | 0.95 | 0.91 | 1.10 | . |
| Childhood Abuse (Ref: No) |  |  |  |  |  |  |
| Yes | 0.91 | 0.05 | 0.07 | 0.83 | 1.01 | 0.07 |
| Outsider (Ref: No) |  |  |  |  |  |  |
| Yes | 0.93 | 0.05 | 0.12 | 0.84 | 1.02 | 0.12 |
| Childhood Health (Ref: Good) |  |  |  |  |  |  |
| Excellent | 1.08 | 0.03 | 0.00 | 1.03 | 1.14 | 0.04 |
| Very Good | 1.03 | 0.04 | 0.38 | 0.96 | 1.12 | . |
| Fair | 1.00 | 0.03 | 0.92 | 0.94 | 1.06 | . |
| Poor | 1.06 | 0.06 | 0.29 | 0.95 | 1.17 | . |
| Immigration Status (Ref: Born in This Country) |  |  |  |  |  |  |
| Born in Another Country | 0.89 | 0.28 | 0.71 | 0.49 | 1.63 | 0.71 |
| Childhood Service Attendance (Ref: Never) |  |  |  |  |  |  |
| At Least 1/Week | 1.31 | 0.10 | 0.00 | 1.13 | 1.53 | 0.00 |
| 1-3/Month | 1.23 | 0.10 | 0.01 | 1.05 | 1.44 | . |
| <1/Month | 1.09 | 0.09 | 0.33 | 0.92 | 1.28 | . |
| Gender (Ref: Male) |  |  |  |  |  |  |
| Female | 1.15 | 0.02 | 0.00 | 1.10 | 1.19 | 0.00 |
| Other | 1.06 | 0.19 | 0.73 | 0.75 | 1.52 | . |
| Year of Birth (Ref: 1998-2005) |  |  |  |  |  |  |
| 1993-1998; Age 25-29 | 1.10 | 0.05 | 0.05 | 1.00 | 1.20 | 0.00 |
| 1983-1993; Age 30-39 | 1.09 | 0.04 | 0.02 | 1.01 | 1.18 | . |
| 1973-1983; Age 40-49 | 1.14 | 0.04 | 0.00 | 1.06 | 1.23 | . |
| 1963-1973; Age 50-59 | 1.17 | 0.05 | 0.00 | 1.08 | 1.28 | . |
| 1953-1963; Age 60-69 | 1.16 | 0.06 | 0.00 | 1.05 | 1.27 | . |
| 1943-1953; Age 70-79 | 1.26 | 0.08 | 0.00 | 1.12 | 1.43 | . |
| 1943 or Earlier; Age 80 or Older | 1.12 | 0.17 | 0.49 | 0.82 | 1.52 | . |
| Mother Absence/Presence (Ref: Present) |  |  |  |  |  |  |
| Absent | 1.00 | 0.08 | 0.98 | 0.85 | 1.18 | 0.98 |
| Father Absence/Presence (Ref: Present) |  |  |  |  |  |  |
| Absent | 0.97 | 0.07 | 0.64 | 0.84 | 1.12 | 0.64 |
| Childhood Religion (Ref: Christianity) |  |  |  |  |  |  |
| Islam | 0.99 | 0.04 | 0.76 | 0.90 | 1.08 | 0.55 |
| Some Other Religion | 0.82 | 0.15 | 0.29 | 0.57 | 1.19 | . |
| Race/Ethnicity (Ref: Ethnic Plurality) |  |  |  |  |  |  |
| Ethnic Minority | 1.05 | 0.03 | 0.03 | 1.00 | 1.10 | 0.03 |

**Table S14c: E-Values and E-Value Limits (Philippines)**

| Variable | E-Value | E-Value Limit |
| --- | --- | --- |
| Relationship with Mother (Ref: Very/Somewhat Bad) |  |  |
| Very/Somewhat Good | 1.27 | 1.00 |
| Relationship with Father (Ref: Very/Somewhat Bad) |  |  |
| Very/Somewhat Good | 1.15 | 1.00 |
| Parent Marital Status (Ref: Married) |  |  |
| Divorced | 1.13 | 1.00 |
| Never Married | 1.26 | 1.00 |
| One or Both Had Died | 1.52 | 1.33 |
| Childhood Income (Ref: Got By) |  |  |
| Lived Comfortably | 1.03 | 1.00 |
| Found it Difficult | 1.14 | 1.00 |
| Found it Very Difficult | 1.04 | 1.00 |
| Childhood Abuse (Ref: No) |  |  |
| Yes | 1.29 | 1.00 |
| Outsider (Ref: No) |  |  |
| Yes | 1.26 | 1.00 |
| Childhood Health (Ref: Good) |  |  |
| Excellent | 1.27 | 1.13 |
| Very Good | 1.16 | 1.00 |
| Fair | 1.04 | 1.00 |
| Poor | 1.22 | 1.00 |
| Immigration Status (Ref: Born in This Country) |  |  |
| Born in Another Country | 1.35 | 1.00 |
| Childhood Service Attendance (Ref: Never) |  |  |
| At Least 1/Week | 1.63 | 1.32 |
| 1-3/Month | 1.51 | 1.18 |
| <1/Month | 1.28 | 1.00 |
| Gender (Ref: Male) |  |  |
| Female | 1.39 | 1.28 |
| Other | 1.23 | 1.00 |
| Year of Birth (Ref: 1998-2005) |  |  |
| 1993-1998; Age 25-29 | 1.30 | 1.00 |
| 1983-1993; Age 30-39 | 1.30 | 1.09 |
| 1973-1983; Age 40-49 | 1.38 | 1.20 |
| 1963-1973; Age 50-59 | 1.43 | 1.24 |
| 1953-1963; Age 60-69 | 1.40 | 1.19 |
| 1943-1953; Age 70-79 | 1.56 | 1.30 |
| 1943 or Earlier; Age 80 or Older | 1.33 | 1.00 |
| Mother Absence/Presence (Ref: Present) |  |  |
| Absent | 1.04 | 1.00 |
| Father Absence/Presence (Ref: Present) |  |  |
| Absent | 1.16 | 1.00 |
| Childhood Religion (Ref: Christianity) |  |  |
| Islam | 1.10 | 1.00 |
| Some Other Religion | 1.50 | 1.00 |
| Race/Ethnicity (Ref: Ethnic Plurality) |  |  |
| Ethnic Minority | 1.21 | 1.05 |

**Table S15a: Nationally-Representative Descriptive Statistics of the Observed Sample (Poland)**

| Variable | Proportion | Frequency |
| --- | --- | --- |
| Relationship with Mother |  |  |
| Very Good | 0.47 | 4879 |
| Somewhat Good | 0.48 | 4973 |
| Somewhat Bad | 0.03 | 285 |
| Very Bad | 0.01 | 58 |
| Not Applicable | 0.01 | 80 |
| Missing | 0.01 | 112 |
| Relationship with Father |  |  |
| Very Good | 0.41 | 4231 |
| Somewhat Good | 0.48 | 4984 |
| Somewhat Bad | 0.05 | 516 |
| Very Bad | 0.01 | 78 |
| Not Applicable | 0.04 | 407 |
| Missing | 0.02 | 173 |
| Parent Marital Status |  |  |
| Married | 0.86 | 8972 |
| Divorced | 0.06 | 587 |
| Never Married | 0.02 | 193 |
| One or Both Had Died | 0.03 | 313 |
| Missing | 0.03 | 324 |
| Childhood Income |  |  |
| Lived Comfortably | 0.13 | 1384 |
| Got By | 0.60 | 6257 |
| Found it Difficult | 0.21 | 2133 |
| Found it Very Difficult | 0.05 | 509 |
| Missing | 0.01 | 106 |
| Childhood Abuse |  |  |
| Yes | 0.03 | 325 |
| No | 0.96 | 10009 |
| Missing | 0.01 | 55 |
| Outsider |  |  |
| Yes | 0.05 | 490 |
| No | 0.93 | 9615 |
| Not Applicable | 0.00 | 33 |
| Missing | 0.02 | 252 |
| Childhood Health |  |  |
| Excellent | 0.26 | 2676 |
| Very Good | 0.52 | 5371 |
| Good | 0.17 | 1779 |
| Fair | 0.04 | 406 |
| Poor | 0.01 | 123 |
| Missing | 0.00 | 34 |
| Immigration Status |  |  |
| Born in This Country | 0.99 | 10258 |
| Born in Another Country | 0.01 | 108 |
| Missing | 0.00 | 23 |
| Childhood Service Attendance |  |  |
| At Least 1/Week | 0.46 | 4751 |
| 1-3/Month | 0.26 | 2689 |
| <1/Month | 0.21 | 2161 |
| Never | 0.03 | 354 |
| Missing | 0.04 | 434 |
| Gender |  |  |
| Male | 0.48 | 4974 |
| Female | 0.52 | 5387 |
| Other | 0.00 | 3 |
| Missing | 0.00 | 26 |
| Year of Birth |  |  |
| 1998-2005; Age 18-24 | 0.09 | 955 |
| 1993-1998; Age 25-29 | 0.07 | 761 |
| 1983-1993; Age 30-39 | 0.21 | 2159 |
| 1973-1983; Age 40-49 | 0.19 | 1956 |
| 1963-1973; Age 50-59 | 0.16 | 1670 |
| 1953-1963; Age 60-69 | 0.18 | 1909 |
| 1943-1953; Age 70-79 | 0.08 | 833 |
| 1943 or Earlier; 80 or Older | 0.01 | 145 |
| Missing | 0.00 | 1 |
| Childhood Religion |  |  |
| Christianity | 0.95 | 9861 |
| Islam | 0.00 | 3 |
| Hinduism | . | . |
| Buddhism | 0.00 | 2 |
| Judaism | . | . |
| Sikhism | 0.00 | 1 |
| Baha'i | . | . |
| Jainism | . | . |
| Shinto | . | . |
| Taoism | . | . |
| Confucianism | . | . |
| Primal, Animist, or Folk Religion | 0.00 | 5 |
| Spiritism | . | . |
| African-Derived | . | . |
| Chinese | . | . |
| Some Other Religion | . | . |
| No Religion/Atheist/Agnostic | 0.05 | 482 |
| Missing | 0.00 | 35 |
| Race/Ethnicity |  |  |
| Polish | 0.99 | 10309 |
| German | 0.00 | 4 |
| Belarussian | 0.00 | 2 |
| Ukranian | 0.00 | 38 |
| Roma | . | . |
| Russian | . | . |
| Ethnic Jewish | . | . |
| Lemko | . | . |
| Silesia | 0.00 | 14 |
| Kashubians | 0.00 | 3 |
| Other | 0.00 | 4 |
| Missing | 0.00 | 14 |

**Table S15b: Variations Across Childhood Predictors (Poland)**

| Variable | IRR | SE | Prob | LCI | UCI | Global p-value |
| --- | --- | --- | --- | --- | --- | --- |
| Relationship with Mother (Ref: Very/Somewhat Bad) |  |  |  |  |  |  |
| Very/Somewhat Good | 1.06 | 0.17 | 0.72 | 0.77 | 1.45 | 0.72 |
| Relationship with Father (Ref: Very/Somewhat Bad) |  |  |  |  |  |  |
| Very/Somewhat Good | 1.31 | 0.17 | 0.04 | 1.02 | 1.68 | 0.04 |
| Parent Marital Status (Ref: Married) |  |  |  |  |  |  |
| Divorced | 1.05 | 0.12 | 0.67 | 0.84 | 1.32 | 0.06 |
| Never Married | 1.39 | 0.18 | 0.01 | 1.09 | 1.78 | . |
| One or Both Had Died | 0.91 | 0.15 | 0.57 | 0.65 | 1.27 | . |
| Childhood Income (Ref: Got By) |  |  |  |  |  |  |
| Lived Comfortably | 0.87 | 0.08 | 0.11 | 0.73 | 1.03 | 0.05 |
| Found it Difficult | 1.11 | 0.06 | 0.05 | 1.00 | 1.23 | . |
| Found it Very Difficult | 1.00 | 0.10 | 0.99 | 0.82 | 1.22 | . |
| Childhood Abuse (Ref: No) |  |  |  |  |  |  |
| Yes | 0.84 | 0.12 | 0.21 | 0.64 | 1.10 | 0.21 |
| Outsider (Ref: No) |  |  |  |  |  |  |
| Yes | 1.16 | 0.13 | 0.17 | 0.94 | 1.44 | 0.17 |
| Childhood Health (Ref: Good) |  |  |  |  |  |  |
| Excellent | 0.81 | 0.06 | 0.00 | 0.70 | 0.94 | 0.04 |
| Very Good | 0.95 | 0.06 | 0.41 | 0.83 | 1.08 | . |
| Fair | 0.95 | 0.11 | 0.65 | 0.76 | 1.19 | . |
| Poor | 1.12 | 0.16 | 0.42 | 0.85 | 1.50 | . |
| Immigration Status (Ref: Born in This Country) |  |  |  |  |  |  |
| Born in Another Country | 1.17 | 0.30 | 0.55 | 0.69 | 1.96 | 0.55 |
| Childhood Service Attendance (Ref: Never) |  |  |  |  |  |  |
| At Least 1/Week | 3.56 | 0.78 | 0.00 | 2.31 | 5.50 | 0.00 |
| 1-3/Month | 2.31 | 0.52 | 0.00 | 1.48 | 3.62 | . |
| <1/Month | 1.19 | 0.30 | 0.48 | 0.73 | 1.95 | . |
| Gender (Ref: Male) |  |  |  |  |  |  |
| Female | 1.49 | 0.08 | 0.00 | 1.35 | 1.64 | 0.00 |
| Other | 3.15 | 1.75 | 0.04 | 1.05 | 9.39 | . |
| Year of Birth (Ref: 1998-2005) |  |  |  |  |  |  |
| 1993-1998; Age 25-29 | 1.01 | 0.15 | 0.95 | 0.76 | 1.34 | 0.00 |
| 1983-1993; Age 30-39 | 1.30 | 0.17 | 0.05 | 1.00 | 1.69 | . |
| 1973-1983; Age 40-49 | 1.46 | 0.19 | 0.00 | 1.13 | 1.89 | . |
| 1963-1973; Age 50-59 | 1.98 | 0.26 | 0.00 | 1.52 | 2.57 | . |
| 1953-1963; Age 60-69 | 2.94 | 0.42 | 0.00 | 2.23 | 3.88 | . |
| 1943-1953; Age 70-79 | 3.28 | 0.48 | 0.00 | 2.46 | 4.37 | . |
| 1943 or Earlier; Age 80 or Older | 2.92 | 0.59 | 0.00 | 1.98 | 4.33 | . |
| Mother Absence/Presence (Ref: Present) |  |  |  |  |  |  |
| Absent | 0.96 | 0.30 | 0.90 | 0.52 | 1.79 | 0.90 |
| Father Absence/Presence (Ref: Present) |  |  |  |  |  |  |
| Absent | 1.31 | 0.21 | 0.10 | 0.95 | 1.81 | 0.10 |
| Childhood Religion (Ref: No Religion/Atheist/Agnostic) |  |  |  |  |  |  |
| Christianity | 0.65 | 0.11 | 0.01 | 0.47 | 0.91 | 0.02 |
| Some Other Religion | 1.11 | 0.57 | 0.84 | 0.41 | 3.06 | . |
| Race/Ethnicity (Ref: Ethnic Plurality) |  |  |  |  |  |  |
| Ethnic Minority | 0.90 | 0.30 | 0.74 | 0.47 | 1.71 | 0.74 |

**Table S15c: E-Values and E-Value Limits (Poland)**

| Variable | E-Value | E-Value Limit |
| --- | --- | --- |
| Relationship with Mother (Ref: Very/Somewhat Bad) |  |  |
| Very/Somewhat Good | 1.30 | 1.00 |
| Relationship with Father (Ref: Very/Somewhat Bad) |  |  |
| Very/Somewhat Good | 1.89 | 1.10 |
| Parent Marital Status (Ref: Married) |  |  |
| Divorced | 1.27 | 1.00 |
| Never Married | 2.07 | 1.25 |
| One or Both Had Died | 1.41 | 1.00 |
| Childhood Income (Ref: Got By) |  |  |
| Lived Comfortably | 1.54 | 1.00 |
| Found it Difficult | 1.44 | 1.00 |
| Found it Very Difficult | 1.03 | 1.00 |
| Childhood Abuse (Ref: No) |  |  |
| Yes | 1.64 | 1.00 |
| Outsider (Ref: No) |  |  |
| Yes | 1.57 | 1.00 |
| Childhood Health (Ref: Good) |  |  |
| Excellent | 1.73 | 1.22 |
| Very Good | 1.29 | 1.00 |
| Fair | 1.28 | 1.00 |
| Poor | 1.48 | 1.00 |
| Immigration Status (Ref: Born in This Country) |  |  |
| Born in Another Country | 1.58 | 1.00 |
| Childhood Service Attendance (Ref: Never) |  |  |
| At Least 1/Week | 6.04 | 2.41 |
| 1-3/Month | 3.82 | 1.73 |
| <1/Month | 1.64 | 1.00 |
| Gender (Ref: Male) |  |  |
| Female | 2.26 | 1.59 |
| Other | 5.31 | 1.19 |
| Year of Birth (Ref: 1998-2005) |  |  |
| 1993-1998; Age 25-29 | 1.10 | 1.00 |
| 1983-1993; Age 30-39 | 1.87 | 1.01 |
| 1973-1983; Age 40-49 | 2.21 | 1.33 |
| 1963-1973; Age 50-59 | 3.20 | 1.77 |
| 1953-1963; Age 60-69 | 4.95 | 2.35 |
| 1943-1953; Age 70-79 | 5.55 | 2.51 |
| 1943 or Earlier; Age 80 or Older | 4.92 | 2.16 |
| Mother Absence/Presence (Ref: Present) |  |  |
| Absent | 1.23 | 1.00 |
| Father Absence/Presence (Ref: Present) |  |  |
| Absent | 1.90 | 1.00 |
| Childhood Religion (Ref: No Religion/Atheist/Agnostic) |  |  |
| Christianity | 2.36 | 1.28 |
| Some Other Religion | 1.45 | 1.00 |
| Race/Ethnicity (Ref: Ethnic Plurality) |  |  |
| Ethnic Minority | 1.46 | 1.00 |

**Table S16a: Nationally-Representative Descriptive Statistics of the Observed Sample (South Africa)**

| Variable | Proportion | Frequency |
| --- | --- | --- |
| Relationship with Mother |  |  |
| Very Good | 0.82 | 2186 |
| Somewhat Good | 0.10 | 263 |
| Somewhat Bad | 0.02 | 51 |
| Very Bad | 0.01 | 39 |
| Not Applicable | 0.03 | 90 |
| Missing | 0.01 | 21 |
| Relationship with Father |  |  |
| Very Good | 0.62 | 1656 |
| Somewhat Good | 0.13 | 333 |
| Somewhat Bad | 0.03 | 86 |
| Very Bad | 0.06 | 159 |
| Not Applicable | 0.12 | 331 |
| Missing | 0.03 | 85 |
| Parent Marital Status |  |  |
| Married | 0.50 | 1321 |
| Divorced | 0.05 | 131 |
| Never Married | 0.34 | 904 |
| One or Both Had Died | 0.05 | 140 |
| Missing | 0.06 | 155 |
| Childhood Income |  |  |
| Lived Comfortably | 0.40 | 1050 |
| Got By | 0.33 | 875 |
| Found it Difficult | 0.16 | 432 |
| Found it Very Difficult | 0.11 | 289 |
| Missing | 0.00 | 5 |
| Childhood Abuse |  |  |
| Yes | 0.17 | 450 |
| No | 0.81 | 2149 |
| Missing | 0.02 | 52 |
| Outsider |  |  |
| Yes | 0.16 | 434 |
| No | 0.83 | 2211 |
| Not Applicable | 0.00 | 3 |
| Missing | 0.00 | 3 |
| Childhood Health |  |  |
| Excellent | 0.46 | 1225 |
| Very Good | 0.22 | 590 |
| Good | 0.14 | 370 |
| Fair | 0.10 | 266 |
| Poor | 0.07 | 183 |
| Missing | 0.01 | 17 |
| Immigration Status |  |  |
| Born in This Country | 0.95 | 2511 |
| Born in Another Country | 0.05 | 139 |
| Missing | 0.00 | 1 |
| Childhood Service Attendance |  |  |
| At Least 1/Week | 0.63 | 1681 |
| 1-3/Month | 0.21 | 552 |
| <1/Month | 0.07 | 175 |
| Never | 0.08 | 217 |
| Missing | 0.01 | 26 |
| Gender |  |  |
| Male | 0.49 | 1288 |
| Female | 0.51 | 1356 |
| Other | 0.00 | 2 |
| Missing | 0.00 | 4 |
| Year of Birth |  |  |
| 1998-2005; Age 18-24 | 0.17 | 461 |
| 1993-1998; Age 25-29 | 0.14 | 364 |
| 1983-1993; Age 30-39 | 0.25 | 655 |
| 1973-1983; Age 40-49 | 0.20 | 522 |
| 1963-1973; Age 50-59 | 0.12 | 309 |
| 1953-1963; Age 60-69 | 0.07 | 195 |
| 1943-1953; Age 70-79 | 0.05 | 120 |
| 1943 or Earlier; 80 or Older | 0.01 | 17 |
| Missing | 0.00 | 9 |
| Childhood Religion |  |  |
| Christianity | 0.88 | 2323 |
| Islam | 0.02 | 52 |
| Hinduism | 0.00 | 2 |
| Buddhism | 0.00 | 11 |
| Judaism | . | . |
| Sikhism | . | . |
| Baha'i | . | . |
| Jainism | . | . |
| Shinto | 0.00 | 2 |
| Taoism | 0.00 | 1 |
| Confucianism | . | . |
| Primal, Animist, or Folk Religion | 0.04 | 117 |
| Spiritism | . | . |
| African-Derived | . | . |
| Chinese | . | . |
| Some Other Religion | 0.00 | 7 |
| No Religion/Atheist/Agnostic | 0.04 | 107 |
| Missing | 0.01 | 27 |
| Race/Ethnicity |  |  |
| Black | 0.90 | 2381 |
| Asian/Indian | 0.00 | 6 |
| Colored | 0.10 | 252 |
| White | 0.00 | 8 |
| Other | 0.00 | 0 |
| Missing | 0.00 | 3 |

**Table S16b: Variations Across Childhood Predictors (South Africa)**

| Variable | IRR | SE | Prob | LCI | UCI | Global p-value |
| --- | --- | --- | --- | --- | --- | --- |
| Relationship with Mother (Ref: Very/Somewhat Bad) |  |  |  |  |  |  |
| Very/Somewhat Good | 0.92 | 0.07 | 0.23 | 0.79 | 1.06 | 0.23 |
| Relationship with Father (Ref: Very/Somewhat Bad) |  |  |  |  |  |  |
| Very/Somewhat Good | 1.01 | 0.05 | 0.89 | 0.91 | 1.12 | 0.89 |
| Parent Marital Status (Ref: Married) |  |  |  |  |  |  |
| Divorced | 0.89 | 0.08 | 0.17 | 0.75 | 1.05 | 0.45 |
| Never Married | 1.02 | 0.04 | 0.68 | 0.94 | 1.10 | . |
| One or Both Had Died | 0.98 | 0.08 | 0.84 | 0.85 | 1.15 | . |
| Childhood Income (Ref: Got By) |  |  |  |  |  |  |
| Lived Comfortably | 1.08 | 0.04 | 0.03 | 1.01 | 1.16 | 0.19 |
| Found it Difficult | 1.04 | 0.05 | 0.36 | 0.95 | 1.14 | . |
| Found it Very Difficult | 1.05 | 0.06 | 0.40 | 0.94 | 1.18 | . |
| Childhood Abuse (Ref: No) |  |  |  |  |  |  |
| Yes | 0.98 | 0.05 | 0.66 | 0.89 | 1.07 | 0.66 |
| Outsider (Ref: No) |  |  |  |  |  |  |
| Yes | 1.02 | 0.04 | 0.54 | 0.95 | 1.11 | 0.54 |
| Childhood Health (Ref: Good) |  |  |  |  |  |  |
| Excellent | 0.96 | 0.04 | 0.35 | 0.88 | 1.05 | 0.69 |
| Very Good | 0.96 | 0.05 | 0.43 | 0.86 | 1.07 | . |
| Fair | 0.98 | 0.06 | 0.71 | 0.86 | 1.10 | . |
| Poor | 1.03 | 0.07 | 0.70 | 0.89 | 1.18 | . |
| Immigration Status (Ref: Born in This Country) |  |  |  |  |  |  |
| Born in Another Country | 0.90 | 0.10 | 0.36 | 0.72 | 1.13 | 0.36 |
| Childhood Service Attendance (Ref: Never) |  |  |  |  |  |  |
| At Least 1/Week | 1.11 | 0.12 | 0.34 | 0.89 | 1.38 | 0.15 |
| 1-3/Month | 1.15 | 0.13 | 0.21 | 0.92 | 1.45 | . |
| <1/Month | 0.98 | 0.12 | 0.85 | 0.76 | 1.25 | . |
| Gender (Ref: Male) |  |  |  |  |  |  |
| Female | 1.14 | 0.04 | 0.00 | 1.07 | 1.22 | 0.00 |
| Other | 1.65 | 0.12 | 0.00 | 1.42 | 1.91 | . |
| Year of Birth (Ref: 1998-2005) |  |  |  |  |  |  |
| 1993-1998; Age 25-29 | 0.95 | 0.05 | 0.33 | 0.86 | 1.05 | 0.00 |
| 1983-1993; Age 30-39 | 0.95 | 0.04 | 0.25 | 0.87 | 1.04 | . |
| 1973-1983; Age 40-49 | 0.99 | 0.06 | 0.89 | 0.89 | 1.11 | . |
| 1963-1973; Age 50-59 | 1.13 | 0.07 | 0.05 | 1.00 | 1.28 | . |
| 1953-1963; Age 60-69 | 1.15 | 0.08 | 0.04 | 1.01 | 1.31 | . |
| 1943-1953; Age 70-79 | 1.25 | 0.12 | 0.02 | 1.04 | 1.50 | . |
| 1943 or Earlier; Age 80 or Older | 1.33 | 0.07 | 0.00 | 1.19 | 1.48 | . |
| Mother Absence/Presence (Ref: Present) |  |  |  |  |  |  |
| Absent | 0.89 | 0.08 | 0.19 | 0.74 | 1.06 | 0.19 |
| Father Absence/Presence (Ref: Present) |  |  |  |  |  |  |
| Absent | 1.03 | 0.06 | 0.59 | 0.92 | 1.16 | 0.59 |
| Childhood Religion (Ref: No Religion/Atheist/Agnostic) |  |  |  |  |  |  |
| Christianity | 1.19 | 0.21 | 0.34 | 0.84 | 1.68 | 0.57 |
| Primal, Animist, or Folk Religion | 1.10 | 0.21 | 0.60 | 0.76 | 1.59 | . |
| Some Other Religion | 1.09 | 0.23 | 0.69 | 0.71 | 1.66 | . |
| Race/Ethnicity (Ref: Ethnic Plurality) |  |  |  |  |  |  |
| Ethnic Minority | 0.93 | 0.07 | 0.35 | 0.80 | 1.08 | 0.35 |

**Table S16c: E-Values and E-Value Limits (South Africa)**

| Variable | E-Value | E-Value Limit |
| --- | --- | --- |
| Relationship with Mother (Ref: Very/Somewhat Bad) |  |  |
| Very/Somewhat Good | 1.29 | 1.00 |
| Relationship with Father (Ref: Very/Somewhat Bad) |  |  |
| Very/Somewhat Good | 1.07 | 1.00 |
| Parent Marital Status (Ref: Married) |  |  |
| Divorced | 1.35 | 1.00 |
| Never Married | 1.11 | 1.00 |
| One or Both Had Died | 1.10 | 1.00 |
| Childhood Income (Ref: Got By) |  |  |
| Lived Comfortably | 1.27 | 1.07 |
| Found it Difficult | 1.18 | 1.00 |
| Found it Very Difficult | 1.21 | 1.00 |
| Childhood Abuse (Ref: No) |  |  |
| Yes | 1.12 | 1.00 |
| Outsider (Ref: No) |  |  |
| Yes | 1.14 | 1.00 |
| Childhood Health (Ref: Good) |  |  |
| Excellent | 1.19 | 1.00 |
| Very Good | 1.19 | 1.00 |
| Fair | 1.13 | 1.00 |
| Poor | 1.14 | 1.00 |
| Immigration Status (Ref: Born in This Country) |  |  |
| Born in Another Country | 1.33 | 1.00 |
| Childhood Service Attendance (Ref: Never) |  |  |
| At Least 1/Week | 1.33 | 1.00 |
| 1-3/Month | 1.40 | 1.00 |
| <1/Month | 1.13 | 1.00 |
| Gender (Ref: Male) |  |  |
| Female | 1.38 | 1.22 |
| Other | 2.03 | 1.67 |
| Year of Birth (Ref: 1998-2005) |  |  |
| 1993-1998; Age 25-29 | 1.20 | 1.00 |
| 1983-1993; Age 30-39 | 1.21 | 1.00 |
| 1973-1983; Age 40-49 | 1.07 | 1.00 |
| 1963-1973; Age 50-59 | 1.36 | 1.03 |
| 1953-1963; Age 60-69 | 1.39 | 1.06 |
| 1943-1953; Age 70-79 | 1.55 | 1.17 |
| 1943 or Earlier; Age 80 or Older | 1.65 | 1.40 |
| Mother Absence/Presence (Ref: Present) |  |  |
| Absent | 1.36 | 1.00 |
| Father Absence/Presence (Ref: Present) |  |  |
| Absent | 1.16 | 1.00 |
| Childhood Religion (Ref: No Religion/Atheist/Agnostic) |  |  |
| Christianity | 1.45 | 1.00 |
| Primal, Animist, or Folk Religion | 1.31 | 1.00 |
| Some Other Religion | 1.28 | 1.00 |
| Race/Ethnicity (Ref: Ethnic Plurality) |  |  |
| Ethnic Minority | 1.25 | 1.00 |

**Table S17a: Nationally-Representative Descriptive Statistics of the Observed Sample (Spain)**

| Variable | Proportion | Frequency |
| --- | --- | --- |
| Relationship with Mother |  |  |
| Very Good | 0.72 | 4557 |
| Somewhat Good | 0.20 | 1258 |
| Somewhat Bad | 0.04 | 248 |
| Very Bad | 0.01 | 92 |
| Not Applicable | 0.02 | 107 |
| Missing | 0.00 | 28 |
| Relationship with Father |  |  |
| Very Good | 0.66 | 4131 |
| Somewhat Good | 0.22 | 1397 |
| Somewhat Bad | 0.05 | 309 |
| Very Bad | 0.03 | 178 |
| Not Applicable | 0.04 | 243 |
| Missing | 0.01 | 33 |
| Parent Marital Status |  |  |
| Married | 0.84 | 5285 |
| Divorced | 0.06 | 378 |
| Never Married | 0.05 | 312 |
| One or Both Had Died | 0.02 | 126 |
| Missing | 0.03 | 188 |
| Childhood Income |  |  |
| Lived Comfortably | 0.32 | 2041 |
| Got By | 0.47 | 2956 |
| Found it Difficult | 0.18 | 1154 |
| Found it Very Difficult | 0.02 | 110 |
| Missing | 0.00 | 29 |
| Childhood Abuse |  |  |
| Yes | 0.10 | 659 |
| No | 0.88 | 5510 |
| Missing | 0.02 | 122 |
| Outsider |  |  |
| Yes | 0.09 | 579 |
| No | 0.90 | 5637 |
| Not Applicable | 0.01 | 36 |
| Missing | 0.01 | 39 |
| Childhood Health |  |  |
| Excellent | 0.39 | 2450 |
| Very Good | 0.36 | 2286 |
| Good | 0.20 | 1235 |
| Fair | 0.03 | 164 |
| Poor | 0.02 | 135 |
| Missing | 0.00 | 20 |
| Immigration Status |  |  |
| Born in This Country | 0.87 | 5479 |
| Born in Another Country | 0.13 | 788 |
| Missing | 0.00 | 23 |
| Childhood Service Attendance |  |  |
| At Least 1/Week | 0.38 | 2391 |
| 1-3/Month | 0.18 | 1132 |
| <1/Month | 0.20 | 1287 |
| Never | 0.23 | 1445 |
| Missing | 0.01 | 36 |
| Gender |  |  |
| Male | 0.50 | 3142 |
| Female | 0.50 | 3119 |
| Other | 0.00 | 6 |
| Missing | 0.00 | 22 |
| Year of Birth |  |  |
| 1998-2005; Age 18-24 | 0.09 | 594 |
| 1993-1998; Age 25-29 | 0.07 | 450 |
| 1983-1993; Age 30-39 | 0.18 | 1111 |
| 1973-1983; Age 40-49 | 0.22 | 1396 |
| 1963-1973; Age 50-59 | 0.20 | 1252 |
| 1953-1963; Age 60-69 | 0.16 | 977 |
| 1943-1953; Age 70-79 | 0.07 | 467 |
| 1943 or Earlier; 80 or Older | 0.01 | 43 |
| Missing | . | . |
| Childhood Religion |  |  |
| Christianity | 0.81 | 5119 |
| Islam | 0.02 | 132 |
| Hinduism | 0.00 | 5 |
| Buddhism | 0.00 | 8 |
| Judaism | 0.00 | 5 |
| Sikhism | 0.00 | 2 |
| Baha'i | . | . |
| Jainism | . | . |
| Shinto | . | . |
| Taoism | . | . |
| Confucianism | 0.00 | 1 |
| Primal, Animist, or Folk Religion | 0.00 | 4 |
| Spiritism | . | . |
| African-Derived | . | . |
| Chinese | . | . |
| Some Other Religion | 0.00 | 13 |
| No Religion/Atheist/Agnostic | 0.15 | 972 |
| Missing | 0.00 | 29 |
| Race/Ethnicity |  |  |
| No Data | . | . |

**Table S17b: Variations Across Childhood Predictors (Spain)**

| Variable | IRR | SE | Prob | LCI | UCI | Global p-value |
| --- | --- | --- | --- | --- | --- | --- |
| Relationship with Mother (Ref: Very/Somewhat Bad) |  |  |  |  |  |  |
| Very/Somewhat Good | 1.06 | 0.13 | 0.63 | 0.84 | 1.34 | 0.63 |
| Relationship with Father (Ref: Very/Somewhat Bad) |  |  |  |  |  |  |
| Very/Somewhat Good | 1.17 | 0.11 | 0.10 | 0.97 | 1.42 | 0.10 |
| Parent Marital Status (Ref: Married) |  |  |  |  |  |  |
| Divorced | 1.43 | 0.13 | 0.00 | 1.20 | 1.70 | 0.00 |
| Never Married | 1.25 | 0.11 | 0.02 | 1.04 | 1.49 | . |
| One or Both Had Died | 0.88 | 0.18 | 0.53 | 0.58 | 1.32 | . |
| Childhood Income (Ref: Got By) |  |  |  |  |  |  |
| Lived Comfortably | 1.09 | 0.07 | 0.17 | 0.96 | 1.24 | 0.32 |
| Found it Difficult | 1.02 | 0.08 | 0.79 | 0.87 | 1.20 | . |
| Found it Very Difficult | 0.78 | 0.17 | 0.28 | 0.51 | 1.21 | . |
| Childhood Abuse (Ref: No) |  |  |  |  |  |  |
| Yes | 1.05 | 0.08 | 0.58 | 0.89 | 1.22 | 0.58 |
| Outsider (Ref: No) |  |  |  |  |  |  |
| Yes | 1.25 | 0.10 | 0.00 | 1.07 | 1.45 | 0.00 |
| Childhood Health (Ref: Good) |  |  |  |  |  |  |
| Excellent | 1.21 | 0.10 | 0.02 | 1.03 | 1.43 | 0.03 |
| Very Good | 1.31 | 0.11 | 0.00 | 1.11 | 1.55 | . |
| Fair | 1.14 | 0.23 | 0.53 | 0.76 | 1.69 | . |
| Poor | 1.21 | 0.23 | 0.32 | 0.83 | 1.74 | . |
| Immigration Status (Ref: Born in This Country) |  |  |  |  |  |  |
| Born in Another Country | 1.63 | 0.10 | 0.00 | 1.45 | 1.83 | 0.00 |
| Childhood Service Attendance (Ref: Never) |  |  |  |  |  |  |
| At Least 1/Week | 2.82 | 0.29 | 0.00 | 2.31 | 3.45 | 0.00 |
| 1-3/Month | 2.40 | 0.26 | 0.00 | 1.94 | 2.98 | . |
| <1/Month | 1.71 | 0.20 | 0.00 | 1.35 | 2.15 | . |
| Gender (Ref: Male) |  |  |  |  |  |  |
| Female | 1.01 | 0.06 | 0.83 | 0.91 | 1.13 | 0.14 |
| Other | 1.64 | 0.41 | 0.05 | 1.00 | 2.68 | . |
| Year of Birth (Ref: 1998-2005) |  |  |  |  |  |  |
| 1993-1998; Age 25-29 | 1.02 | 0.12 | 0.90 | 0.81 | 1.27 | 0.05 |
| 1983-1993; Age 30-39 | 0.98 | 0.10 | 0.88 | 0.81 | 1.19 | . |
| 1973-1983; Age 40-49 | 0.93 | 0.09 | 0.44 | 0.77 | 1.12 | . |
| 1963-1973; Age 50-59 | 0.94 | 0.10 | 0.54 | 0.76 | 1.15 | . |
| 1953-1963; Age 60-69 | 0.99 | 0.12 | 0.95 | 0.78 | 1.26 | . |
| 1943-1953; Age 70-79 | 1.38 | 0.20 | 0.02 | 1.05 | 1.83 | . |
| 1943 or Earlier; Age 80 or Older | 1.66 | 0.51 | 0.10 | 0.91 | 3.04 | . |
| Mother Absence/Presence (Ref: Present) |  |  |  |  |  |  |
| Absent | 1.06 | 0.15 | 0.69 | 0.80 | 1.40 | 0.69 |
| Father Absence/Presence (Ref: Present) |  |  |  |  |  |  |
| Absent | 1.15 | 0.13 | 0.19 | 0.93 | 1.44 | 0.19 |
| Childhood Religion (Ref: No Religion/Atheist/Agnostic) |  |  |  |  |  |  |
| Christianity | 1.58 | 0.18 | 0.00 | 1.26 | 1.98 | 0.00 |
| Some Other Religion | 2.49 | 0.37 | 0.00 | 1.86 | 3.33 | . |

**Table S17c: E-Values and E-Value Limits (Spain)**

| Variable | E-Value | E-Value Limit |
| --- | --- | --- |
| Relationship with Mother (Ref: Very/Somewhat Bad) |  |  |
| Very/Somewhat Good | 1.30 | 1.00 |
| Relationship with Father (Ref: Very/Somewhat Bad) |  |  |
| Very/Somewhat Good | 1.61 | 1.00 |
| Parent Marital Status (Ref: Married) |  |  |
| Divorced | 2.19 | 1.42 |
| Never Married | 1.78 | 1.17 |
| One or Both Had Died | 1.53 | 1.00 |
| Childhood Income (Ref: Got By) |  |  |
| Lived Comfortably | 1.40 | 1.00 |
| Found it Difficult | 1.17 | 1.00 |
| Found it Very Difficult | 1.85 | 1.00 |
| Childhood Abuse (Ref: No) |  |  |
| Yes | 1.26 | 1.00 |
| Outsider (Ref: No) |  |  |
| Yes | 1.79 | 1.23 |
| Childhood Health (Ref: Good) |  |  |
| Excellent | 1.70 | 1.13 |
| Very Good | 1.94 | 1.30 |
| Fair | 1.52 | 1.00 |
| Poor | 1.69 | 1.00 |
| Immigration Status (Ref: Born in This Country) |  |  |
| Born in Another Country | 2.61 | 1.70 |
| Childhood Service Attendance (Ref: Never) |  |  |
| At Least 1/Week | 4.96 | 2.41 |
| 1-3/Month | 4.15 | 2.13 |
| <1/Month | 2.76 | 1.60 |
| Gender (Ref: Male) |  |  |
| Female | 1.12 | 1.00 |
| Other | 2.63 | 1.05 |
| Year of Birth (Ref: 1998-2005) |  |  |
| 1993-1998; Age 25-29 | 1.14 | 1.00 |
| 1983-1993; Age 30-39 | 1.14 | 1.00 |
| 1973-1983; Age 40-49 | 1.36 | 1.00 |
| 1963-1973; Age 50-59 | 1.33 | 1.00 |
| 1953-1963; Age 60-69 | 1.09 | 1.00 |
| 1943-1953; Age 70-79 | 2.09 | 1.18 |
| 1943 or Earlier; Age 80 or Older | 2.67 | 1.00 |
| Mother Absence/Presence (Ref: Present) |  |  |
| Absent | 1.30 | 1.00 |
| Father Absence/Presence (Ref: Present) |  |  |
| Absent | 1.57 | 1.00 |
| Childhood Religion (Ref: No Religion/Atheist/Agnostic) |  |  |
| Christianity | 2.50 | 1.49 |
| Some Other Religion | 4.31 | 2.07 |

**Table S18a: Nationally-Representative Descriptive Statistics of the Observed Sample (Sweden)**

| Variable | Proportion | Frequency |
| --- | --- | --- |
| Relationship with Mother |  |  |
| Very Good | 0.58 | 8743 |
| Somewhat Good | 0.30 | 4513 |
| Somewhat Bad | 0.08 | 1194 |
| Very Bad | 0.02 | 372 |
| Not Applicable | 0.01 | 216 |
| Missing | 0.00 | 30 |
| Relationship with Father |  |  |
| Very Good | 0.47 | 7134 |
| Somewhat Good | 0.32 | 4885 |
| Somewhat Bad | 0.11 | 1588 |
| Very Bad | 0.05 | 725 |
| Not Applicable | 0.05 | 720 |
| Missing | 0.00 | 16 |
| Parent Marital Status |  |  |
| Married | 0.72 | 10887 |
| Divorced | 0.13 | 1927 |
| Never Married | 0.12 | 1747 |
| One or Both Had Died | 0.02 | 362 |
| Missing | 0.01 | 145 |
| Childhood Income |  |  |
| Lived Comfortably | 0.39 | 5951 |
| Got By | 0.51 | 7717 |
| Found it Difficult | 0.08 | 1238 |
| Found it Very Difficult | 0.01 | 140 |
| Missing | 0.00 | 22 |
| Childhood Abuse |  |  |
| Yes | 0.15 | 2288 |
| No | 0.85 | 12735 |
| Missing | 0.00 | 45 |
| Outsider |  |  |
| Yes | 0.12 | 1867 |
| No | 0.86 | 13034 |
| Not Applicable | 0.01 | 139 |
| Missing | 0.00 | 29 |
| Childhood Health |  |  |
| Excellent | 0.38 | 5733 |
| Very Good | 0.34 | 5124 |
| Good | 0.18 | 2669 |
| Fair | 0.07 | 1108 |
| Poor | 0.03 | 397 |
| Missing | 0.00 | 38 |
| Immigration Status |  |  |
| Born in This Country | 0.92 | 13922 |
| Born in Another Country | 0.07 | 1052 |
| Missing | 0.01 | 94 |
| Childhood Service Attendance |  |  |
| At Least 1/Week | 0.06 | 955 |
| 1-3/Month | 0.09 | 1362 |
| <1/Month | 0.41 | 6224 |
| Never | 0.43 | 6472 |
| Missing | 0.00 | 54 |
| Gender |  |  |
| Male | 0.50 | 7536 |
| Female | 0.50 | 7493 |
| Other | 0.00 | 27 |
| Missing | 0.00 | 12 |
| Year of Birth |  |  |
| 1998-2005; Age 18-24 | 0.10 | 1515 |
| 1993-1998; Age 25-29 | 0.09 | 1399 |
| 1983-1993; Age 30-39 | 0.16 | 2398 |
| 1973-1983; Age 40-49 | 0.15 | 2221 |
| 1963-1973; Age 50-59 | 0.17 | 2493 |
| 1953-1963; Age 60-69 | 0.14 | 2168 |
| 1943-1953; Age 70-79 | 0.15 | 2253 |
| 1943 or Earlier; 80 or Older | 0.04 | 621 |
| Missing | . | . |
| Childhood Religion |  |  |
| Christianity | 0.70 | 10617 |
| Islam | 0.03 | 462 |
| Hinduism | 0.00 | 16 |
| Buddhism | 0.00 | 41 |
| Judaism | 0.00 | 51 |
| Sikhism | 0.00 | 9 |
| Baha'i | 0.00 | 3 |
| Jainism | . | . |
| Shinto | 0.00 | 1 |
| Taoism | . | . |
| Confucianism | 0.00 | 4 |
| Primal, Animist, or Folk Religion | 0.00 | 31 |
| Spiritism | . | . |
| African-Derived | . | . |
| Chinese | . | . |
| Some Other Religion | 0.00 | 69 |
| No Religion/Atheist/Agnostic | 0.25 | 3738 |
| Missing | 0.00 | 26 |
| Race/Ethnicity |  |  |
| No Data | . | . |

**Table S18b: Variations Across Childhood Predictors (Sweden)**

| Variable | IRR | SE | Prob | LCI | UCI | Global p-value |
| --- | --- | --- | --- | --- | --- | --- |
| Relationship with Mother (Ref: Very/Somewhat Bad) |  |  |  |  |  |  |
| Very/Somewhat Good | 1.01 | 0.10 | 0.93 | 0.83 | 1.23 | 0.93 |
| Relationship with Father (Ref: Very/Somewhat Bad) |  |  |  |  |  |  |
| Very/Somewhat Good | 1.03 | 0.09 | 0.76 | 0.86 | 1.22 | 0.76 |
| Parent Marital Status (Ref: Married) |  |  |  |  |  |  |
| Divorced | 0.98 | 0.09 | 0.85 | 0.82 | 1.18 | 0.62 |
| Never Married | 0.88 | 0.10 | 0.25 | 0.71 | 1.09 | . |
| One or Both Had Died | 0.82 | 0.22 | 0.45 | 0.48 | 1.38 | . |
| Childhood Income (Ref: Got By) |  |  |  |  |  |  |
| Lived Comfortably | 1.00 | 0.06 | 0.94 | 0.89 | 1.14 | 0.82 |
| Found it Difficult | 1.10 | 0.11 | 0.36 | 0.90 | 1.34 | . |
| Found it Very Difficult | 1.09 | 0.23 | 0.70 | 0.72 | 1.65 | . |
| Childhood Abuse (Ref: No) |  |  |  |  |  |  |
| Yes | 1.18 | 0.09 | 0.03 | 1.02 | 1.36 | 0.03 |
| Outsider (Ref: No) |  |  |  |  |  |  |
| Yes | 1.26 | 0.10 | 0.01 | 1.07 | 1.48 | 0.01 |
| Childhood Health (Ref: Good) |  |  |  |  |  |  |
| Excellent | 1.06 | 0.09 | 0.50 | 0.90 | 1.25 | 0.74 |
| Very Good | 0.97 | 0.08 | 0.72 | 0.83 | 1.14 | . |
| Fair | 1.05 | 0.12 | 0.68 | 0.84 | 1.32 | . |
| Poor | 0.97 | 0.18 | 0.88 | 0.68 | 1.38 | . |
| Immigration Status (Ref: Born in This Country) |  |  |  |  |  |  |
| Born in Another Country | 1.30 | 0.11 | 0.00 | 1.10 | 1.54 | 0.00 |
| Childhood Service Attendance (Ref: Never) |  |  |  |  |  |  |
| At Least 1/Week | 7.11 | 0.63 | 0.00 | 5.97 | 8.47 | 0.00 |
| 1-3/Month | 4.24 | 0.40 | 0.00 | 3.51 | 5.11 | . |
| <1/Month | 1.67 | 0.15 | 0.00 | 1.41 | 1.98 | . |
| Gender (Ref: Male) |  |  |  |  |  |  |
| Female | 1.17 | 0.07 | 0.01 | 1.05 | 1.31 | 0.02 |
| Other | 1.05 | 0.67 | 0.94 | 0.30 | 3.69 | . |
| Year of Birth (Ref: 1998-2005) |  |  |  |  |  |  |
| 1993-1998; Age 25-29 | 0.98 | 0.10 | 0.83 | 0.80 | 1.20 | 0.07 |
| 1983-1993; Age 30-39 | 0.79 | 0.08 | 0.02 | 0.65 | 0.97 | . |
| 1973-1983; Age 40-49 | 0.71 | 0.08 | 0.00 | 0.57 | 0.89 | . |
| 1963-1973; Age 50-59 | 0.90 | 0.10 | 0.30 | 0.73 | 1.10 | . |
| 1953-1963; Age 60-69 | 0.85 | 0.09 | 0.13 | 0.68 | 1.05 | . |
| 1943-1953; Age 70-79 | 0.90 | 0.10 | 0.35 | 0.73 | 1.12 | . |
| 1943 or Earlier; Age 80 or Older | 0.86 | 0.13 | 0.32 | 0.65 | 1.15 | . |
| Mother Absence/Presence (Ref: Present) |  |  |  |  |  |  |
| Absent | 1.08 | 0.18 | 0.65 | 0.78 | 1.50 | 0.65 |
| Father Absence/Presence (Ref: Present) |  |  |  |  |  |  |
| Absent | 0.83 | 0.13 | 0.26 | 0.61 | 1.14 | 0.26 |
| Childhood Religion (Ref: No Religion/Atheist/Agnostic) |  |  |  |  |  |  |
| Christianity | 1.86 | 0.20 | 0.00 | 1.51 | 2.30 | 0.00 |
| Some Other Religion | 3.51 | 0.49 | 0.00 | 2.68 | 4.61 | . |

**Table S18c: E-Values and E-Value Limits (Sweden)**

| Variable | E-Value | E-Value Limit |
| --- | --- | --- |
| Relationship with Mother (Ref: Very/Somewhat Bad) |  |  |
| Very/Somewhat Good | 1.13 | 1.00 |
| Relationship with Father (Ref: Very/Somewhat Bad) |  |  |
| Very/Somewhat Good | 1.25 | 1.00 |
| Parent Marital Status (Ref: Married) |  |  |
| Divorced | 1.19 | 1.00 |
| Never Married | 1.71 | 1.00 |
| One or Both Had Died | 2.03 | 1.00 |
| Childhood Income (Ref: Got By) |  |  |
| Lived Comfortably | 1.09 | 1.00 |
| Found it Difficult | 1.55 | 1.00 |
| Found it Very Difficult | 1.51 | 1.00 |
| Childhood Abuse (Ref: No) |  |  |
| Yes | 1.86 | 1.10 |
| Outsider (Ref: No) |  |  |
| Yes | 2.15 | 1.22 |
| Childhood Health (Ref: Good) |  |  |
| Excellent | 1.39 | 1.00 |
| Very Good | 1.26 | 1.00 |
| Fair | 1.36 | 1.00 |
| Poor | 1.25 | 1.00 |
| Immigration Status (Ref: Born in This Country) |  |  |
| Born in Another Country | 2.32 | 1.28 |
| Childhood Service Attendance (Ref: Never) |  |  |
| At Least 1/Week | 35.56 | 4.32 |
| 1-3/Month | 16.30 | 3.15 |
| <1/Month | 3.68 | 1.66 |
| Gender (Ref: Male) |  |  |
| Female | 1.84 | 1.18 |
| Other | 1.36 | 1.00 |
| Year of Birth (Ref: 1998-2005) |  |  |
| 1993-1998; Age 25-29 | 1.22 | 1.00 |
| 1983-1993; Age 30-39 | 2.17 | 1.14 |
| 1973-1983; Age 40-49 | 2.68 | 1.32 |
| 1963-1973; Age 50-59 | 1.63 | 1.00 |
| 1953-1963; Age 60-69 | 1.87 | 1.00 |
| 1943-1953; Age 70-79 | 1.61 | 1.00 |
| 1943 or Earlier; Age 80 or Older | 1.79 | 1.00 |
| Mother Absence/Presence (Ref: Present) |  |  |
| Absent | 1.48 | 1.00 |
| Father Absence/Presence (Ref: Present) |  |  |
| Absent | 1.94 | 1.00 |
| Childhood Religion (Ref: No Religion/Atheist/Agnostic) |  |  |
| Christianity | 4.44 | 1.76 |
| Some Other Religion | 12.23 | 2.66 |

**Table S19a: Nationally-Representative Descriptive Statistics of the Observed Sample (Tanzania)**

| Variable | Proportion | Frequency |
| --- | --- | --- |
| Relationship with Mother |  |  |
| Very Good | 0.85 | 7739 |
| Somewhat Good | 0.09 | 796 |
| Somewhat Bad | 0.01 | 84 |
| Very Bad | 0.01 | 84 |
| Not Applicable | 0.03 | 303 |
| Missing | 0.01 | 70 |
| Relationship with Father |  |  |
| Very Good | 0.75 | 6831 |
| Somewhat Good | 0.12 | 1101 |
| Somewhat Bad | 0.02 | 203 |
| Very Bad | 0.03 | 247 |
| Not Applicable | 0.06 | 550 |
| Missing | 0.02 | 142 |
| Parent Marital Status |  |  |
| Married | 0.76 | 6929 |
| Divorced | 0.07 | 678 |
| Never Married | 0.08 | 751 |
| One or Both Had Died | 0.03 | 313 |
| Missing | 0.04 | 404 |
| Childhood Income |  |  |
| Lived Comfortably | 0.29 | 2611 |
| Got By | 0.32 | 2909 |
| Found it Difficult | 0.30 | 2679 |
| Found it Very Difficult | 0.09 | 814 |
| Missing | 0.01 | 61 |
| Childhood Abuse |  |  |
| Yes | 0.08 | 716 |
| No | 0.92 | 8328 |
| Missing | 0.00 | 32 |
| Outsider |  |  |
| Yes | 0.08 | 734 |
| No | 0.92 | 8320 |
| Not Applicable | 0.00 | 4 |
| Missing | 0.00 | 17 |
| Childhood Health |  |  |
| Excellent | 0.27 | 2406 |
| Very Good | 0.22 | 2036 |
| Good | 0.32 | 2946 |
| Fair | 0.13 | 1177 |
| Poor | 0.05 | 456 |
| Missing | 0.01 | 54 |
| Immigration Status |  |  |
| Born in This Country | 1.00 | 9048 |
| Born in Another Country | 0.00 | 25 |
| Missing | 0.00 | 1 |
| Childhood Service Attendance |  |  |
| At Least 1/Week | 0.61 | 5580 |
| 1-3/Month | 0.26 | 2383 |
| <1/Month | 0.04 | 333 |
| Never | 0.07 | 595 |
| Missing | 0.02 | 184 |
| Gender |  |  |
| Male | 0.47 | 4299 |
| Female | 0.53 | 4776 |
| Other | . | . |
| Missing | . | . |
| Year of Birth |  |  |
| 1998-2005; Age 18-24 | 0.25 | 2284 |
| 1993-1998; Age 25-29 | 0.15 | 1349 |
| 1983-1993; Age 30-39 | 0.23 | 2060 |
| 1973-1983; Age 40-49 | 0.17 | 1503 |
| 1963-1973; Age 50-59 | 0.10 | 912 |
| 1953-1963; Age 60-69 | 0.06 | 575 |
| 1943-1953; Age 70-79 | 0.03 | 297 |
| 1943 or Earlier; 80 or Older | 0.01 | 93 |
| Missing | 0.00 | 2 |
| Childhood Religion |  |  |
| Christianity | 0.62 | 5651 |
| Islam | 0.34 | 3060 |
| Hinduism | . | . |
| Buddhism | . | . |
| Judaism | . | . |
| Sikhism | . | . |
| Baha'i | 0.00 | 1 |
| Jainism | . | . |
| Shinto | . | . |
| Taoism | . | . |
| Confucianism | . | . |
| Primal, Animist, or Folk Religion | 0.00 | 11 |
| Spiritism | . | . |
| African-Derived | . | . |
| Chinese | . | . |
| Some Other Religion | . | . |
| No Religion/Atheist/Agnostic | 0.04 | 345 |
| Missing | 0.00 | 7 |
| Race/Ethnicity |  |  |
| African | 1.00 | 9060 |
| Indian | 0.00 | 3 |
| Arab | 0.00 | 11 |
| Other | . | . |
| Missing | 0.00 | 2 |

**Table S19b: Variations Across Childhood Predictors (Tanzania)**

| Variable | IRR | SE | Prob | LCI | UCI | Global p-value |
| --- | --- | --- | --- | --- | --- | --- |
| Relationship with Mother (Ref: Very/Somewhat Bad) |  |  |  |  |  |  |
| Very/Somewhat Good | 0.94 | 0.04 | 0.12 | 0.86 | 1.02 | 0.12 |
| Relationship with Father (Ref: Very/Somewhat Bad) |  |  |  |  |  |  |
| Very/Somewhat Good | 0.99 | 0.03 | 0.88 | 0.93 | 1.06 | 0.88 |
| Parent Marital Status (Ref: Married) |  |  |  |  |  |  |
| Divorced | 0.94 | 0.03 | 0.08 | 0.87 | 1.01 | 0.00 |
| Never Married | 0.87 | 0.04 | 0.00 | 0.80 | 0.94 | . |
| One or Both Had Died | 0.94 | 0.04 | 0.17 | 0.86 | 1.03 | . |
| Childhood Income (Ref: Got By) |  |  |  |  |  |  |
| Lived Comfortably | 0.94 | 0.02 | 0.00 | 0.91 | 0.98 | 0.00 |
| Found it Difficult | 0.94 | 0.02 | 0.00 | 0.91 | 0.97 | . |
| Found it Very Difficult | 0.96 | 0.03 | 0.23 | 0.90 | 1.02 | . |
| Childhood Abuse (Ref: No) |  |  |  |  |  |  |
| Yes | 0.93 | 0.03 | 0.03 | 0.87 | 0.99 | 0.03 |
| Outsider (Ref: No) |  |  |  |  |  |  |
| Yes | 1.00 | 0.03 | 0.95 | 0.94 | 1.06 | 0.95 |
| Childhood Health (Ref: Good) |  |  |  |  |  |  |
| Excellent | 1.05 | 0.02 | 0.02 | 1.01 | 1.09 | 0.08 |
| Very Good | 1.00 | 0.02 | 0.99 | 0.96 | 1.05 | . |
| Fair | 1.03 | 0.03 | 0.22 | 0.98 | 1.09 | . |
| Poor | 1.04 | 0.04 | 0.32 | 0.96 | 1.12 | . |
| Immigration Status (Ref: Born in This Country) |  |  |  |  |  |  |
| Born in Another Country | 1.18 | 0.08 | 0.01 | 1.04 | 1.34 | 0.01 |
| Childhood Service Attendance (Ref: Never) |  |  |  |  |  |  |
| At Least 1/Week | 1.15 | 0.05 | 0.00 | 1.05 | 1.26 | 0.01 |
| 1-3/Month | 1.11 | 0.05 | 0.03 | 1.01 | 1.23 | . |
| <1/Month | 1.07 | 0.06 | 0.26 | 0.95 | 1.21 | . |
| Gender (Ref: Male) |  |  |  |  |  |  |
| Female | 1.06 | 0.02 | 0.00 | 1.03 | 1.09 | 0.00 |
| Other | 1.00 | . | . | . | . | . |
| Year of Birth (Ref: 1998-2005) |  |  |  |  |  |  |
| 1993-1998; Age 25-29 | 1.04 | 0.03 | 0.15 | 0.99 | 1.10 | 0.00 |
| 1983-1993; Age 30-39 | 1.06 | 0.02 | 0.00 | 1.02 | 1.11 | . |
| 1973-1983; Age 40-49 | 1.07 | 0.03 | 0.01 | 1.02 | 1.12 | . |
| 1963-1973; Age 50-59 | 1.12 | 0.03 | 0.00 | 1.07 | 1.17 | . |
| 1953-1963; Age 60-69 | 1.12 | 0.04 | 0.00 | 1.05 | 1.19 | . |
| 1943-1953; Age 70-79 | 1.13 | 0.06 | 0.02 | 1.02 | 1.24 | . |
| 1943 or Earlier; Age 80 or Older | 1.16 | 0.08 | 0.04 | 1.01 | 1.34 | . |
| Mother Absence/Presence (Ref: Present) |  |  |  |  |  |  |
| Absent | 0.92 | 0.04 | 0.05 | 0.84 | 1.00 | 0.05 |
| Father Absence/Presence (Ref: Present) |  |  |  |  |  |  |
| Absent | 1.04 | 0.03 | 0.24 | 0.98 | 1.11 | 0.24 |
| Childhood Religion (Ref: No Religion/Atheist/Agnostic) |  |  |  |  |  |  |
| Christianity | 1.02 | 0.07 | 0.80 | 0.89 | 1.16 | 0.35 |
| Islam | 1.06 | 0.07 | 0.41 | 0.92 | 1.21 | . |
| Some Other Religion | 0.98 | 0.20 | 0.91 | 0.65 | 1.47 | . |
| Race/Ethnicity (Ref: Ethnic Plurality) |  |  |  |  |  |  |
| Ethnic Minority | 1.30 | 0.03 | 0.00 | 1.25 | 1.36 | 0.00 |

**Table S19c: E-Values and E-Value Limits (Tanzania)**

| Variable | E-Value | E-Value Limit |
| --- | --- | --- |
| Relationship with Mother (Ref: Very/Somewhat Bad) |  |  |
| Very/Somewhat Good | 1.24 | 1.00 |
| Relationship with Father (Ref: Very/Somewhat Bad) |  |  |
| Very/Somewhat Good | 1.06 | 1.00 |
| Parent Marital Status (Ref: Married) |  |  |
| Divorced | 1.23 | 1.00 |
| Never Married | 1.39 | 1.21 |
| One or Both Had Died | 1.23 | 1.00 |
| Childhood Income (Ref: Got By) |  |  |
| Lived Comfortably | 1.22 | 1.10 |
| Found it Difficult | 1.24 | 1.15 |
| Found it Very Difficult | 1.17 | 1.00 |
| Childhood Abuse (Ref: No) |  |  |
| Yes | 1.25 | 1.06 |
| Outsider (Ref: No) |  |  |
| Yes | 1.03 | 1.00 |
| Childhood Health (Ref: Good) |  |  |
| Excellent | 1.19 | 1.06 |
| Very Good | 1.02 | 1.00 |
| Fair | 1.16 | 1.00 |
| Poor | 1.17 | 1.00 |
| Immigration Status (Ref: Born in This Country) |  |  |
| Born in Another Country | 1.43 | 1.17 |
| Childhood Service Attendance (Ref: Never) |  |  |
| At Least 1/Week | 1.38 | 1.18 |
| 1-3/Month | 1.32 | 1.07 |
| <1/Month | 1.24 | 1.00 |
| Gender (Ref: Male) |  |  |
| Female | 1.22 | 1.14 |
| Other | 1.00 | 1.00 |
| Year of Birth (Ref: 1998-2005) |  |  |
| 1993-1998; Age 25-29 | 1.17 | 1.00 |
| 1983-1993; Age 30-39 | 1.23 | 1.11 |
| 1973-1983; Age 40-49 | 1.24 | 1.11 |
| 1963-1973; Age 50-59 | 1.33 | 1.21 |
| 1953-1963; Age 60-69 | 1.33 | 1.17 |
| 1943-1953; Age 70-79 | 1.35 | 1.12 |
| 1943 or Earlier; Age 80 or Older | 1.40 | 1.06 |
| Mother Absence/Presence (Ref: Present) |  |  |
| Absent | 1.28 | 1.00 |
| Father Absence/Presence (Ref: Present) |  |  |
| Absent | 1.17 | 1.00 |
| Childhood Religion (Ref: No Religion/Atheist/Agnostic) |  |  |
| Christianity | 1.11 | 1.00 |
| Islam | 1.22 | 1.00 |
| Some Other Religion | 1.13 | 1.00 |
| Race/Ethnicity (Ref: Ethnic Plurality) |  |  |
| Ethnic Minority | 1.60 | 1.48 |

**Table S20a: Nationally-Representative Descriptive Statistics of the Observed Sample (Turkey)**

| Variable | Proportion | Frequency |
| --- | --- | --- |
| Relationship with Mother |  |  |
| Very Good | 0.66 | 970 |
| Somewhat Good | 0.27 | 401 |
| Somewhat Bad | 0.03 | 48 |
| Very Bad | 0.02 | 26 |
| Not Applicable | 0.01 | 21 |
| Missing | 0.00 | 7 |
| Relationship with Father |  |  |
| Very Good | 0.54 | 795 |
| Somewhat Good | 0.29 | 425 |
| Somewhat Bad | 0.05 | 73 |
| Very Bad | 0.06 | 95 |
| Not Applicable | 0.04 | 60 |
| Missing | 0.02 | 25 |
| Parent Marital Status |  |  |
| Married | 0.90 | 1325 |
| Divorced | 0.04 | 57 |
| Never Married | 0.00 | 7 |
| One or Both Had Died | 0.04 | 61 |
| Missing | 0.02 | 23 |
| Childhood Income |  |  |
| Lived Comfortably | 0.34 | 498 |
| Got By | 0.44 | 647 |
| Found it Difficult | 0.15 | 218 |
| Found it Very Difficult | 0.07 | 108 |
| Missing | 0.00 | 2 |
| Childhood Abuse |  |  |
| Yes | 0.11 | 158 |
| No | 0.88 | 1290 |
| Missing | 0.02 | 25 |
| Outsider |  |  |
| Yes | 0.11 | 157 |
| No | 0.89 | 1306 |
| Not Applicable | 0.00 | 5 |
| Missing | 0.00 | 5 |
| Childhood Health |  |  |
| Excellent | 0.26 | 377 |
| Very Good | 0.28 | 410 |
| Good | 0.28 | 419 |
| Fair | 0.15 | 220 |
| Poor | 0.03 | 47 |
| Missing | 0.00 | 0 |
| Immigration Status |  |  |
| Born in This Country | 0.96 | 1415 |
| Born in Another Country | 0.04 | 58 |
| Missing | . | . |
| Childhood Service Attendance |  |  |
| At Least 1/Week | 0.41 | 609 |
| 1-3/Month | 0.16 | 238 |
| <1/Month | 0.15 | 225 |
| Never | 0.26 | 383 |
| Missing | 0.01 | 18 |
| Gender |  |  |
| Male | 0.51 | 754 |
| Female | 0.49 | 719 |
| Other | . | . |
| Missing | . | . |
| Year of Birth |  |  |
| 1998-2005; Age 18-24 | 0.15 | 222 |
| 1993-1998; Age 25-29 | 0.10 | 152 |
| 1983-1993; Age 30-39 | 0.21 | 315 |
| 1973-1983; Age 40-49 | 0.21 | 312 |
| 1963-1973; Age 50-59 | 0.15 | 225 |
| 1953-1963; Age 60-69 | 0.11 | 164 |
| 1943-1953; Age 70-79 | 0.04 | 65 |
| 1943 or Earlier; 80 or Older | 0.01 | 18 |
| Missing | . | . |
| Childhood Religion |  |  |
| Christianity | 0.00 | 1 |
| Islam | 0.98 | 1439 |
| Hinduism | . | . |
| Buddhism | . | . |
| Judaism | 0.00 | 1 |
| Sikhism | . | . |
| Baha'i | . | . |
| Jainism | . | . |
| Shinto | . | . |
| Taoism | . | . |
| Confucianism | . | . |
| Primal, Animist, or Folk Religion | . | . |
| Spiritism | . | . |
| African-Derived | . | . |
| Chinese | . | . |
| Some Other Religion | . | . |
| No Religion/Atheist/Agnostic | 0.01 | 13 |
| Missing | 0.01 | 19 |
| Race/Ethnicity |  |  |
| Turkish | 0.70 | 1030 |
| Kurdish/Zaza | 0.17 | 252 |
| Arab | 0.03 | 51 |
| Laz | 0.02 | 25 |
| Circassian | 0.01 | 19 |
| Bosnian | 0.00 | 5 |
| Armenian | 0.00 | 1 |
| Georgian | 0.00 | 4 |
| Uyghur | 0.00 | 1 |
| Jewish | . | . |
| Albanian | 0.01 | 8 |
| Greek | 0.00 | 1 |
| Azeri | 0.01 | 9 |
| Other | 0.04 | 58 |
| Missing | 0.01 | 9 |

**Table S20b: Variations Across Childhood Predictors (Turkey)**

| Variable | IRR | SE | Prob | LCI | UCI | Global p-value |
| --- | --- | --- | --- | --- | --- | --- |
| Relationship with Mother (Ref: Very/Somewhat Bad) |  |  |  |  |  |  |
| Very/Somewhat Good | 0.95 | 0.10 | 0.61 | 0.78 | 1.16 | 0.61 |
| Relationship with Father (Ref: Very/Somewhat Bad) |  |  |  |  |  |  |
| Very/Somewhat Good | 1.03 | 0.07 | 0.64 | 0.90 | 1.19 | 0.64 |
| Parent Marital Status (Ref: Married) |  |  |  |  |  |  |
| Divorced | 1.07 | 0.09 | 0.46 | 0.90 | 1.27 | 0.45 |
| Never Married | 0.81 | 0.17 | 0.33 | 0.54 | 1.24 | . |
| One or Both Had Died | 1.09 | 0.08 | 0.25 | 0.94 | 1.25 | . |
| Childhood Income (Ref: Got By) |  |  |  |  |  |  |
| Lived Comfortably | 1.04 | 0.04 | 0.37 | 0.96 | 1.13 | 0.84 |
| Found it Difficult | 1.01 | 0.06 | 0.89 | 0.89 | 1.14 | . |
| Found it Very Difficult | 1.03 | 0.08 | 0.76 | 0.87 | 1.20 | . |
| Childhood Abuse (Ref: No) |  |  |  |  |  |  |
| Yes | 0.88 | 0.06 | 0.06 | 0.76 | 1.01 | 0.06 |
| Outsider (Ref: No) |  |  |  |  |  |  |
| Yes | 0.97 | 0.07 | 0.71 | 0.85 | 1.12 | 0.71 |
| Childhood Health (Ref: Good) |  |  |  |  |  |  |
| Excellent | 0.98 | 0.05 | 0.77 | 0.89 | 1.09 | 0.94 |
| Very Good | 1.00 | 0.05 | 0.95 | 0.91 | 1.11 | . |
| Fair | 1.00 | 0.06 | 1.00 | 0.88 | 1.13 | . |
| Poor | 0.86 | 0.16 | 0.43 | 0.59 | 1.25 | . |
| Immigration Status (Ref: Born in This Country) |  |  |  |  |  |  |
| Born in Another Country | 1.05 | 0.11 | 0.64 | 0.86 | 1.29 | 0.64 |
| Childhood Service Attendance (Ref: Never) |  |  |  |  |  |  |
| At Least 1/Week | 1.46 | 0.08 | 0.00 | 1.30 | 1.63 | 0.00 |
| 1-3/Month | 1.21 | 0.08 | 0.01 | 1.05 | 1.38 | . |
| <1/Month | 1.02 | 0.08 | 0.81 | 0.88 | 1.18 | . |
| Gender (Ref: Male) |  |  |  |  |  |  |
| Female | 1.33 | 0.05 | 0.00 | 1.23 | 1.44 | 0.00 |
| Other | 1.00 | . | . | . | . | . |
| Year of Birth (Ref: 1998-2005) |  |  |  |  |  |  |
| 1993-1998; Age 25-29 | 1.14 | 0.09 | 0.12 | 0.97 | 1.33 | 0.01 |
| 1983-1993; Age 30-39 | 1.29 | 0.09 | 0.00 | 1.12 | 1.47 | . |
| 1973-1983; Age 40-49 | 1.26 | 0.09 | 0.00 | 1.10 | 1.45 | . |
| 1963-1973; Age 50-59 | 1.27 | 0.10 | 0.00 | 1.09 | 1.47 | . |
| 1953-1963; Age 60-69 | 1.35 | 0.12 | 0.00 | 1.13 | 1.60 | . |
| 1943-1953; Age 70-79 | 1.27 | 0.18 | 0.09 | 0.96 | 1.68 | . |
| 1943 or Earlier; Age 80 or Older | 1.47 | 0.25 | 0.02 | 1.05 | 2.05 | . |
| Mother Absence/Presence (Ref: Present) |  |  |  |  |  |  |
| Absent | 0.84 | 0.11 | 0.19 | 0.66 | 1.09 | 0.19 |
| Father Absence/Presence (Ref: Present) |  |  |  |  |  |  |
| Absent | 1.11 | 0.10 | 0.25 | 0.93 | 1.33 | 0.25 |
| Childhood Religion (Ref: Islam) |  |  |  |  |  |  |
| Some Other Religion | 0.38 | 0.14 | 0.01 | 0.18 | 0.79 | 0.01 |
| Race/Ethnicity (Ref: Ethnic Plurality) |  |  |  |  |  |  |
| Ethnic Minority | 1.03 | 0.05 | 0.53 | 0.94 | 1.12 | 0.53 |

**Table S20c: E-Values and E-Value Limits (Turkey)**

| Variable | E-Value | E-Value Limit |
| --- | --- | --- |
| Relationship with Mother (Ref: Very/Somewhat Bad) |  |  |
| Very/Somewhat Good | 1.21 | 1.00 |
| Relationship with Father (Ref: Very/Somewhat Bad) |  |  |
| Very/Somewhat Good | 1.16 | 1.00 |
| Parent Marital Status (Ref: Married) |  |  |
| Divorced | 1.24 | 1.00 |
| Never Married | 1.52 | 1.00 |
| One or Both Had Died | 1.28 | 1.00 |
| Childhood Income (Ref: Got By) |  |  |
| Lived Comfortably | 1.18 | 1.00 |
| Found it Difficult | 1.08 | 1.00 |
| Found it Very Difficult | 1.14 | 1.00 |
| Childhood Abuse (Ref: No) |  |  |
| Yes | 1.38 | 1.00 |
| Outsider (Ref: No) |  |  |
| Yes | 1.14 | 1.00 |
| Childhood Health (Ref: Good) |  |  |
| Excellent | 1.11 | 1.00 |
| Very Good | 1.05 | 1.00 |
| Fair | 1.00 | 1.00 |
| Poor | 1.42 | 1.00 |
| Immigration Status (Ref: Born in This Country) |  |  |
| Born in Another Country | 1.21 | 1.00 |
| Childhood Service Attendance (Ref: Never) |  |  |
| At Least 1/Week | 1.82 | 1.54 |
| 1-3/Month | 1.49 | 1.19 |
| <1/Month | 1.12 | 1.00 |
| Gender (Ref: Male) |  |  |
| Female | 1.66 | 1.46 |
| Other | 1.00 | 1.00 |
| Year of Birth (Ref: 1998-2005) |  |  |
| 1993-1998; Age 25-29 | 1.37 | 1.00 |
| 1983-1993; Age 30-39 | 1.60 | 1.31 |
| 1973-1983; Age 40-49 | 1.57 | 1.28 |
| 1963-1973; Age 50-59 | 1.58 | 1.27 |
| 1953-1963; Age 60-69 | 1.68 | 1.33 |
| 1943-1953; Age 70-79 | 1.58 | 1.00 |
| 1943 or Earlier; Age 80 or Older | 1.83 | 1.19 |
| Mother Absence/Presence (Ref: Present) |  |  |
| Absent | 1.45 | 1.00 |
| Father Absence/Presence (Ref: Present) |  |  |
| Absent | 1.33 | 1.00 |
| Childhood Religion (Ref: Islam) |  |  |
| Some Other Religion | 3.00 | 1.50 |
| Race/Ethnicity (Ref: Ethnic Plurality) |  |  |
| Ethnic Minority | 1.15 | 1.00 |

**Table S21a: Nationally-Representative Descriptive Statistics of the Observed Sample (United Kingdom)**

| Variable | Proportion | Frequency |
| --- | --- | --- |
| Relationship with Mother |  |  |
| Very Good | 0.64 | 3435 |
| Somewhat Good | 0.25 | 1338 |
| Somewhat Bad | 0.06 | 325 |
| Very Bad | 0.03 | 150 |
| Not Applicable | 0.02 | 92 |
| Missing | 0.01 | 27 |
| Relationship with Father |  |  |
| Very Good | 0.54 | 2907 |
| Somewhat Good | 0.26 | 1383 |
| Somewhat Bad | 0.08 | 407 |
| Very Bad | 0.06 | 321 |
| Not Applicable | 0.06 | 321 |
| Missing | 0.01 | 29 |
| Parent Marital Status |  |  |
| Married | 0.81 | 4343 |
| Divorced | 0.09 | 481 |
| Never Married | 0.06 | 315 |
| One or Both Had Died | 0.03 | 154 |
| Missing | 0.01 | 75 |
| Childhood Income |  |  |
| Lived Comfortably | 0.48 | 2552 |
| Got By | 0.36 | 1933 |
| Found it Difficult | 0.12 | 632 |
| Found it Very Difficult | 0.04 | 230 |
| Missing | 0.00 | 22 |
| Childhood Abuse |  |  |
| Yes | 0.16 | 864 |
| No | 0.83 | 4455 |
| Missing | 0.01 | 49 |
| Outsider |  |  |
| Yes | 0.19 | 1017 |
| No | 0.80 | 4308 |
| Not Applicable | 0.01 | 32 |
| Missing | 0.00 | 12 |
| Childhood Health |  |  |
| Excellent | 0.40 | 2154 |
| Very Good | 0.32 | 1736 |
| Good | 0.19 | 995 |
| Fair | 0.06 | 332 |
| Poor | 0.02 | 130 |
| Missing | 0.00 | 20 |
| Immigration Status |  |  |
| Born in This Country | 0.87 | 4659 |
| Born in Another Country | 0.13 | 682 |
| Missing | 0.00 | 27 |
| Childhood Service Attendance |  |  |
| At Least 1/Week | 0.32 | 1732 |
| 1-3/Month | 0.14 | 733 |
| <1/Month | 0.17 | 903 |
| Never | 0.37 | 1972 |
| Missing | 0.01 | 28 |
| Gender |  |  |
| Male | 0.48 | 2557 |
| Female | 0.52 | 2789 |
| Other | 0.00 | 14 |
| Missing | 0.00 | 9 |
| Year of Birth |  |  |
| 1998-2005; Age 18-24 | 0.09 | 490 |
| 1993-1998; Age 25-29 | 0.07 | 391 |
| 1983-1993; Age 30-39 | 0.18 | 946 |
| 1973-1983; Age 40-49 | 0.15 | 827 |
| 1963-1973; Age 50-59 | 0.18 | 949 |
| 1953-1963; Age 60-69 | 0.17 | 889 |
| 1943-1953; Age 70-79 | 0.13 | 711 |
| 1943 or Earlier; 80 or Older | 0.03 | 163 |
| Missing | 0.00 | 1 |
| Childhood Religion |  |  |
| Christianity | 0.64 | 3461 |
| Islam | 0.04 | 230 |
| Hinduism | 0.02 | 88 |
| Buddhism | 0.00 | 15 |
| Judaism | 0.01 | 59 |
| Sikhism | 0.01 | 30 |
| Baha'i | 0.00 | 5 |
| Jainism | 0.00 | 0 |
| Shinto | . | . |
| Taoism | 0.00 | 2 |
| Confucianism | 0.00 | 3 |
| Primal, Animist, or Folk Religion | 0.00 | 22 |
| Spiritism | . | . |
| African-Derived | . | . |
| Chinese | . | . |
| Some Other Religion | 0.00 | 24 |
| No Religion/Atheist/Agnostic | 0.26 | 1409 |
| Missing | 0.00 | 21 |
| Race/Ethnicity |  |  |
| Asian | 0.08 | 426 |
| Black | 0.03 | 152 |
| White | 0.87 | 4647 |
| Other | 0.02 | 96 |
| Missing | 0.01 | 47 |

**Table S21b: Variations Across Childhood Predictors (United Kingdom)**

| Variable | IRR | SE | Prob | LCI | UCI | Global p-value |
| --- | --- | --- | --- | --- | --- | --- |
| Relationship with Mother (Ref: Very/Somewhat Bad) |  |  |  |  |  |  |
| Very/Somewhat Good | 1.05 | 0.12 | 0.64 | 0.84 | 1.32 | 0.64 |
| Relationship with Father (Ref: Very/Somewhat Bad) |  |  |  |  |  |  |
| Very/Somewhat Good | 1.10 | 0.11 | 0.38 | 0.89 | 1.34 | 0.38 |
| Parent Marital Status (Ref: Married) |  |  |  |  |  |  |
| Divorced | 0.81 | 0.12 | 0.17 | 0.61 | 1.09 | 0.43 |
| Never Married | 0.82 | 0.15 | 0.28 | 0.57 | 1.18 | . |
| One or Both Had Died | 0.92 | 0.23 | 0.75 | 0.57 | 1.50 | . |
| Childhood Income (Ref: Got By) |  |  |  |  |  |  |
| Lived Comfortably | 0.96 | 0.07 | 0.58 | 0.84 | 1.10 | 0.81 |
| Found it Difficult | 0.91 | 0.10 | 0.38 | 0.73 | 1.13 | . |
| Found it Very Difficult | 0.91 | 0.16 | 0.59 | 0.64 | 1.29 | . |
| Childhood Abuse (Ref: No) |  |  |  |  |  |  |
| Yes | 1.15 | 0.09 | 0.07 | 0.99 | 1.35 | 0.07 |
| Outsider (Ref: No) |  |  |  |  |  |  |
| Yes | 1.36 | 0.10 | 0.00 | 1.18 | 1.57 | 0.00 |
| Childhood Health (Ref: Good) |  |  |  |  |  |  |
| Excellent | 1.19 | 0.12 | 0.08 | 0.98 | 1.44 | 0.03 |
| Very Good | 1.03 | 0.10 | 0.80 | 0.85 | 1.25 | . |
| Fair | 1.40 | 0.21 | 0.02 | 1.05 | 1.87 | . |
| Poor | 1.34 | 0.29 | 0.19 | 0.87 | 2.05 | . |
| Immigration Status (Ref: Born in This Country) |  |  |  |  |  |  |
| Born in Another Country | 1.19 | 0.09 | 0.02 | 1.03 | 1.38 | 0.02 |
| Childhood Service Attendance (Ref: Never) |  |  |  |  |  |  |
| At Least 1/Week | 3.51 | 0.44 | 0.00 | 2.74 | 4.49 | 0.00 |
| 1-3/Month | 2.85 | 0.40 | 0.00 | 2.16 | 3.76 | . |
| <1/Month | 1.43 | 0.22 | 0.02 | 1.06 | 1.94 | . |
| Gender (Ref: Male) |  |  |  |  |  |  |
| Female | 1.00 | 0.06 | 0.98 | 0.88 | 1.13 | 0.00 |
| Other | 0.00 | 0.00 | 0.00 | 0.00 | 0.00 | . |
| Year of Birth (Ref: 1998-2005) |  |  |  |  |  |  |
| 1993-1998; Age 25-29 | 1.22 | 0.17 | 0.16 | 0.93 | 1.61 | 0.00 |
| 1983-1993; Age 30-39 | 1.21 | 0.16 | 0.14 | 0.94 | 1.56 | . |
| 1973-1983; Age 40-49 | 1.03 | 0.15 | 0.83 | 0.78 | 1.36 | . |
| 1963-1973; Age 50-59 | 0.89 | 0.13 | 0.45 | 0.67 | 1.20 | . |
| 1953-1963; Age 60-69 | 0.86 | 0.13 | 0.33 | 0.63 | 1.17 | . |
| 1943-1953; Age 70-79 | 0.93 | 0.15 | 0.64 | 0.68 | 1.26 | . |
| 1943 or Earlier; Age 80 or Older | 1.41 | 0.25 | 0.05 | 1.00 | 2.00 | . |
| Mother Absence/Presence (Ref: Present) |  |  |  |  |  |  |
| Absent | 1.34 | 0.21 | 0.06 | 0.98 | 1.82 | 0.06 |
| Father Absence/Presence (Ref: Present) |  |  |  |  |  |  |
| Absent | 0.80 | 0.14 | 0.19 | 0.57 | 1.12 | 0.19 |
| Childhood Religion (Ref: No Religion/Atheist/Agnostic) |  |  |  |  |  |  |
| Christianity | 1.75 | 0.24 | 0.00 | 1.34 | 2.30 | 0.00 |
| Islam | 2.96 | 0.45 | 0.00 | 2.19 | 3.99 | . |
| Some Other Religion | 1.61 | 0.33 | 0.02 | 1.07 | 2.41 | . |
| Race/Ethnicity (Ref: Ethnic Plurality) |  |  |  |  |  |  |
| Ethnic Minority | 1.42 | 0.13 | 0.00 | 1.18 | 1.70 | 0.00 |

**Table S21c: E-Values and E-Value Limits (United Kingdom)**

| Variable | E-Value | E-Value Limit |
| --- | --- | --- |
| Relationship with Mother (Ref: Very/Somewhat Bad) |  |  |
| Very/Somewhat Good | 1.30 | 1.00 |
| Relationship with Father (Ref: Very/Somewhat Bad) |  |  |
| Very/Somewhat Good | 1.43 | 1.00 |
| Parent Marital Status (Ref: Married) |  |  |
| Divorced | 1.77 | 1.00 |
| Never Married | 1.76 | 1.00 |
| One or Both Had Died | 1.39 | 1.00 |
| Childhood Income (Ref: Got By) |  |  |
| Lived Comfortably | 1.24 | 1.00 |
| Found it Difficult | 1.44 | 1.00 |
| Found it Very Difficult | 1.44 | 1.00 |
| Childhood Abuse (Ref: No) |  |  |
| Yes | 1.59 | 1.00 |
| Outsider (Ref: No) |  |  |
| Yes | 2.08 | 1.39 |
| Childhood Health (Ref: Good) |  |  |
| Excellent | 1.68 | 1.00 |
| Very Good | 1.19 | 1.00 |
| Fair | 2.18 | 1.18 |
| Poor | 2.03 | 1.00 |
| Immigration Status (Ref: Born in This Country) |  |  |
| Born in Another Country | 1.68 | 1.13 |
| Childhood Service Attendance (Ref: Never) |  |  |
| At Least 1/Week | 6.69 | 2.70 |
| 1-3/Month | 5.29 | 2.30 |
| <1/Month | 2.24 | 1.20 |
| Gender (Ref: Male) |  |  |
| Female | 1.04 | 1.00 |
| Other | 2.22e+06 | 724.08 |
| Year of Birth (Ref: 1998-2005) |  |  |
| 1993-1998; Age 25-29 | 1.75 | 1.00 |
| 1983-1993; Age 30-39 | 1.73 | 1.00 |
| 1973-1983; Age 40-49 | 1.21 | 1.00 |
| 1963-1973; Age 50-59 | 1.49 | 1.00 |
| 1953-1963; Age 60-69 | 1.62 | 1.00 |
| 1943-1953; Age 70-79 | 1.37 | 1.00 |
| 1943 or Earlier; Age 80 or Older | 2.20 | 1.00 |
| Mother Absence/Presence (Ref: Present) |  |  |
| Absent | 2.03 | 1.00 |
| Father Absence/Presence (Ref: Present) |  |  |
| Absent | 1.83 | 1.00 |
| Childhood Religion (Ref: No Religion/Atheist/Agnostic) |  |  |
| Christianity | 2.95 | 1.58 |
| Islam | 5.53 | 2.33 |
| Some Other Religion | 2.64 | 1.23 |
| Race/Ethnicity (Ref: Ethnic Plurality) |  |  |
| Ethnic Minority | 2.21 | 1.39 |

**Table S22a: Nationally-Representative Descriptive Statistics of the Observed Sample (United States)**

| Variable | Proportion | Frequency |
| --- | --- | --- |
| Relationship with Mother |  |  |
| Very Good | 0.54 | 20590 |
| Somewhat Good | 0.30 | 11525 |
| Somewhat Bad | 0.09 | 3523 |
| Very Bad | 0.05 | 1874 |
| Not Applicable | 0.02 | 694 |
| Missing | 0.00 | 106 |
| Relationship with Father |  |  |
| Very Good | 0.40 | 15313 |
| Somewhat Good | 0.33 | 12666 |
| Somewhat Bad | 0.13 | 4879 |
| Very Bad | 0.07 | 2604 |
| Not Applicable | 0.07 | 2811 |
| Missing | 0.00 | 38 |
| Parent Marital Status |  |  |
| Married | 0.72 | 27415 |
| Divorced | 0.17 | 6325 |
| Never Married | 0.08 | 3048 |
| One or Both Had Died | 0.03 | 1024 |
| Missing | 0.01 | 500 |
| Childhood Income |  |  |
| Lived Comfortably | 0.39 | 15116 |
| Got By | 0.41 | 15682 |
| Found it Difficult | 0.13 | 5152 |
| Found it Very Difficult | 0.06 | 2342 |
| Missing | 0.00 | 19 |
| Childhood Abuse |  |  |
| Yes | 0.26 | 10026 |
| No | 0.73 | 28045 |
| Missing | 0.01 | 242 |
| Outsider |  |  |
| Yes | 0.27 | 10185 |
| No | 0.72 | 27714 |
| Not Applicable | 0.01 | 305 |
| Missing | 0.00 | 109 |
| Childhood Health |  |  |
| Excellent | 0.44 | 16866 |
| Very Good | 0.32 | 12108 |
| Good | 0.17 | 6444 |
| Fair | 0.06 | 2303 |
| Poor | 0.01 | 520 |
| Missing | 0.00 | 71 |
| Immigration Status |  |  |
| Born in This Country | 0.91 | 34865 |
| Born in Another Country | 0.08 | 3020 |
| Missing | 0.01 | 427 |
| Childhood Service Attendance |  |  |
| At Least 1/Week | 0.49 | 18609 |
| 1-3/Month | 0.17 | 6644 |
| <1/Month | 0.15 | 5829 |
| Never | 0.18 | 7085 |
| Missing | 0.00 | 145 |
| Gender |  |  |
| Male | 0.48 | 18222 |
| Female | 0.51 | 19562 |
| Other | 0.01 | 392 |
| Missing | 0.00 | 136 |
| Year of Birth |  |  |
| 1998-2005; Age 18-24 | 0.07 | 2682 |
| 1993-1998; Age 25-29 | 0.09 | 3540 |
| 1983-1993; Age 30-39 | 0.19 | 7284 |
| 1973-1983; Age 40-49 | 0.15 | 5649 |
| 1963-1973; Age 50-59 | 0.18 | 6745 |
| 1953-1963; Age 60-69 | 0.18 | 6832 |
| 1943-1953; Age 70-79 | 0.11 | 4054 |
| 1943 or Earlier; 80 or Older | 0.04 | 1525 |
| Missing | . | . |
| Childhood Religion |  |  |
| Christianity | 0.79 | 30444 |
| Islam | 0.01 | 220 |
| Hinduism | 0.01 | 203 |
| Buddhism | 0.00 | 172 |
| Judaism | 0.02 | 787 |
| Sikhism | 0.00 | 47 |
| Baha'i | 0.00 | 4 |
| Jainism | 0.00 | 18 |
| Shinto | 0.00 | 6 |
| Taoism | 0.00 | 17 |
| Confucianism | 0.00 | 8 |
| Primal, Animist, or Folk Religion | 0.00 | 67 |
| Spiritism | . | . |
| African-Derived | . | . |
| Chinese | . | . |
| Some Other Religion | 0.01 | 359 |
| No Religion/Atheist/Agnostic | 0.15 | 5845 |
| Missing | 0.00 | 115 |
| Race/Ethnicity |  |  |
| White | 0.62 | 23605 |
| Other | 0.03 | 997 |
| Black | 0.12 | 4501 |
| Asian | 0.06 | 2466 |
| Hispanic | 0.18 | 6724 |
| Other | . | . |
| Missing | 0.00 | 20 |

**Table S22b: Variations Across Childhood Predictors (United States)**

| Variable | IRR | SE | Prob | LCI | UCI | Global p-value |
| --- | --- | --- | --- | --- | --- | --- |
| Relationship with Mother (Ref: Very/Somewhat Bad) |  |  |  |  |  |  |
| Very/Somewhat Good | 1.06 | 0.05 | 0.20 | 0.97 | 1.17 | 0.20 |
| Relationship with Father (Ref: Very/Somewhat Bad) |  |  |  |  |  |  |
| Very/Somewhat Good | 1.07 | 0.04 | 0.12 | 0.98 | 1.15 | 0.12 |
| Parent Marital Status (Ref: Married) |  |  |  |  |  |  |
| Divorced | 1.03 | 0.05 | 0.57 | 0.94 | 1.12 | 0.11 |
| Never Married | 1.20 | 0.10 | 0.02 | 1.02 | 1.41 | . |
| One or Both Had Died | 1.13 | 0.11 | 0.22 | 0.93 | 1.37 | . |
| Childhood Income (Ref: Got By) |  |  |  |  |  |  |
| Lived Comfortably | 0.98 | 0.03 | 0.52 | 0.93 | 1.04 | 0.00 |
| Found it Difficult | 1.10 | 0.05 | 0.03 | 1.01 | 1.20 | . |
| Found it Very Difficult | 1.18 | 0.07 | 0.01 | 1.04 | 1.33 | . |
| Childhood Abuse (Ref: No) |  |  |  |  |  |  |
| Yes | 1.03 | 0.03 | 0.38 | 0.97 | 1.10 | 0.38 |
| Outsider (Ref: No) |  |  |  |  |  |  |
| Yes | 0.85 | 0.03 | 0.00 | 0.79 | 0.92 | 0.00 |
| Childhood Health (Ref: Good) |  |  |  |  |  |  |
| Excellent | 1.05 | 0.05 | 0.26 | 0.96 | 1.14 | 0.10 |
| Very Good | 1.04 | 0.05 | 0.39 | 0.95 | 1.14 | . |
| Fair | 0.99 | 0.10 | 0.92 | 0.81 | 1.21 | . |
| Poor | 1.50 | 0.22 | 0.01 | 1.12 | 2.00 | . |
| Immigration Status (Ref: Born in This Country) |  |  |  |  |  |  |
| Born in Another Country | 0.96 | 0.06 | 0.54 | 0.86 | 1.08 | 0.54 |
| Childhood Service Attendance (Ref: Never) |  |  |  |  |  |  |
| At Least 1/Week | 1.71 | 0.10 | 0.00 | 1.52 | 1.91 | 0.00 |
| 1-3/Month | 1.27 | 0.08 | 0.00 | 1.12 | 1.44 | . |
| <1/Month | 1.33 | 0.09 | 0.00 | 1.16 | 1.53 | . |
| Gender (Ref: Male) |  |  |  |  |  |  |
| Female | 1.29 | 0.03 | 0.00 | 1.23 | 1.36 | 0.00 |
| Other | 1.03 | 0.36 | 0.94 | 0.52 | 2.04 | . |
| Year of Birth (Ref: 1998-2005) |  |  |  |  |  |  |
| 1993-1998; Age 25-29 | 1.22 | 0.19 | 0.22 | 0.89 | 1.66 | 0.00 |
| 1983-1993; Age 30-39 | 1.25 | 0.18 | 0.11 | 0.95 | 1.65 | . |
| 1973-1983; Age 40-49 | 1.51 | 0.21 | 0.00 | 1.15 | 1.98 | . |
| 1963-1973; Age 50-59 | 1.83 | 0.25 | 0.00 | 1.40 | 2.39 | . |
| 1953-1963; Age 60-69 | 1.91 | 0.26 | 0.00 | 1.47 | 2.50 | . |
| 1943-1953; Age 70-79 | 1.82 | 0.25 | 0.00 | 1.39 | 2.37 | . |
| 1943 or Earlier; Age 80 or Older | 2.04 | 0.29 | 0.00 | 1.54 | 2.69 | . |
| Mother Absence/Presence (Ref: Present) |  |  |  |  |  |  |
| Absent | 1.01 | 0.09 | 0.93 | 0.85 | 1.20 | 0.93 |
| Father Absence/Presence (Ref: Present) |  |  |  |  |  |  |
| Absent | 0.96 | 0.07 | 0.58 | 0.83 | 1.11 | 0.58 |
| Childhood Religion (Ref: No Religion/Atheist/Agnostic) |  |  |  |  |  |  |
| Christianity | 2.09 | 0.18 | 0.00 | 1.77 | 2.46 | 0.00 |
| Some Other Religion | 1.56 | 0.18 | 0.00 | 1.24 | 1.97 | . |
| Race/Ethnicity (Ref: Ethnic Plurality) |  |  |  |  |  |  |
| Ethnic Minority | 1.17 | 0.03 | 0.00 | 1.11 | 1.24 | 0.00 |

**Table S22c: E-Values and E-Value Limits (United States)**

| Variable | E-Value | E-Value Limit |
| --- | --- | --- |
| Relationship with Mother (Ref: Very/Somewhat Bad) |  |  |
| Very/Somewhat Good | 1.27 | 1.00 |
| Relationship with Father (Ref: Very/Somewhat Bad) |  |  |
| Very/Somewhat Good | 1.28 | 1.00 |
| Parent Marital Status (Ref: Married) |  |  |
| Divorced | 1.16 | 1.00 |
| Never Married | 1.57 | 1.12 |
| One or Both Had Died | 1.43 | 1.00 |
| Childhood Income (Ref: Got By) |  |  |
| Lived Comfortably | 1.13 | 1.00 |
| Found it Difficult | 1.36 | 1.08 |
| Found it Very Difficult | 1.53 | 1.17 |
| Childhood Abuse (Ref: No) |  |  |
| Yes | 1.17 | 1.00 |
| Outsider (Ref: No) |  |  |
| Yes | 1.51 | 1.24 |
| Childhood Health (Ref: Good) |  |  |
| Excellent | 1.24 | 1.00 |
| Very Good | 1.21 | 1.00 |
| Fair | 1.10 | 1.00 |
| Poor | 2.07 | 1.30 |
| Immigration Status (Ref: Born in This Country) |  |  |
| Born in Another Country | 1.20 | 1.00 |
| Childhood Service Attendance (Ref: Never) |  |  |
| At Least 1/Week | 2.39 | 1.77 |
| 1-3/Month | 1.70 | 1.30 |
| <1/Month | 1.80 | 1.36 |
| Gender (Ref: Male) |  |  |
| Female | 1.73 | 1.45 |
| Other | 1.16 | 1.00 |
| Year of Birth (Ref: 1998-2005) |  |  |
| 1993-1998; Age 25-29 | 1.60 | 1.00 |
| 1983-1993; Age 30-39 | 1.67 | 1.00 |
| 1973-1983; Age 40-49 | 2.09 | 1.35 |
| 1963-1973; Age 50-59 | 2.57 | 1.65 |
| 1953-1963; Age 60-69 | 2.68 | 1.72 |
| 1943-1953; Age 70-79 | 2.55 | 1.64 |
| 1943 or Earlier; Age 80 or Older | 2.86 | 1.79 |
| Mother Absence/Presence (Ref: Present) |  |  |
| Absent | 1.08 | 1.00 |
| Father Absence/Presence (Ref: Present) |  |  |
| Absent | 1.21 | 1.00 |
| Childhood Religion (Ref: No Religion/Atheist/Agnostic) |  |  |
| Christianity | 2.92 | 1.99 |
| Some Other Religion | 2.17 | 1.47 |
| Race/Ethnicity (Ref: Ethnic Plurality) |  |  |
| Ethnic Minority | 1.51 | 1.29 |

**Table S23: Population Weighted Meta-Analysis of Regression Results**

| Variable | Category | RR | 95% CI |
| --- | --- | --- | --- |
| Relationship with mother | (Ref: Very bad/somewhat bad) |  |  |
|  | Very/somewhat good | 1.03 | (0.96,1.11) |
| Relationship with father | (Ref: Very bad/somewhat bad) |  |  |
|  | Very/somewhat good | 1.00 | (0.94,1.07) |
| Parent marital status | (Ref: Parents married) |  |  |
|  | No, divorced | 0.96 | (0.87,1.06) |
|  | Single, never married | 1.05 | (0.92,1.19) |
|  | No, one or both had died | 0.89 | (0.72,1.10) |
| Subjective financial status of family growing up | (Ref: Got by) |  |  |
|  | Lived comfortably | 1.03 | (0.99,1.08) |
|  | Found it difficult | 1.01 | (0.94,1.08) |
|  | Found it very difficult | 0.98 | (0.86,1.11) |
| Abuse | (Ref: No) |  |  |
|  | Yes | 1.08 | (1.03,1.14) |
| Outsider growing up | (Ref: No) |  |  |
|  | Yes | 1.07 | (1.02,1.12) |
| Self-rated health growing up | (Ref: Good) |  |  |
|  | Excellent | 1.06 | (1.00,1.13) |
|  | Very good | 1.01 | (0.95,1.08) |
|  | Fair | 0.91 | (0.82,1.02) |
|  | Poor | 1.06 | (0.91,1.25) |
| Immigration status | (Ref: Born in this country) |  |  |
|  | No | 1.02 | (0.93,1.12) |
| Age 12 religious service attendance | (Ref: Never) |  |  |
|  | At least 1/week | 1.96 | (1.76,2.17) |
|  | 1-3/month | 1.71 | (1.54,1.90) |
|  | Less than 1/month | 1.25 | (1.12,1.40) |
| Year of birth | (Ref: 1998-2005; age 18-24) |  |  |
|  | 1993-1998; age 25-29 | 1.08 | (0.97,1.20) |
|  | 1983-1993; age 30-39 | 1.09 | (0.99,1.20) |
|  | 1973-1983; age 40-49 | 1.15 | (1.04,1.26) |
|  | 1963-1973; age 50-59 | 1.24 | (1.13,1.36) |
|  | 1953-1963; age 60-69 | 1.24 | (1.12,1.37) |
|  | 1943-1953; age 70-79 | 1.26 | (1.10,1.44) |
|  | 1943 or earlier; age 80+ | 1.36 | (0.90,2.06) |
| Gender | (Ref: Male) |  |  |
|  | Female | 1.09 | (1.05,1.14) |
|  | Other | 0.00 | (0.00,0.01) |

**Table S24: Population Weighted Meta-Analysis of E-Values**

| Variable | Category | E-value | E-value Limit |
| --- | --- | --- | --- |
| Relationship with mother | (Ref: Very bad/somewhat bad) |  |  |
|  | Very/somewhat good | 1.21 | 1.00 |
| Relationship with father | (Ref: Very bad/somewhat bad) |  |  |
|  | Very/somewhat good | 1.06 | 1.00 |
| Parent marital status | (Ref: Parents married) |  |  |
|  | No, divorced | 1.24 | 1.00 |
|  | Single, never married | 1.28 | 1.00 |
|  | No, one or both had died | 1.49 | 1.00 |
| Subjective financial status of family growing up | (Ref: Got by) |  |  |
|  | Lived comfortably | 1.21 | 1.00 |
|  | Found it difficult | 1.11 | 1.00 |
|  | Found it very difficult | 1.17 | 1.00 |
| Abuse | (Ref: No) |  |  |
|  | Yes | 1.37 | 1.20 |
| Outsider growing up | (Ref: No) |  |  |
|  | Yes | 1.34 | 1.18 |
| Self-rated health growing up | (Ref: Good) |  |  |
|  | Excellent | 1.31 | 1.00 |
|  | Very good | 1.13 | 1.00 |
|  | Fair | 1.42 | 1.00 |
|  | Poor | 1.33 | 1.00 |
| Immigration status | (Ref: Born in this country) |  |  |
|  | No | 1.16 | 1.00 |
| Age 12 religious service attendance | (Ref: Never) |  |  |
|  | At least 1/week | 3.32 | 2.93 |
|  | 1-3/month | 2.82 | 2.46 |
|  | Less than 1/month | 1.82 | 1.49 |
| Year of birth | (Ref: 1998-2005; age 18-24) |  |  |
|  | 1993-1998; age 25-29 | 1.36 | 1.00 |
|  | 1983-1993; age 30-39 | 1.40 | 1.00 |
|  | 1973-1983; age 40-49 | 1.55 | 1.25 |
|  | 1963-1973; age 50-59 | 1.78 | 1.50 |
|  | 1953-1963; age 60-69 | 1.77 | 1.48 |
|  | 1943-1953; age 70-79 | 1.82 | 1.42 |
|  | 1943 or earlier; age 80+ | 2.06 | 1.00 |
| Gender | (Ref: Male) |  |  |
|  | Female | 1.42 | 1.29 |
|  | Other | 685.37 | 364.80 |


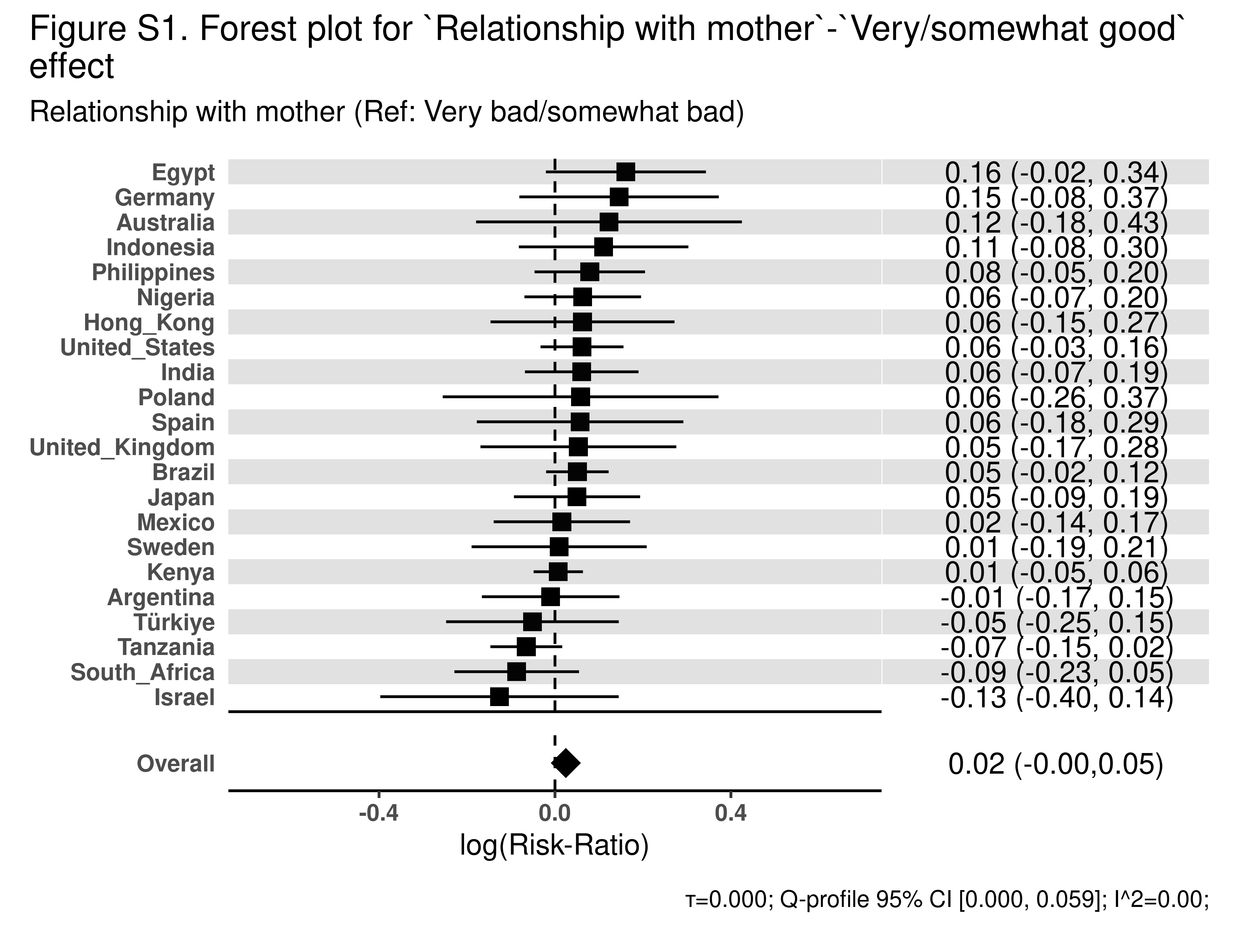

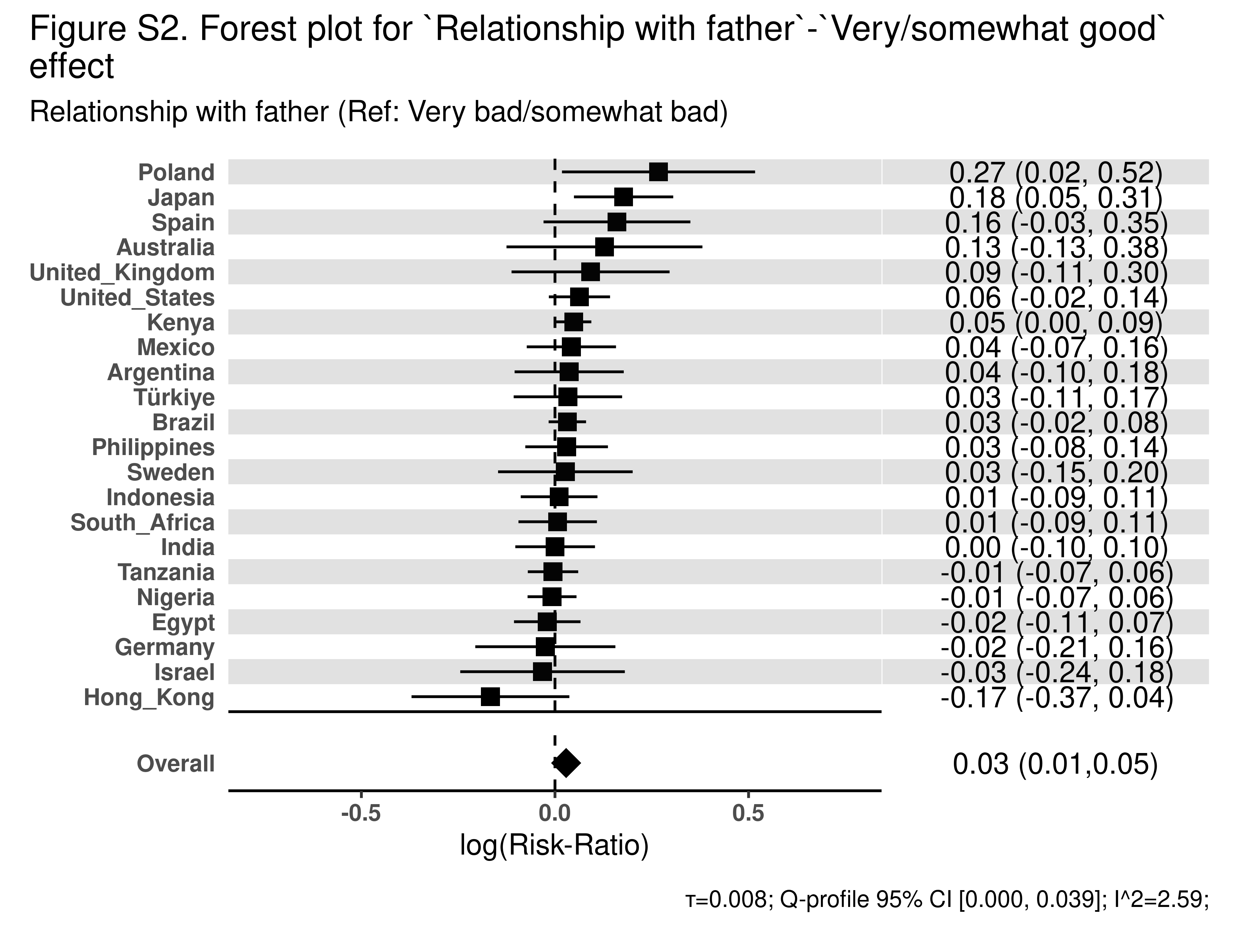

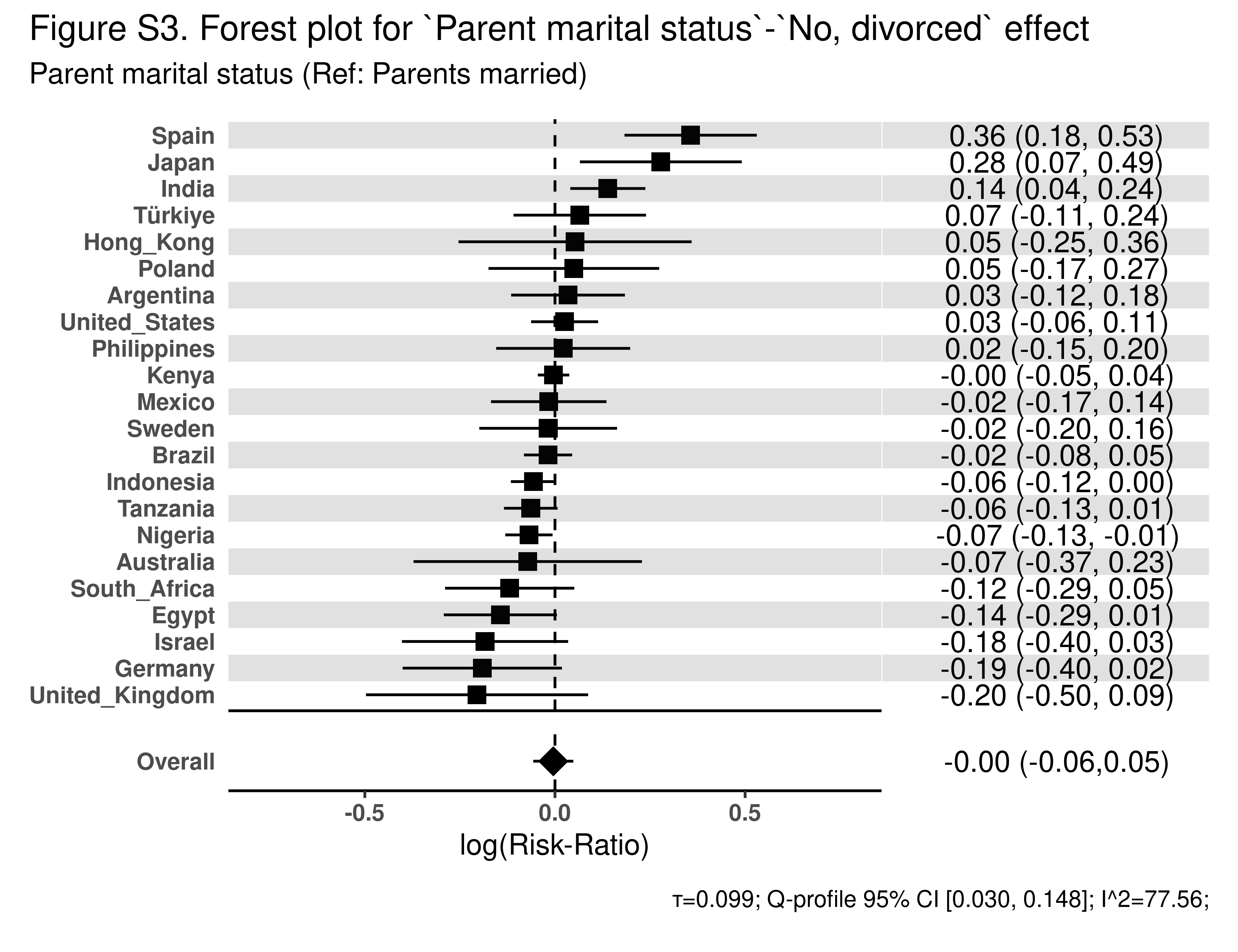

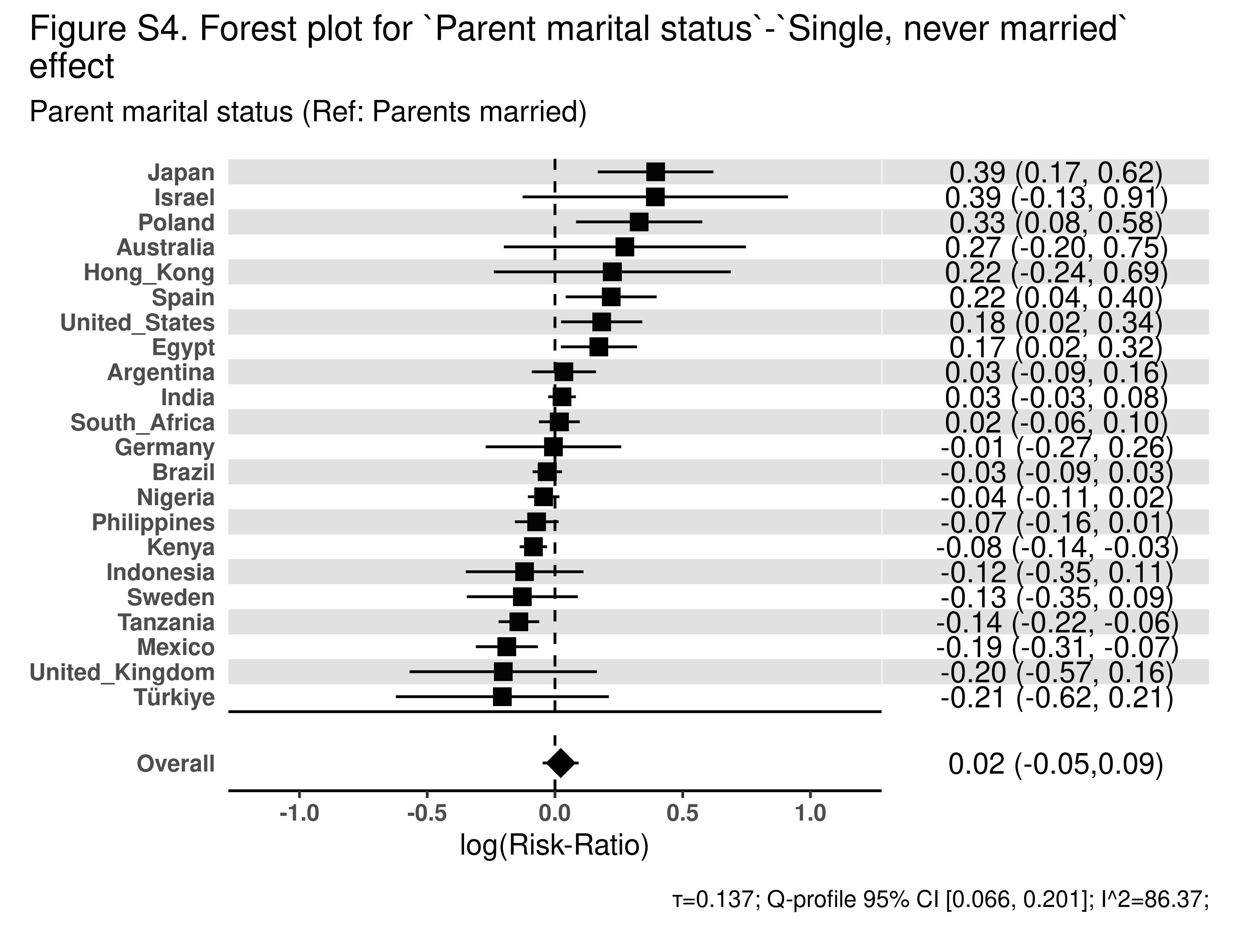

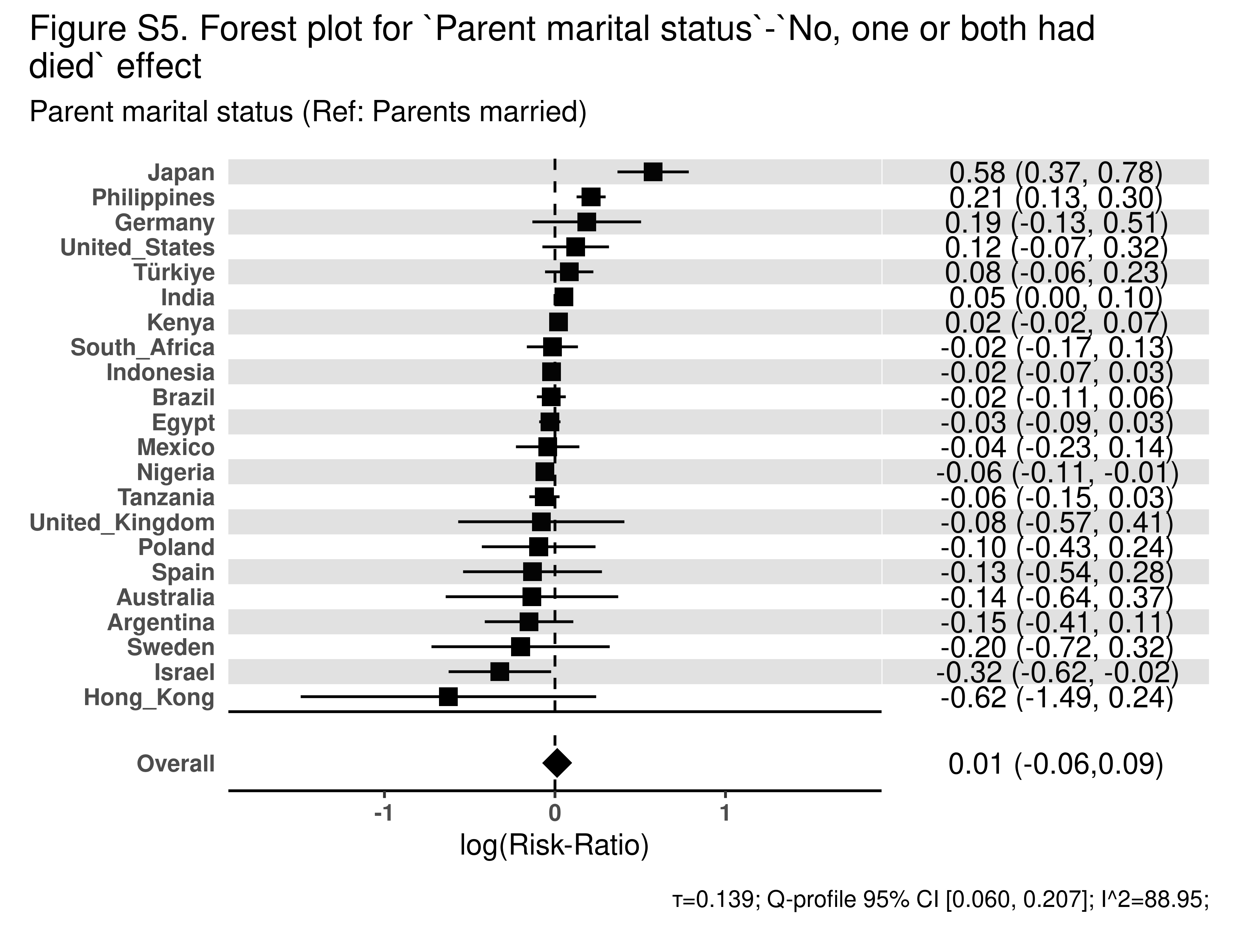

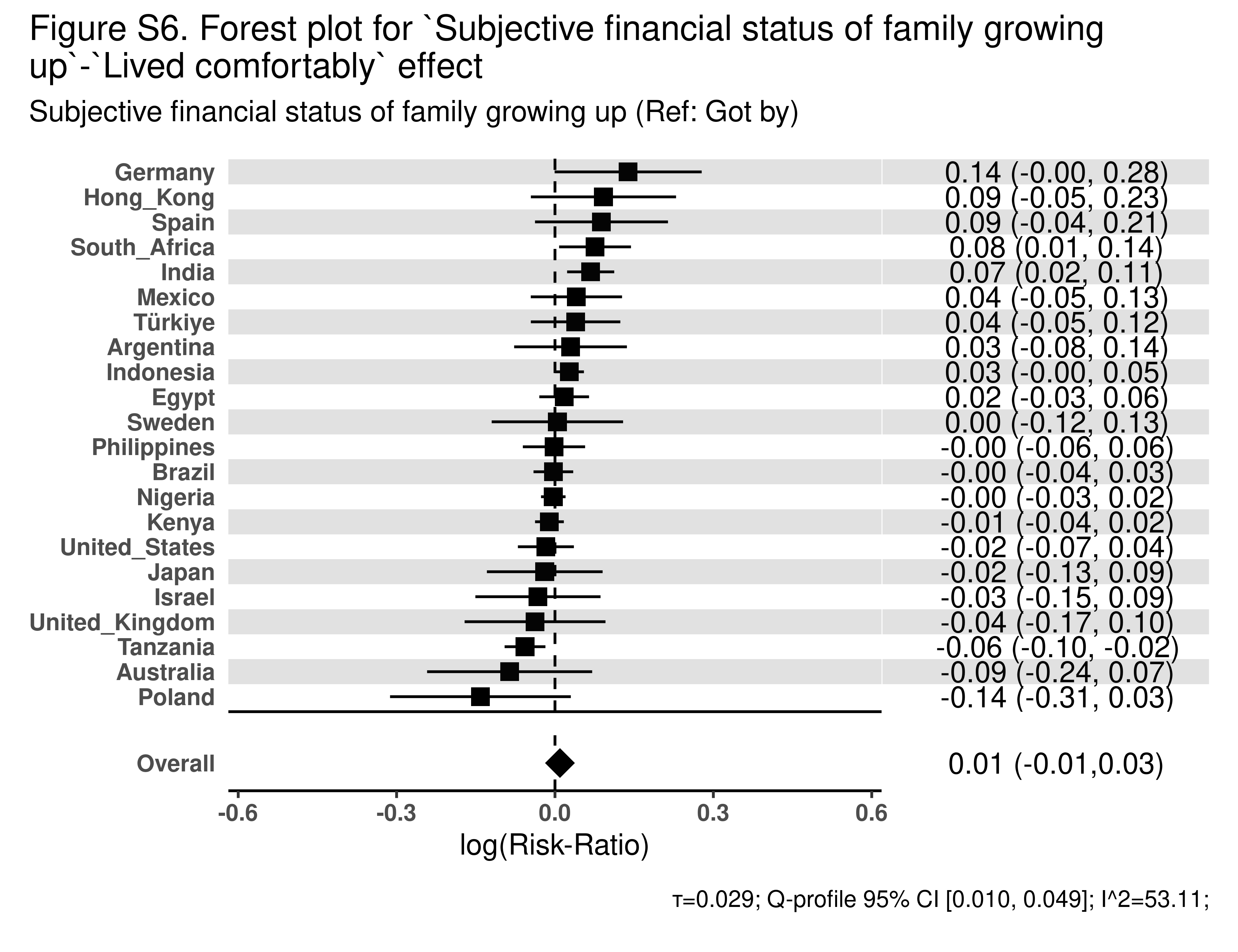

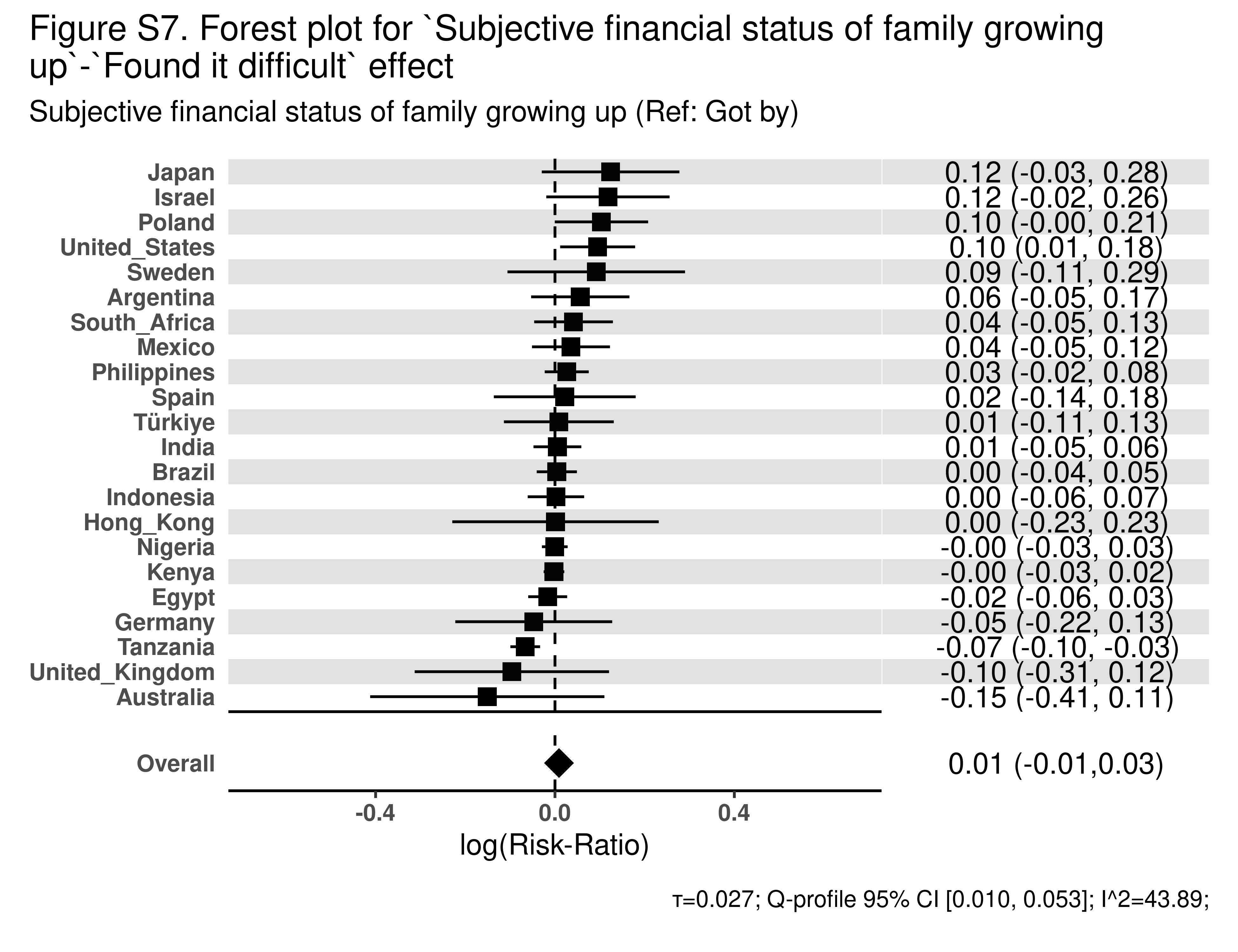

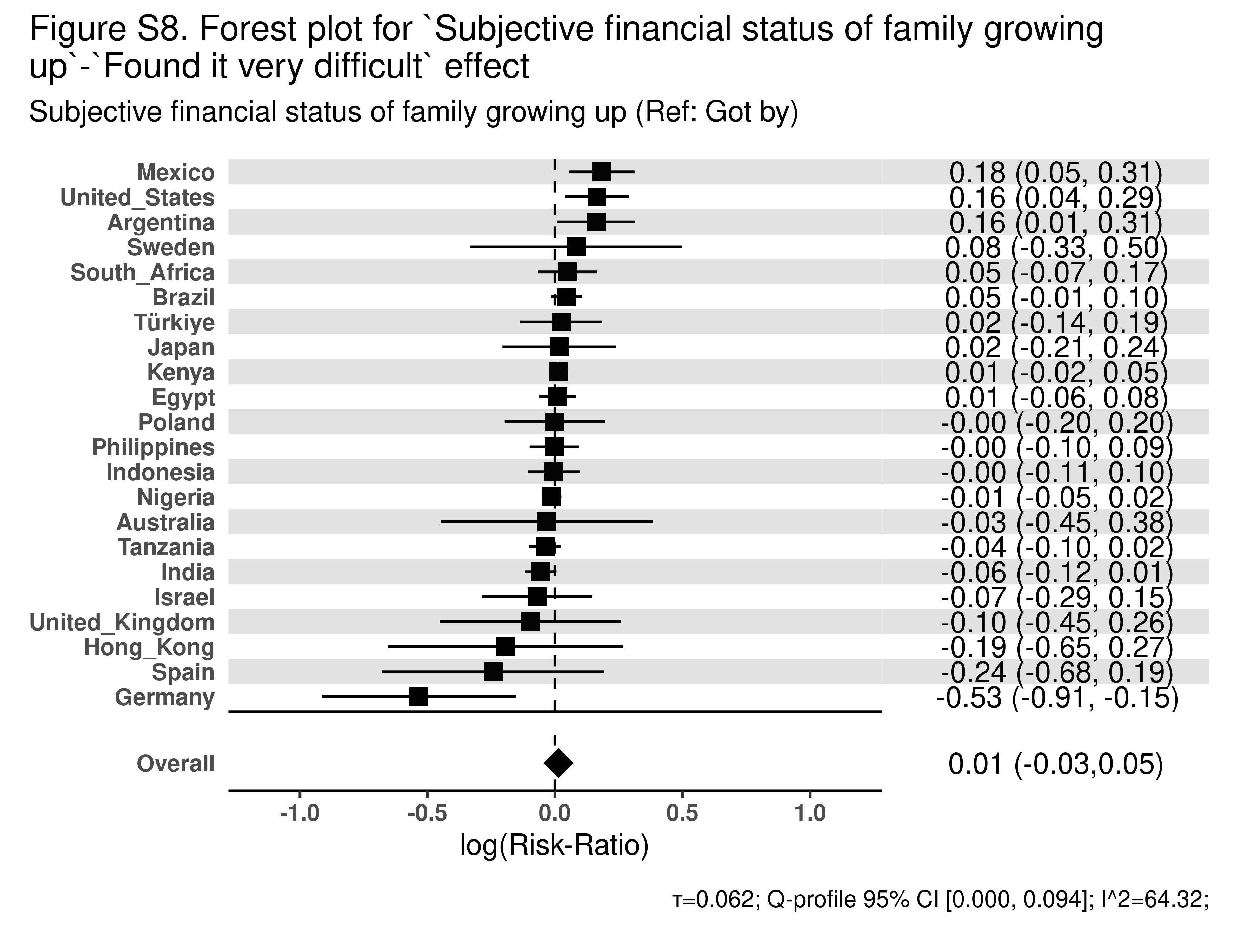

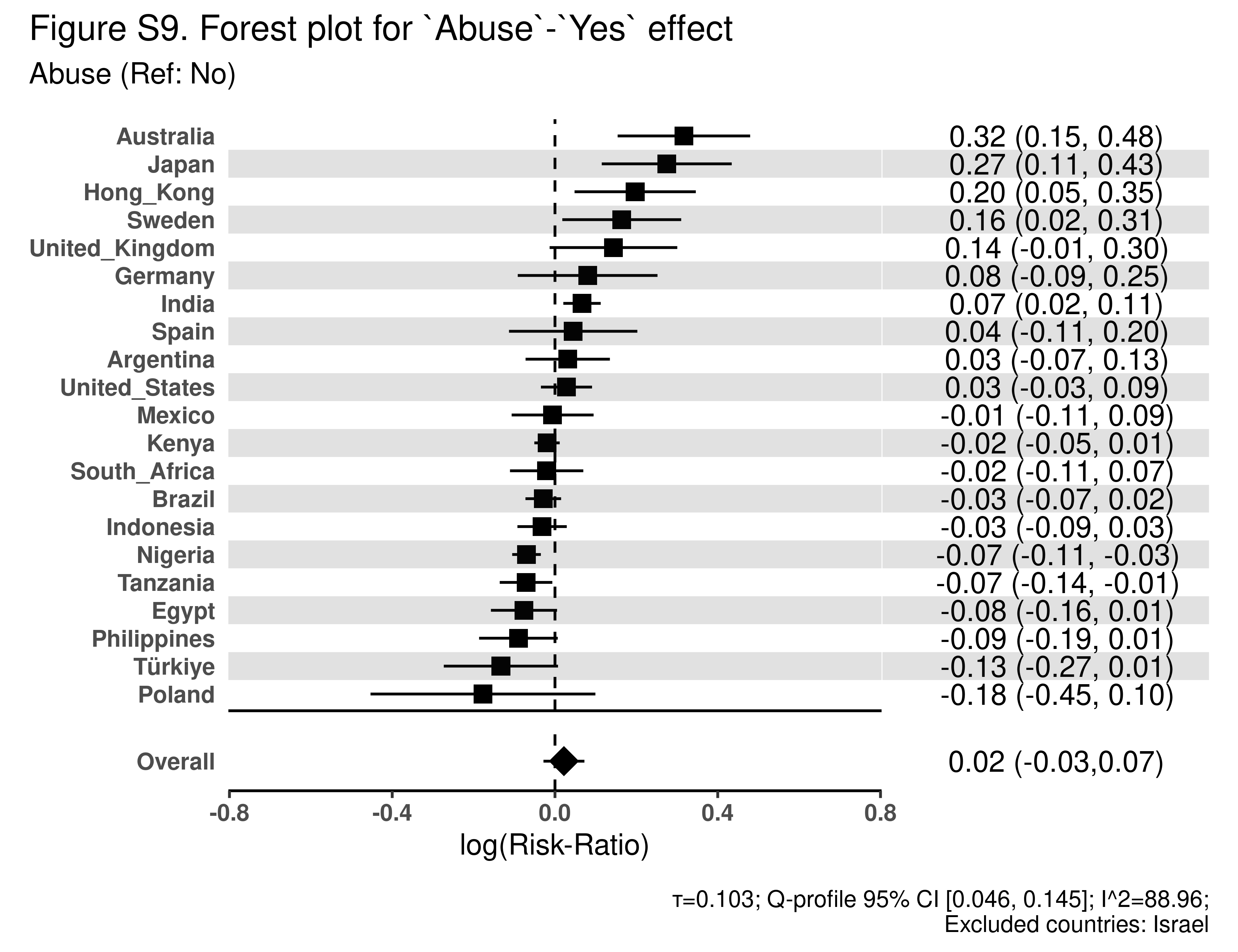

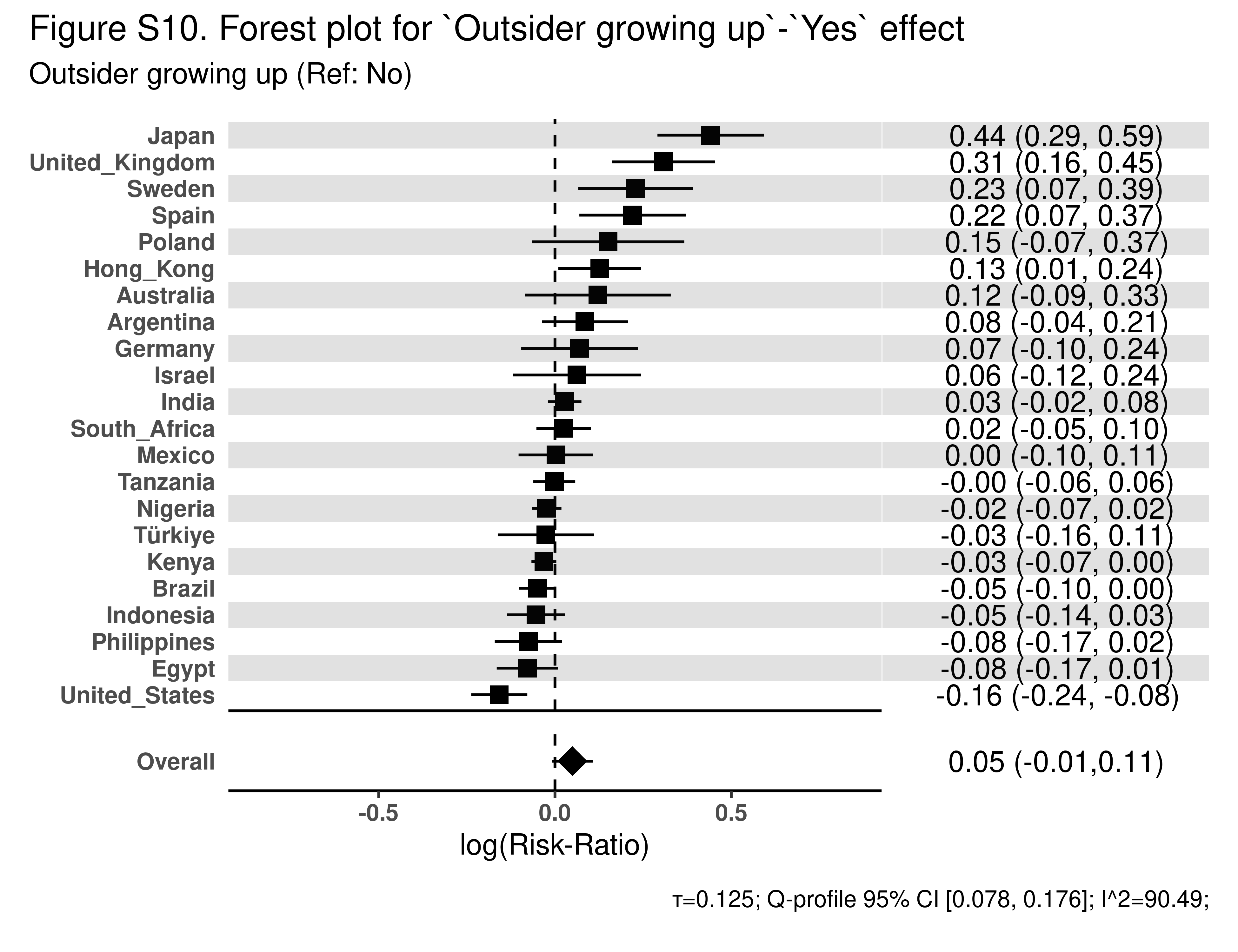

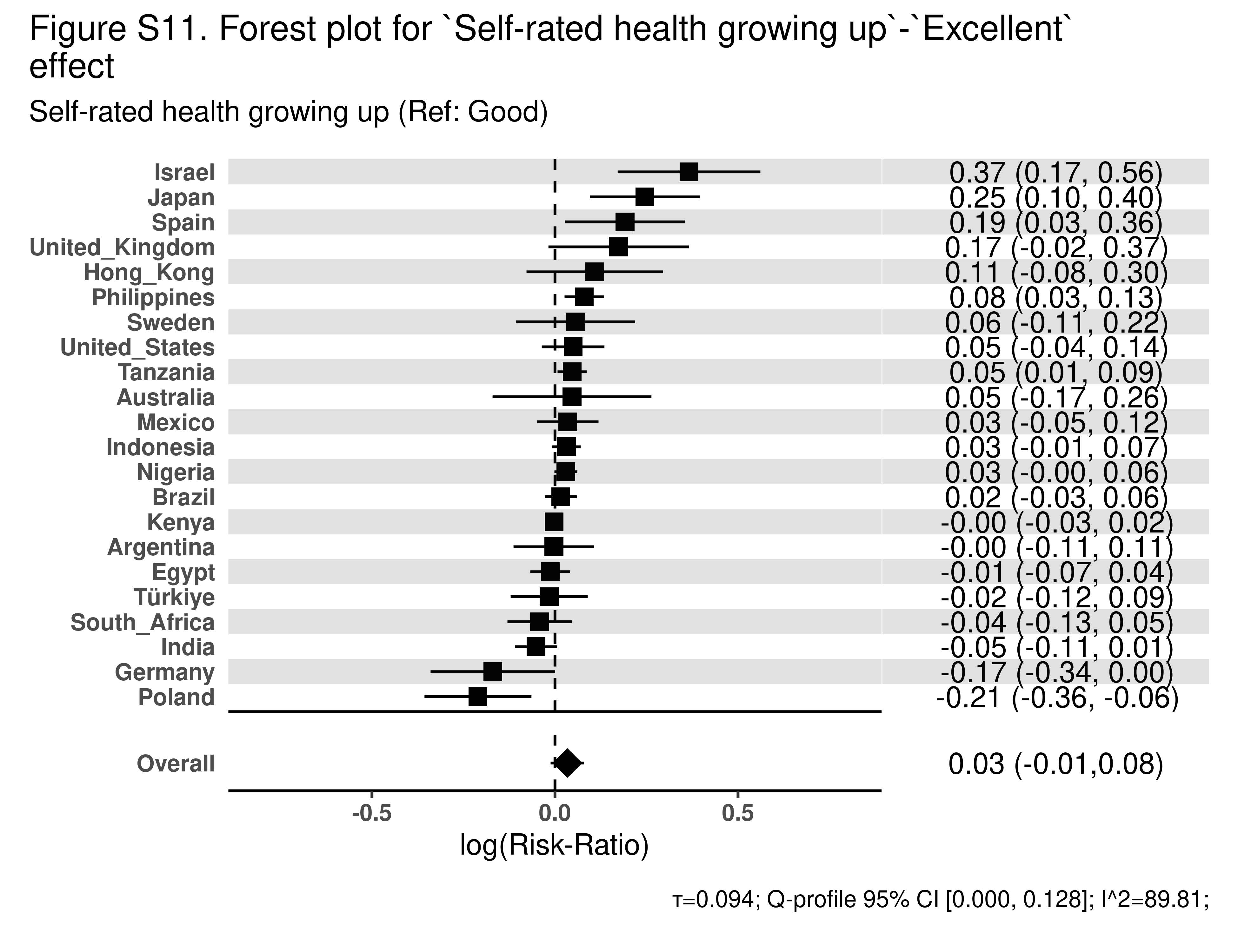

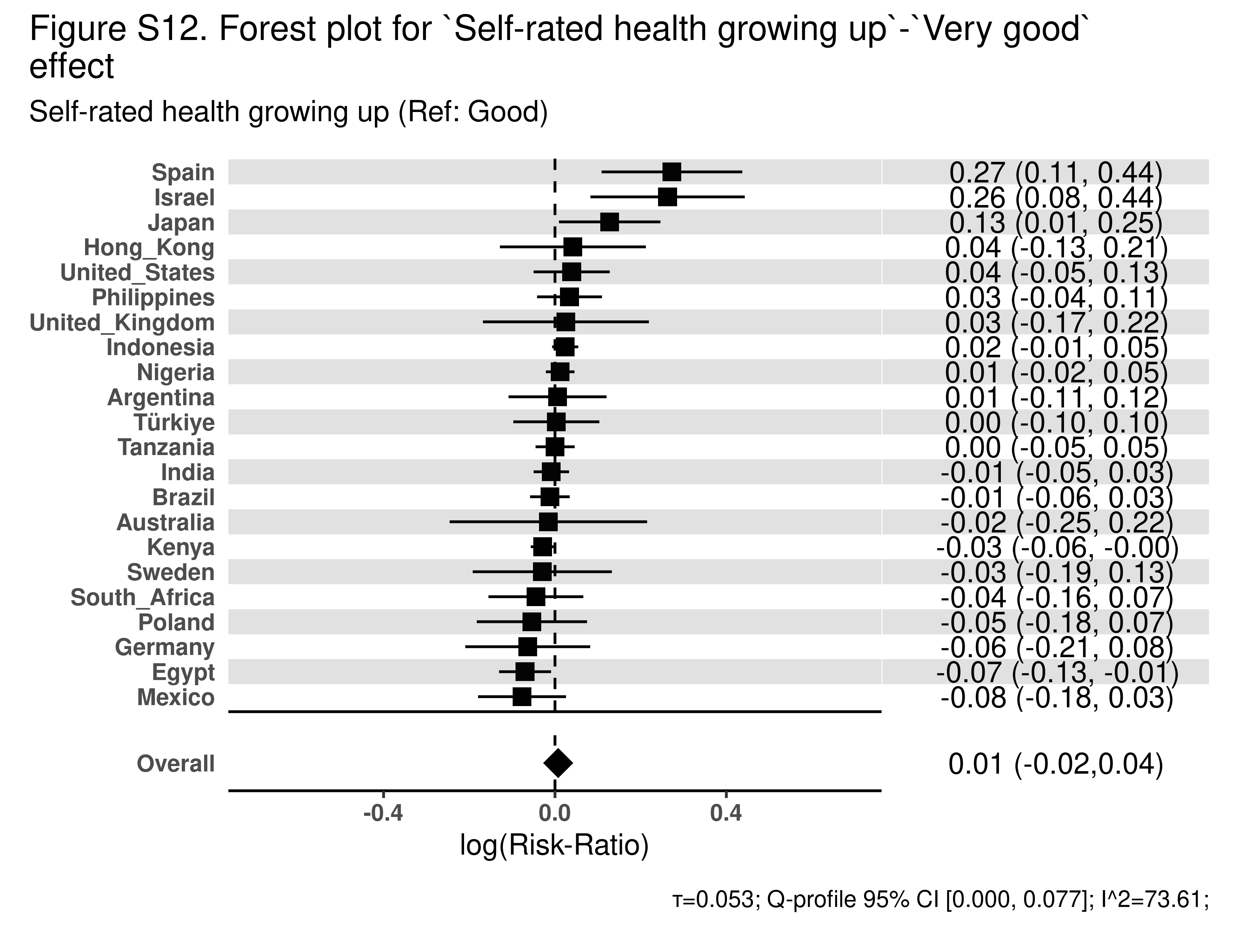

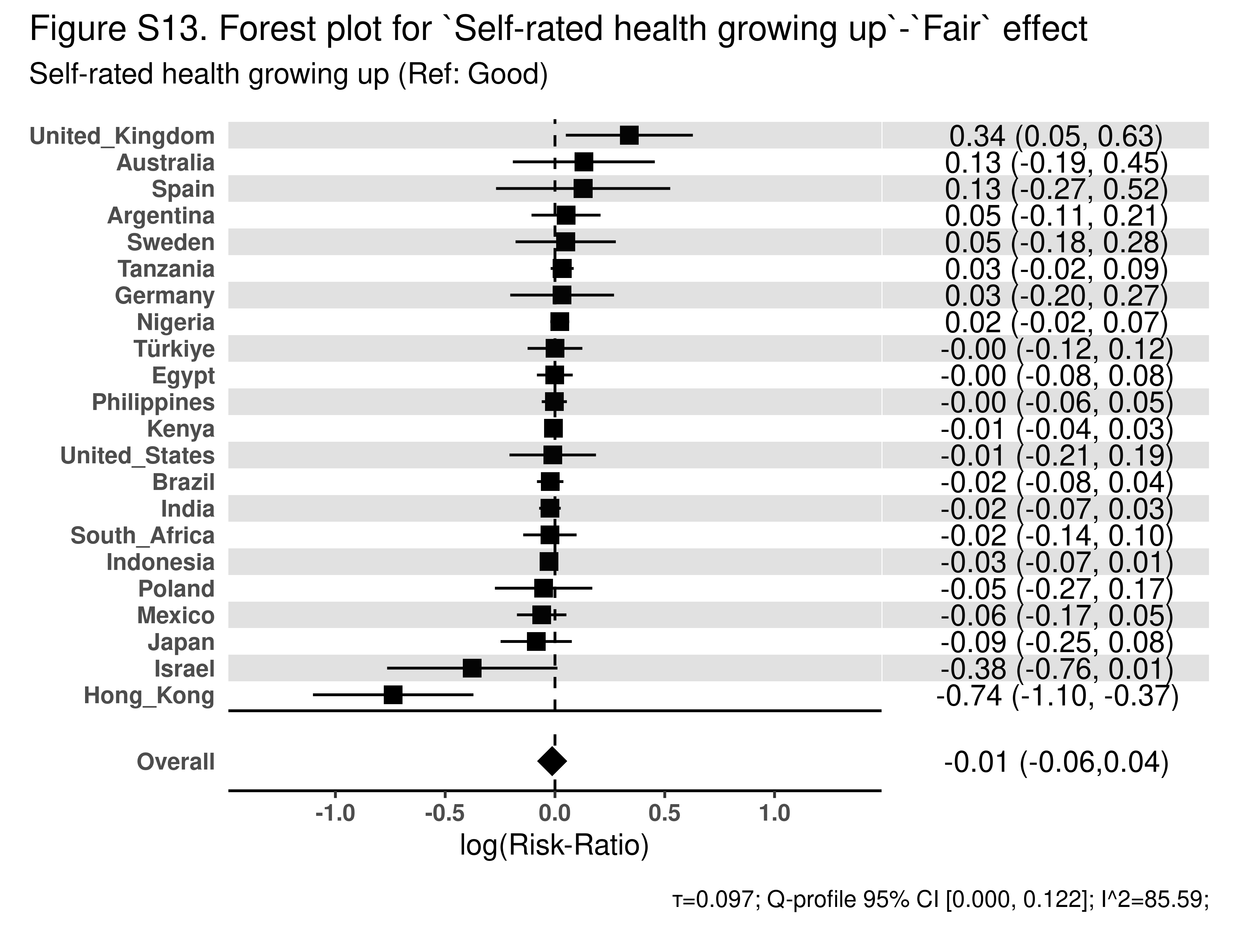

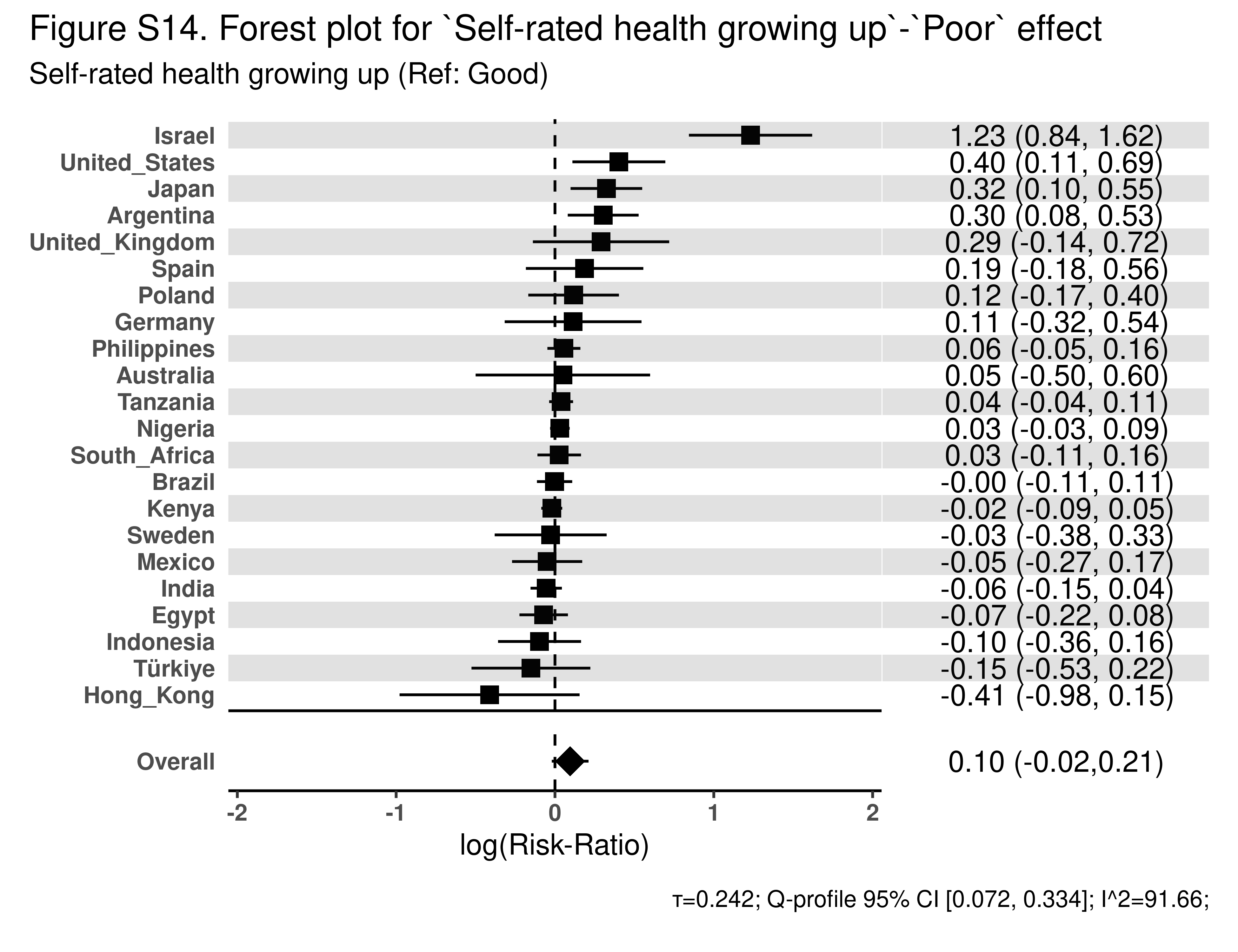

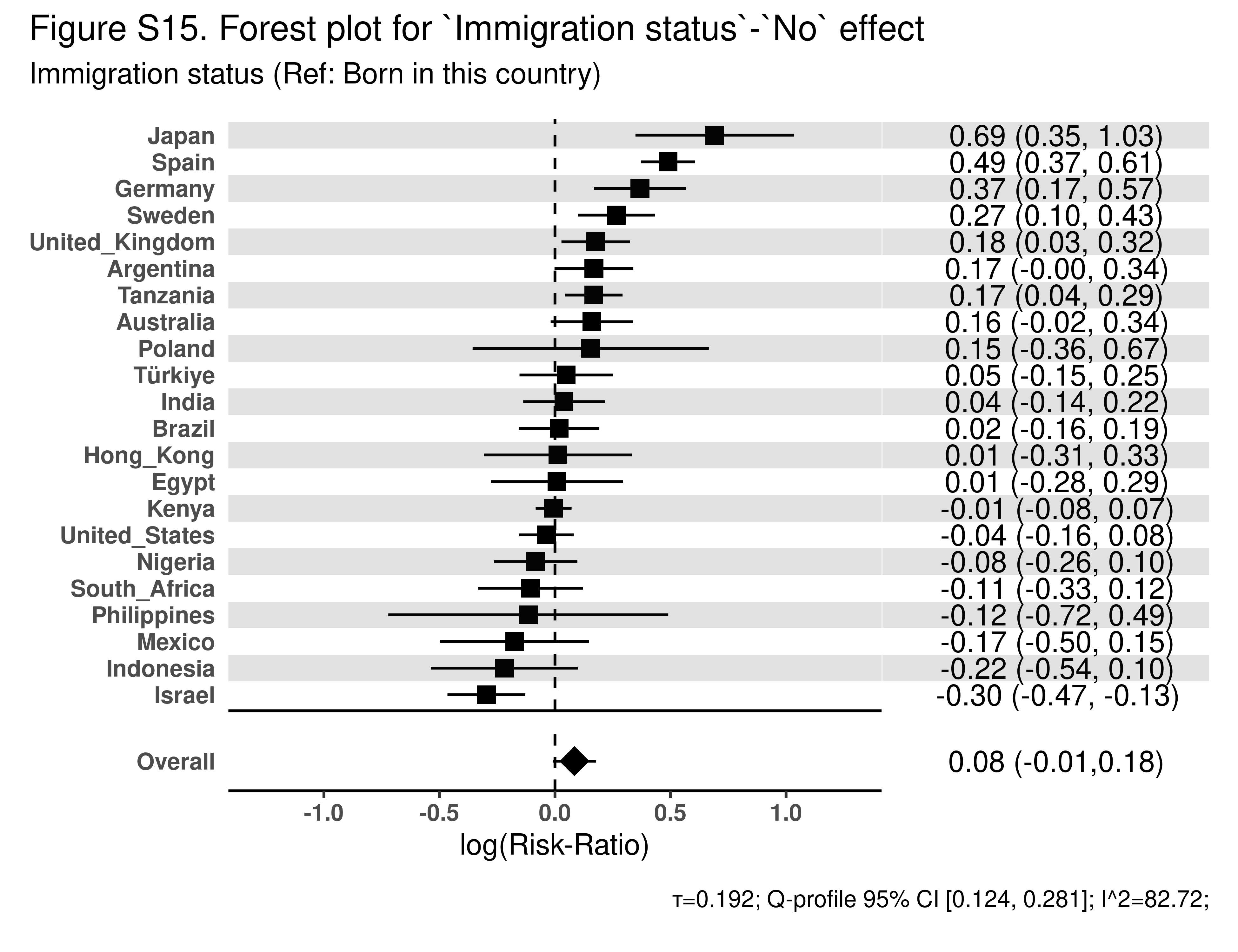

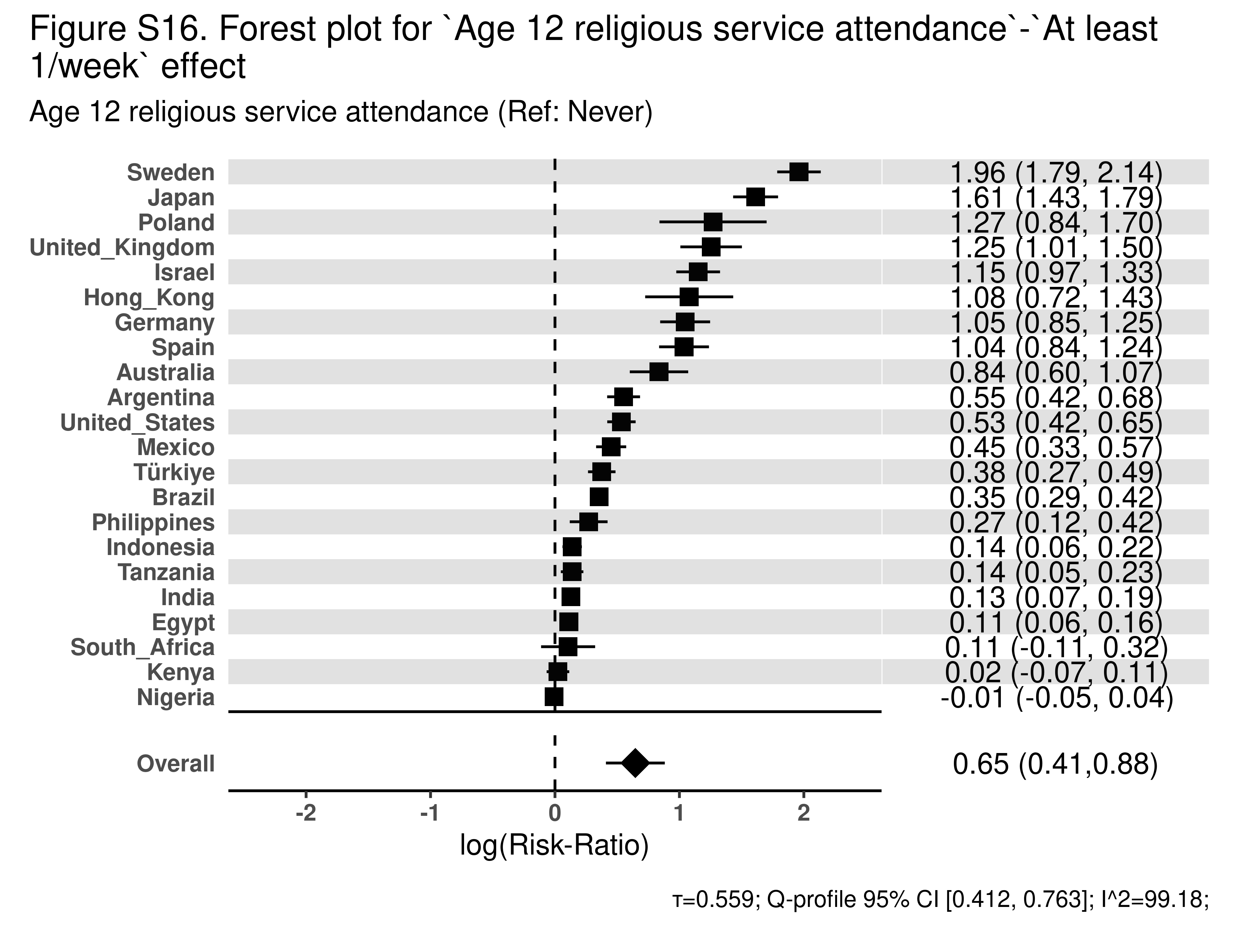

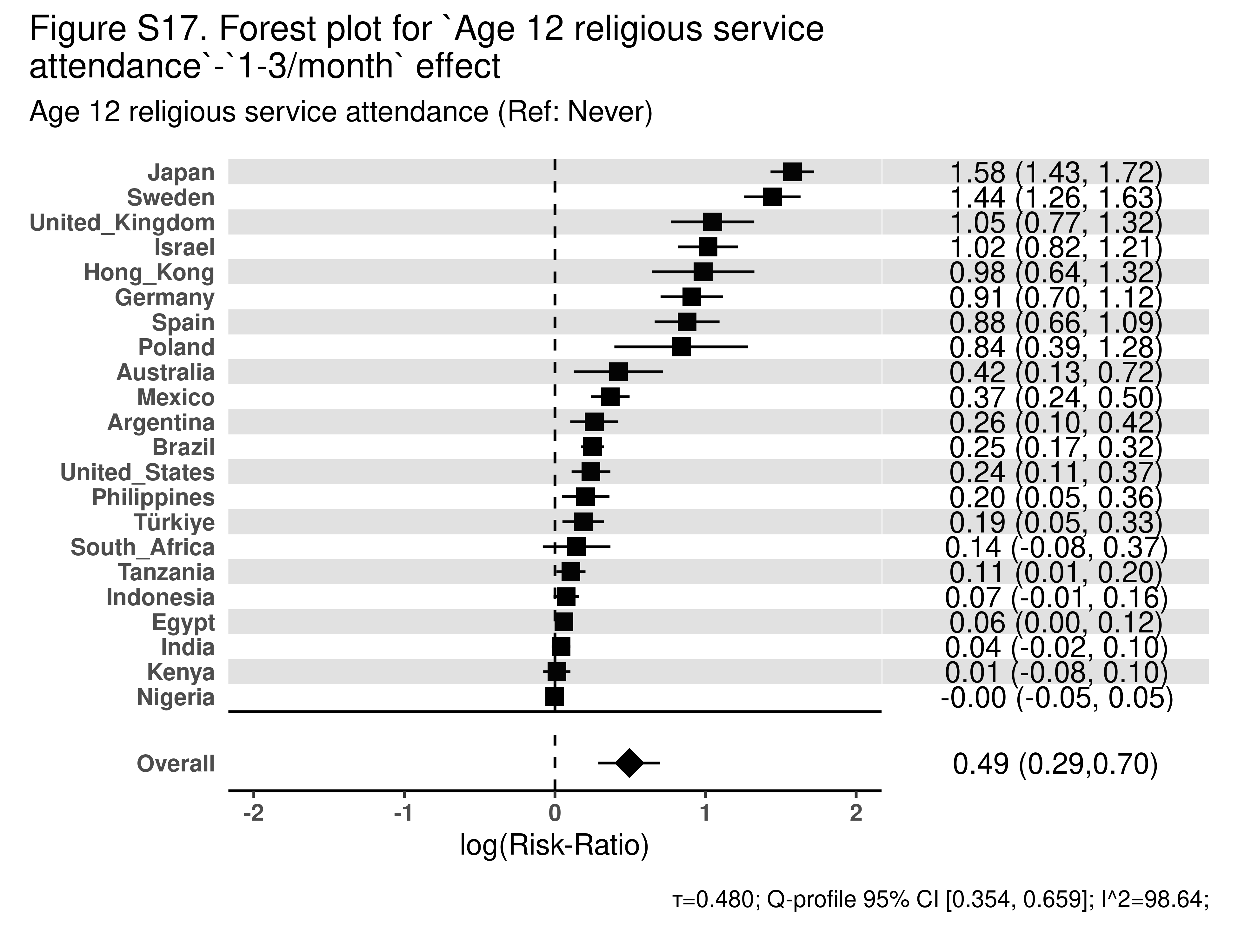

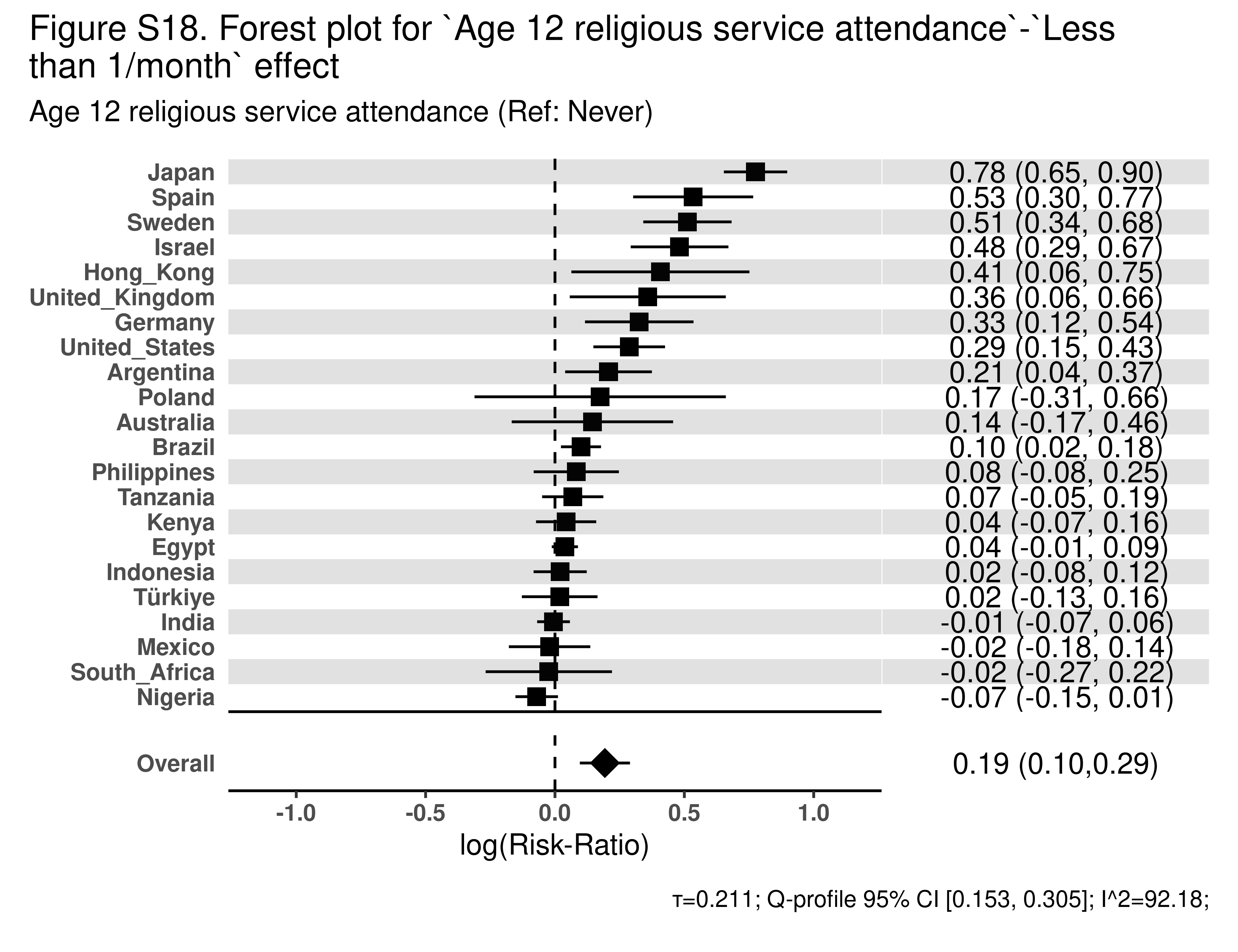

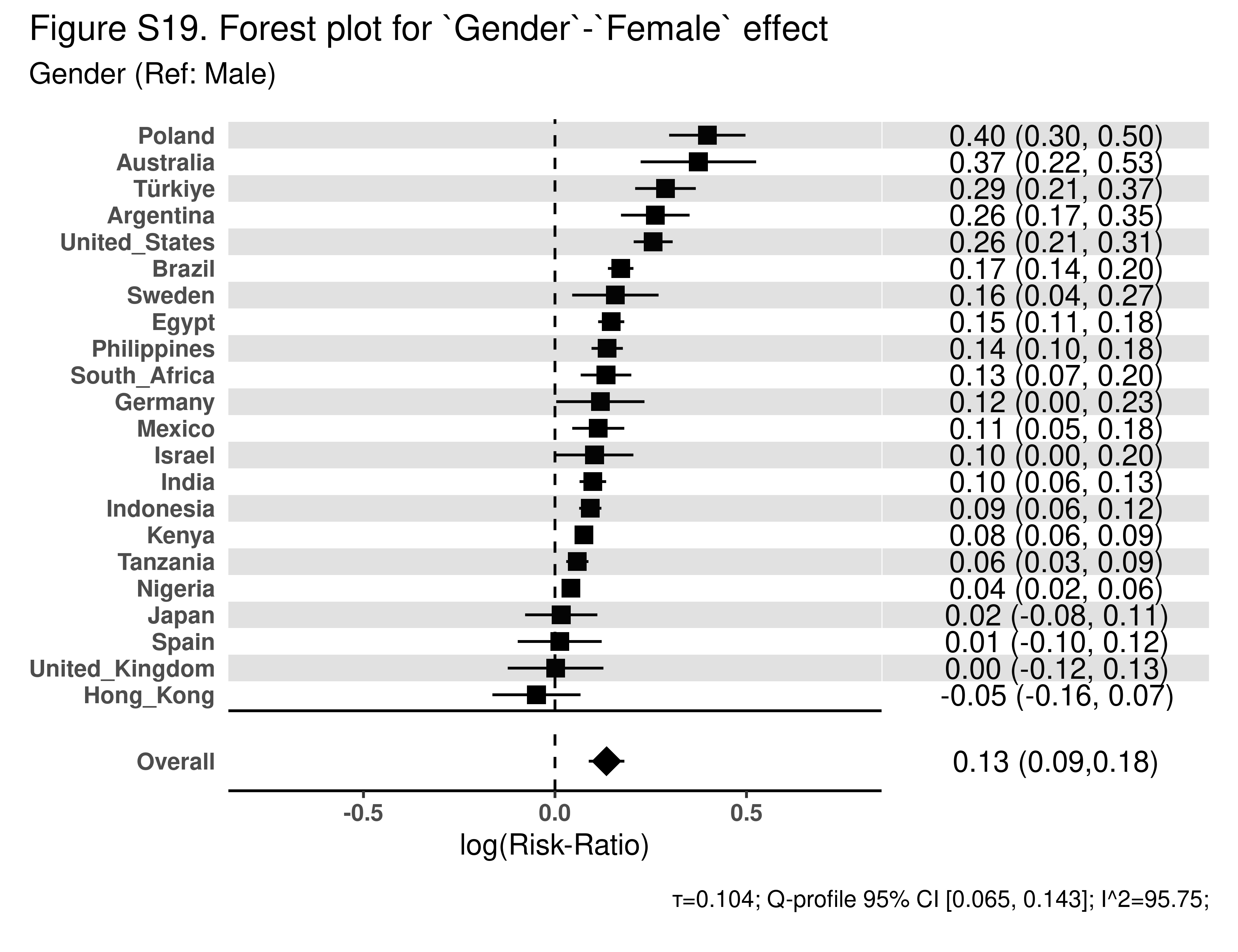

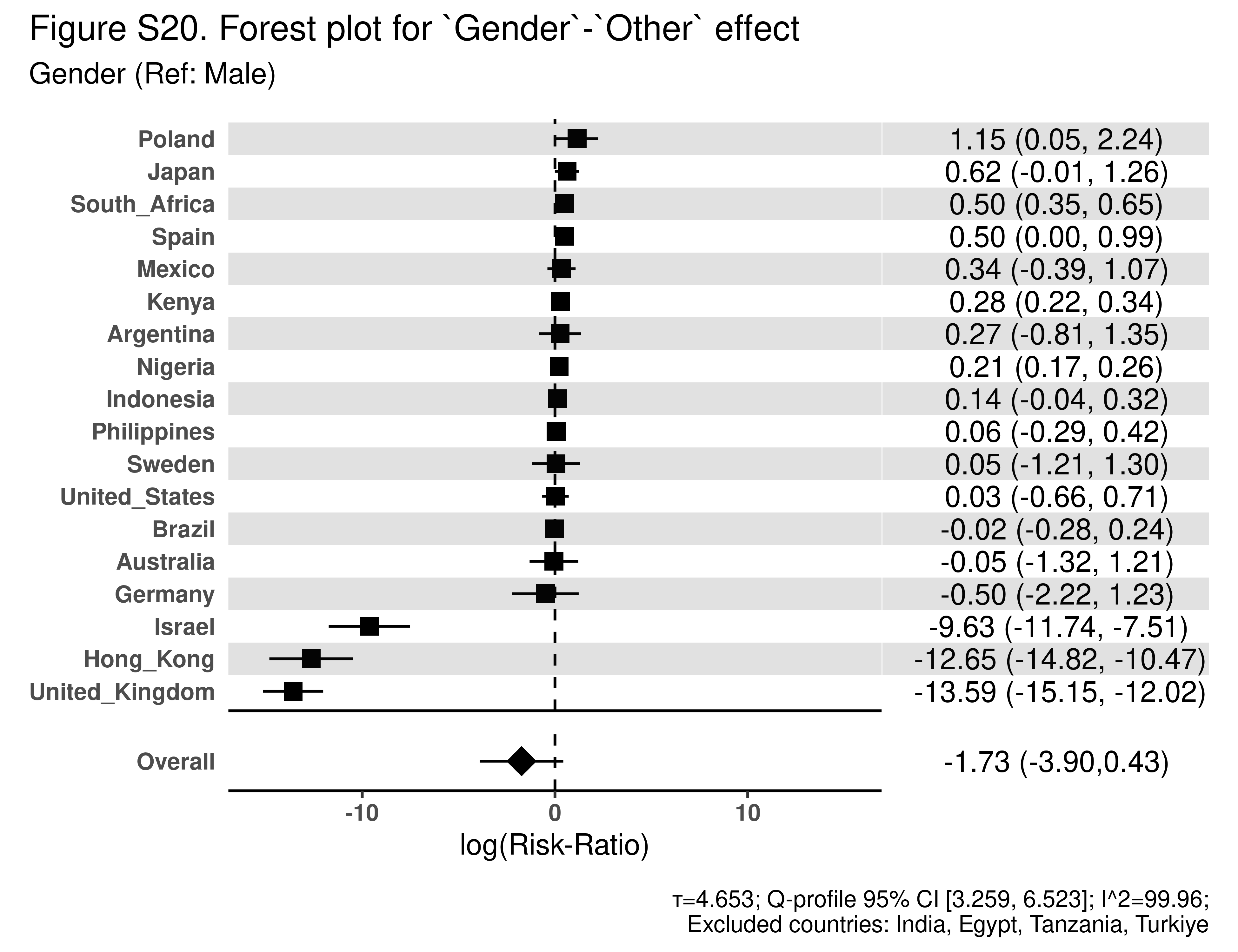

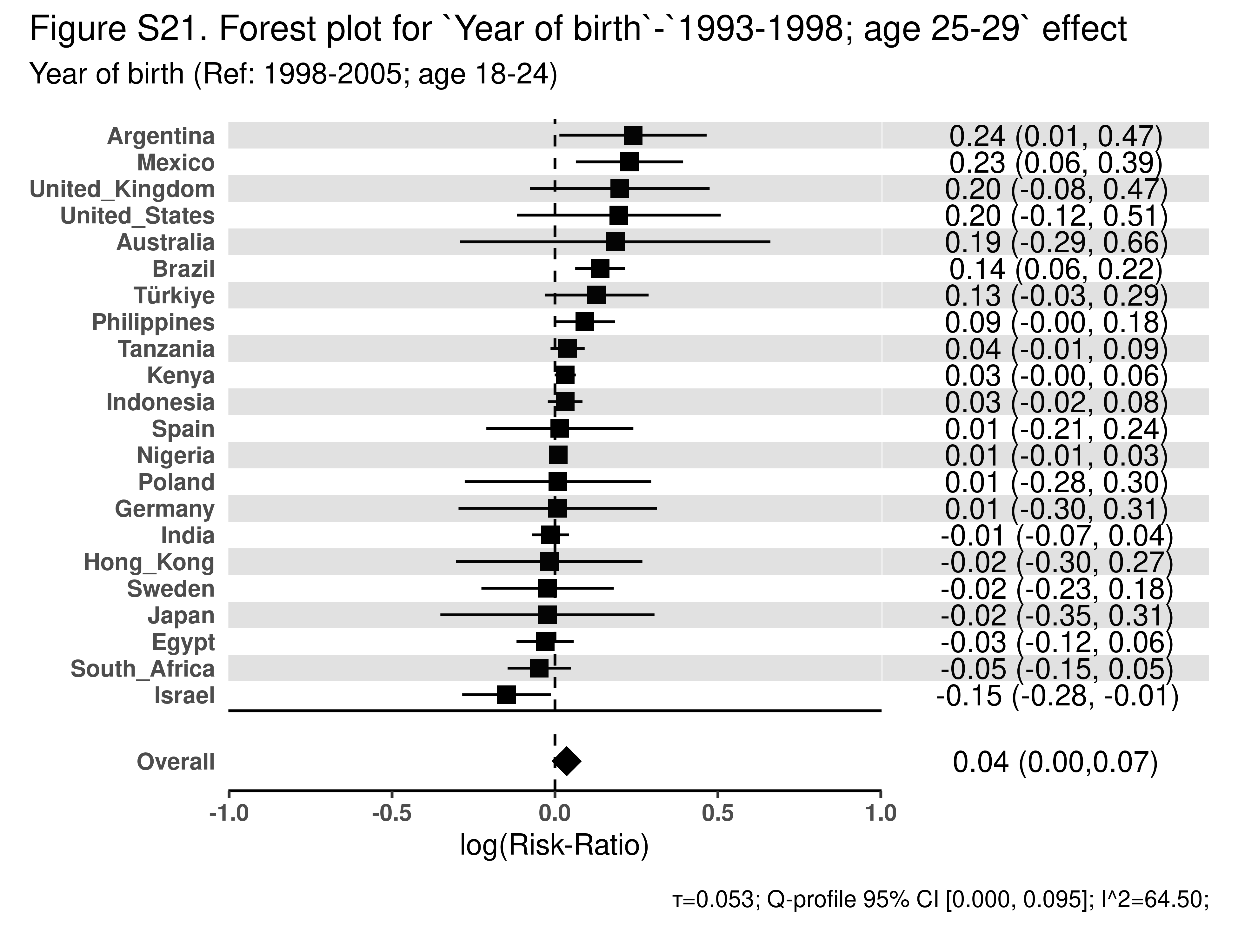

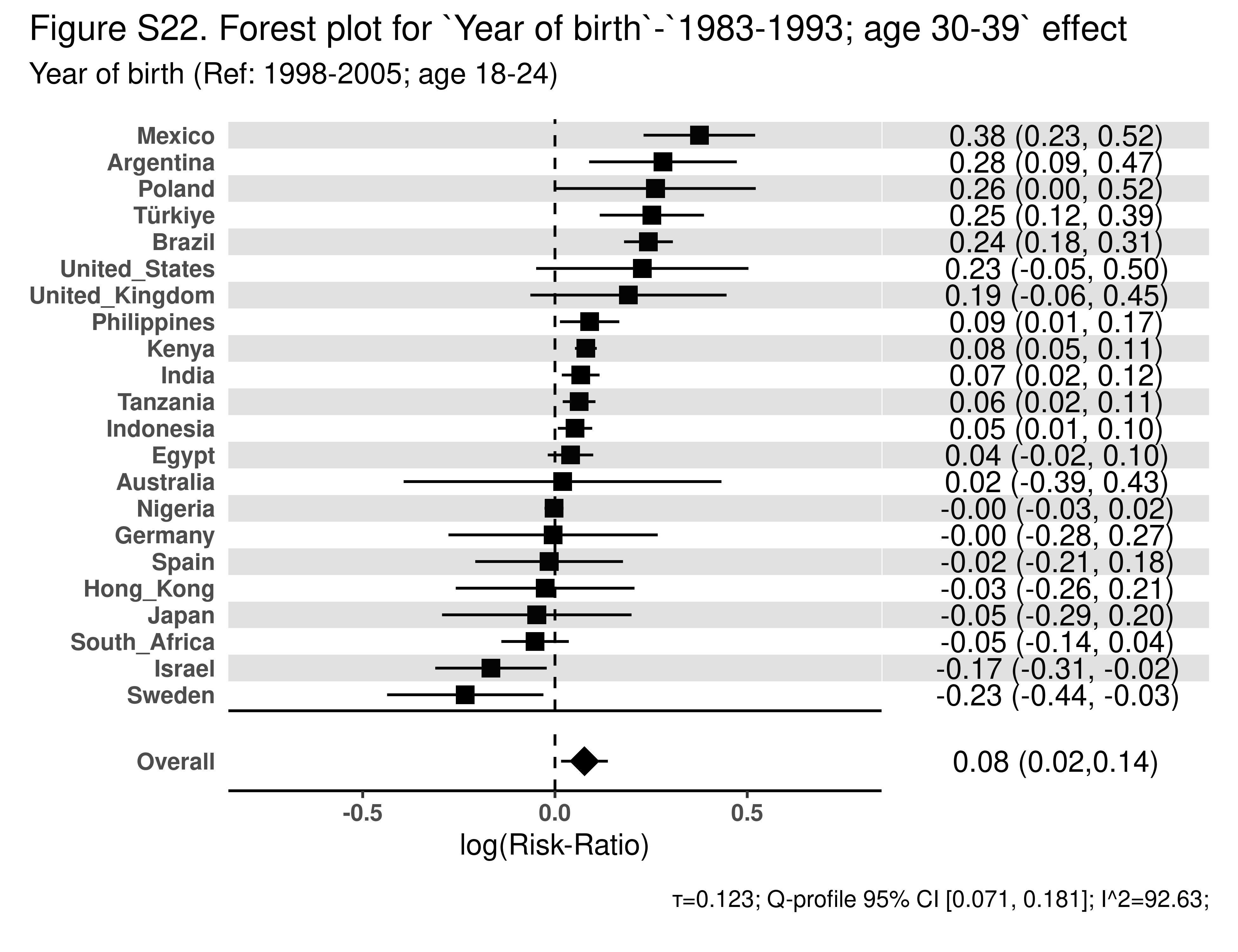

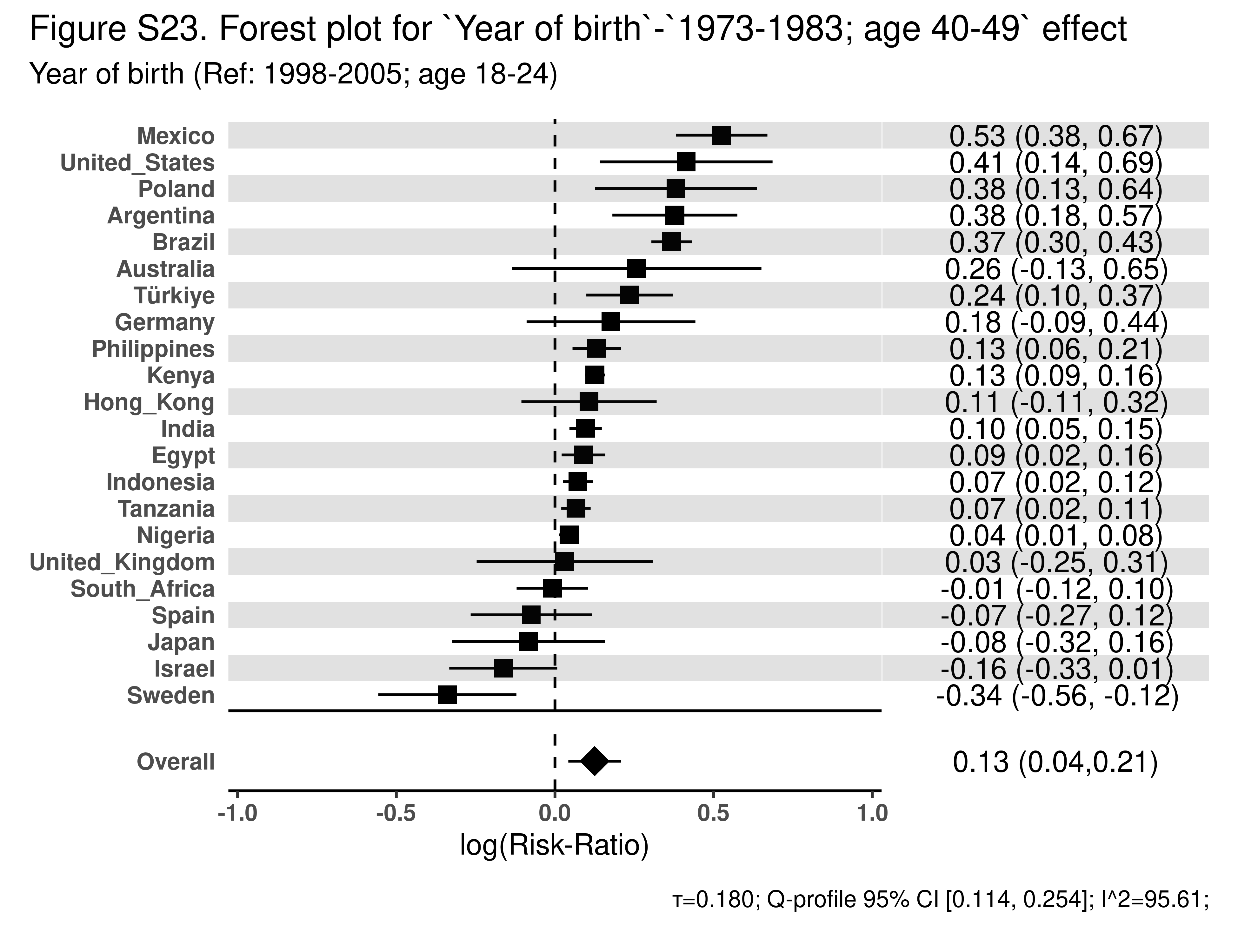

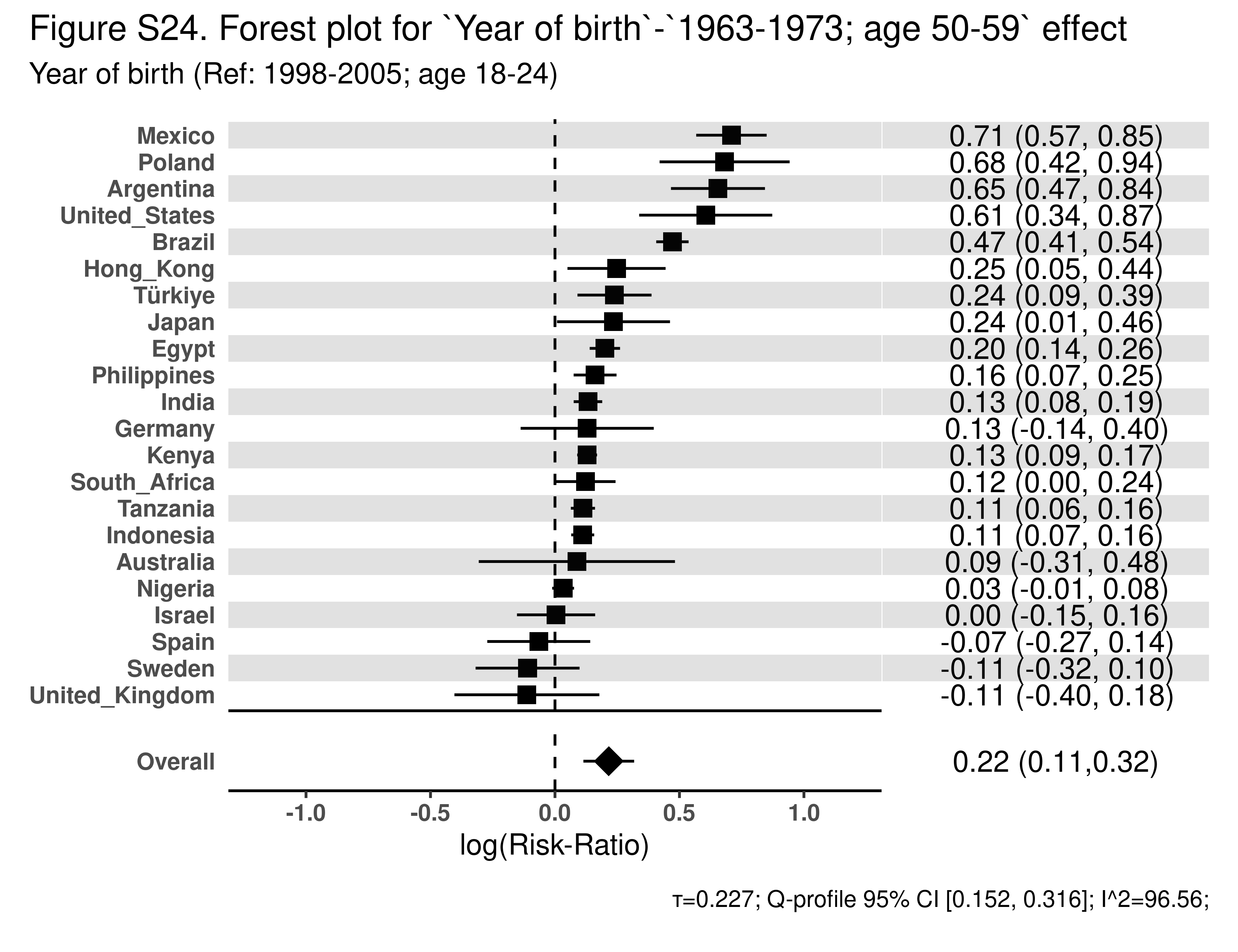

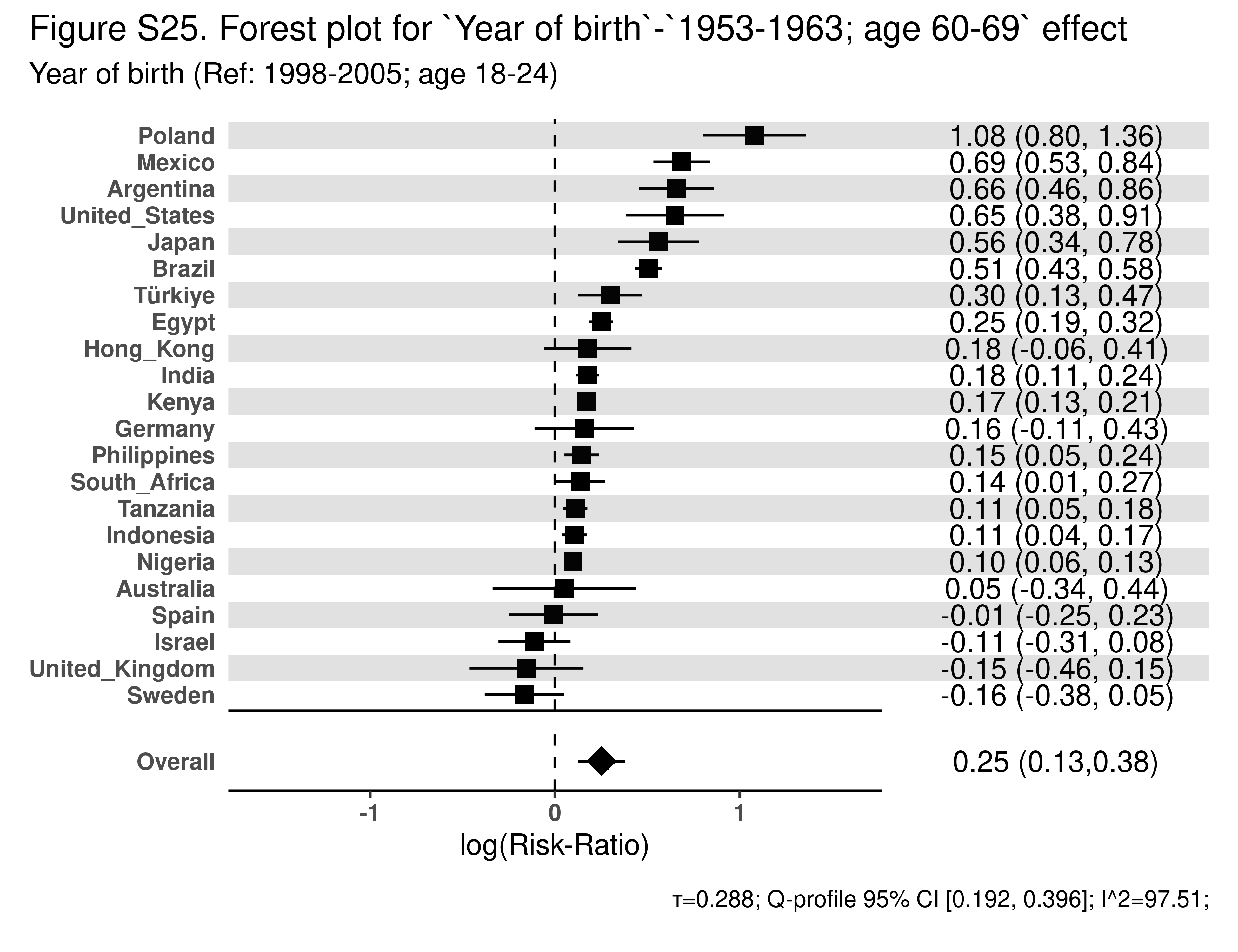

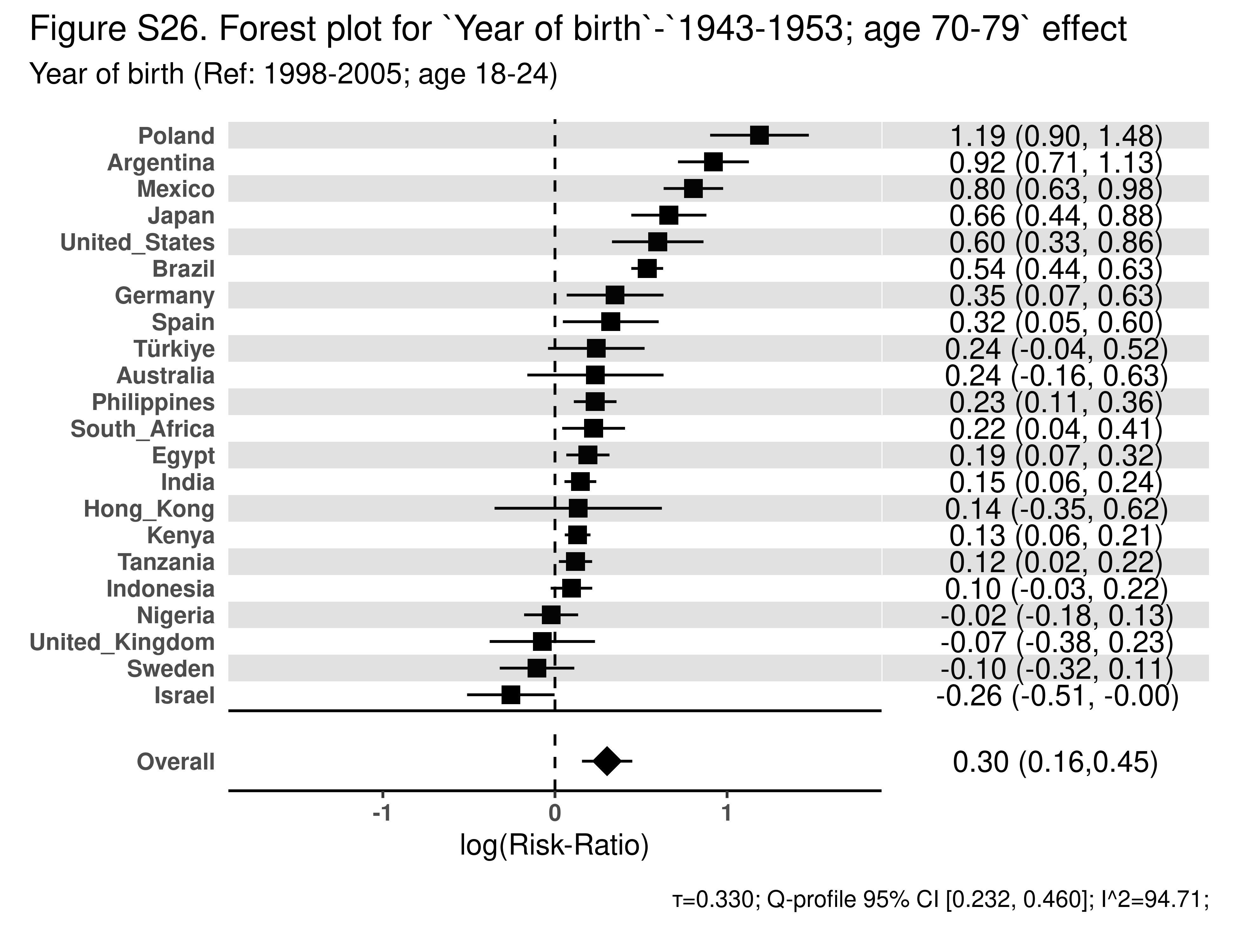

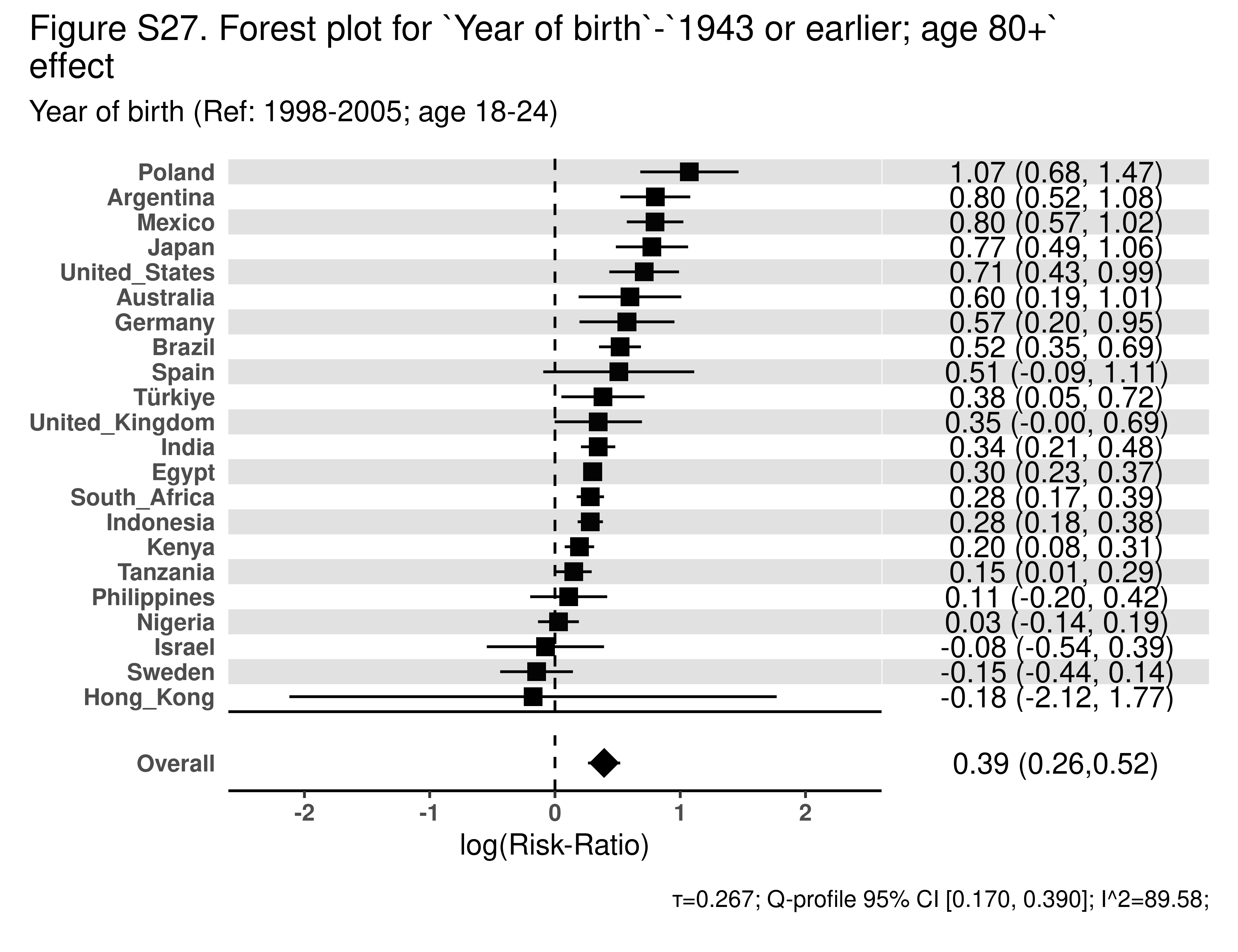

Supplement: Supplementary file 1 — Supplementary Material 1 [file 41598_2025_99796_MOESM1_ESM.docx]
